# Adaptation of global One Health evaluation framework to municipal levels in Fukuoka, Japan

**DOI:** 10.1186/s40249-025-01380-y

**Published:** 2025-11-13

**Authors:** Fumihiko Yokota, Yi-Sheng Ning, Shu-Ning Chen, Hisako Nomura, Shunsuke Managi, Jing-Shu Liu, Xiao-Xi Zhang, Xiao-Nong Zhou, Shuji Shimizu

**Affiliations:** 1 Institute for Asian and Oceanian Studies, Kyushu University, 744 Motooka, Nishi, Fukuoka, 819-0395 Japan; 2 College of Economics & Management, Northwest A and F University, Shaanxi, China; 3 Graduate School of Engineering, Kyushu University, Fukuoka, Japan; 4 School of Global Health, Chinese Center for Tropical Diseases Shanghai Jiao Tong University, Shanghai, China; 5 Faculty of Agriculture, Kyushu University, Fukuoka, Japan

**Keywords:** One Health, Global One Health Index, Municipal health assessment, Fukuoka Prefecture, Health systems evaluation, Japan

## Abstract

**Background:**

The One Health approach recognizes the interconnectedness of human, animal, and environmental health to address global health threats. While the global One Health Index (GOHI) has been applied nationally, its adaptation to sub-national contexts remains unexplored. This study aimed to adapt GOHI to construct localized indicators for Fukuoka, Japan, and assess One Health implementation across municipalities.

**Methods:**

The research followed a three-phase approach: (1) Indicator Selection, where 34 indicators were selected from GOHI and Fukuoka One Health Promotion Action Plan through expert consultation; (2) Data Collection and Score Standardization using robust scaling methods; and (3) Weight Determination using Fuzzy Analytic Hierarchy Process. Fukuoka One Health Index (FOHI) scores were computed and analyzed using descriptive statistics and Latent Class Analysis (LCA).

**Results:**

The mean FOHI score was 52.27 (range: 41.01–63.71), with the lowest average score in Core Drivers Index (47.11) compared to Internal Drivers Index (59.17) and External Drivers (50.43). Municipalities performed strongest in zoonotic disease management (72.33) but weakest in One Health governance (6.36). Miyama City achieved the highest overall score, demonstrating strong governance and integrated implementations. LCA identified two municipal classes differentiated by External Drivers Index scores with clear geographic clustering.

**Conclusions:**

This study demonstrated the feasibility of adapting GOHI to municipal settings and revealed significant variation in One Health implementation across Fukuoka’s municipalities. Performance gaps were identified, particularly in One Health governance despite strong health infrastructure. The methodology offers a blueprint for similar adaptations globally, potentially accelerating the operationalization of One Health principles in local governance contexts.

**Supplementary Information:**

The online version contains supplementary material available at 10.1186/s40249-025-01380-y.

## Background

The One Health approach recognizes the balanced and optimized interconnectedness of human, animal, and environmental health, promoting a transdisciplinary framework for addressing global health threats [1–3]. This integrated approach has gained prominence in confronting escalating challenges such as zoonotic diseases, antimicrobial resistance (AMR), climate change, and food safety [1–4]. The World Health Organization (WHO) estimates that zoonoses are responsible for about one billion cases of illness and millions of deaths annually [5]. The COVID-19 pandemic exemplifies this burden, with estimates of 14.8‒18.2 million deaths from January 2020 to December 2021 [6, 7]. Similarly, AMR, if left unaddressed, is projected to cause approximately 1.91 million deaths annually by 2050, with an additional 8.22 million linked to drug-resistant infections [8]. These data emphasize the urgent need for strengthening public health preparedness through the operationalization of the One Health approach to establish resilient and sustainable health system [9].

To achieve this, systematic evaluation of One Health implementation is essential and requires robust assessment tools capable of monitoring risks and vulnerabilities across various geographic and administrative scales [10, 11]. Despite numerous existing evaluation frameworks, few offer comprehensive monitoring of the complex inter-relationships between the health of humans, animals, and ecosystems [11, 12]. The “global One Health Index” (GOHI), developed in 2022, addresses this gap as the first comprehensive assessment framework evaluating multiple dimensions of One Health across over 160 countries and territories [13–15]. The GOHI framework comprises three main categories: External Drivers Index (EDI), assessing socio-economic, demographic and environmental factors; Internal Drivers Index (IDI), evaluating health services and infrastructure; and Core Drivers Index (CDI), measuring One Health implementation and practices [13–15]. These categories are further divided into 13 key indices, 57 indicators, and 170 sub-indicators, providing a detailed evaluation structure for identifying vulnerabilities and prioritizing interventions [13–15].

Although the GOHI has been successfully applied in assessments at global- and national-levels in China and Sub-Saharan Africa [16–19], its adaptation to sub-national administrative units remains unexplored. This represents a critical gap, as effective One Health implementation requires complementary top-down and bottom-up approaches [20–22]. Empirical evidence demonstrates that local administrative engagement and community participation are essential elements for sustainable One Health initiatives [23, 24], highlighting the need for evaluation frameworks applicable at municipal levels.

Fukuoka Prefecture in northern Kyūshū, Japan, is an ideal setting to examine municipal-level One Health implementation. As a national pioneer in formalized One Health governance, Fukuoka enacted Japan’s first One Health Ordinance in 2020 and introduced 17 distinct initiatives across its municipalities [25–27]. With a population of over 5 million, the prefecture includes major urban centers (Fukuoka, Kitakyushu, Kurume) as well as rural and coastal areas that support agriculture, fisheries, forestry, and livestock industries [28, 29]. This diversity makes Fukuoka a valuable case for exploring One Health across varied socioeconomic and environmental contexts.

Fukuoka’s leadership in One Health began with the “Fukuoka Declaration” at the 2nd World Veterinary Association Congress in 2016, followed by the launch of the Fukuoka One Health Promotion Action Plan (FOHPAP) in March 2022 [25, 30–32]. FOHPAP outlines seven policy pillars: (1) zoonosis control, (2) AMR countermeasures, (3) environmental protection, (4) coexistence of people and animals, (5) health promotion, (6) human-animal-environment relationships, and (7) infrastructure for One Health [25]. Fukuoka is also unique among Japan’s 47 prefectures in establishing a dedicated One Health Promotion section within its Health and Medical Care Department, working collaboratively with departments for Citizen Life, Environment, and Agriculture, Forestry, and Fisheries [25, 33, 34].

By April 2025, 38 of Fukuoka’s 60 municipalities had declared themselves as “One Health promotion municipalities” [33]. Initiatives include integrated education from primary school to university, a registration system for One Health business contractors, and certification of local agricultural, fishery, and livestock products [33, 34]. However, the 20 indicators currently used under FOHPAP are limited to the prefectural level and are not tailored for municipal assessment, leaving a gap in understanding localized implementation.

Given that municipalities are the frontline of One Health practice—where policies are enacted and services delivered—there is a critical need for standardized tools to evaluate their performance. Adapting the GOHI to the municipal level in Fukuoka offers a structured, data-driven framework to assess local implementation, identify strengths and gaps, and support more effective, evidence-based decision-making.

This study addresses two primary research questions: (1) how effective can the GOHI framework be adapted to construct localized indicators in Fukuoka? and (2) what is the current status of One Health implementation across Fukuoka’s municipalities regarding governance, zoonotic diseases, food security, AMR, climate change, and supporting infrastructure? By adapting the GOHI framework to municipal settings, this research aims to construct an operational local assessment tool as “Fukuoka One Health Index (FOHI)” with potential applications in other municipalities. The findings provide evidence-based insights for local governments to evaluate One Health practices, facilitate inter-municipal collaboration, and strengthen governance capacity for effective resource allocation and policy formulation. Comprehensive evaluation of One Health at the local level is essential to enhancing health outcomes across all communities—particularly those with limited resources—and to advancing more equitable and inclusive approaches to public health. Despite the shift in scale, GOHI’s comprehensive and modular structure offers a robust conceptual foundation for sub-national adaptation while ensuring alignment with national and international One Health standards. This integrated approach combining top-down framework adaptation with bottom-up assessment contributes to building more comprehensive and resilient One Health systems capable of addressing future global health challenges at community levels where implementation ultimately occurs.

## Methods

**Fig.1:**
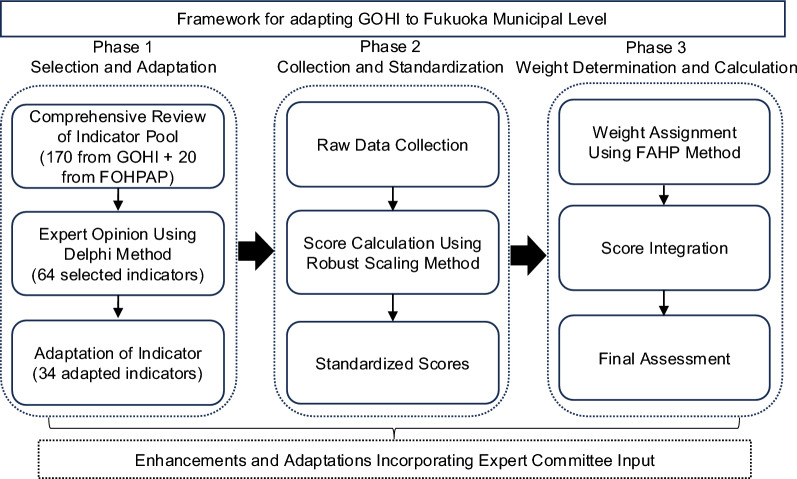
Flowchart of GOHI Adaptation for Construction of FOHI. *GOHI* Global One Health Index, *FOHPAP* Fukuoka One Health Promotion Action Plan, *FAHP* Fuzzy Analytic Hierarchy Process

The research design framework of adapting GOHI to Fukuoka municipal level for constructing FOHI consists of three phases: Phase 1 (Indicator Selection & Adaptation), Phase 2 (Data Collection & Score Standardization), and Phase 3 (Weight Determination & Score Calculation) (Fig. 1).

### Phase 1: indicator selection and adaptation

In Phase 1, we began with a thorough review and analysis of the comprehensive pool of both GOHI 170 sub-indicators and 20 measurable indicators from FOHPAP [13–15, 25]. The GOHI sub-indicators were evaluated based on selection criteria (Table 1). Data and information were thoroughly searched, reviewed, and assessed from the following sources: e-Stat (Comprehensive portal site for Japan government statistics), FOHPAP, Fukuoka Prefectural One Health portal site, Fukuoka One Health Official Annual Reports, Fukuoka Prefecture website, and Fukuoka Prefecture open data site. [25, 33–37].

**Table 1:** Data selection criteria to select FOHI from GOHI and FOHPAP indicator pool

Principle	Criteria
Availability of Municipal-level Data	Data should be available at the municipal-level and/or similar levels such as local data bureaus or local data points
Relevance/equivalence	Data should represent and/or equivalent to the content of corresponding indicators in GOHI framework and FOHPAP 7 pillars
Authoritative sources	Data is retrieved from authoritative country, prefectural, municipality agencies
Completeness	Data used for indicators should cover a sufficient proportion of Fukuoka prefecture
Timeliness	Data should cover a recent temporal period (2020–2024) and be updated at least annually
Comparability	For single indicators, data should be measured with an established and unified method

*FOHI* Fukuoka One Health Index, *GOHI* Global One Health Index, *FOHPAP* Fukuoka One Health Promotion Action Plan

The initial selection of potentially suitable indicators was made from the GOHI and FOHPAP indicator pool. Indicators were finally determined from these initially selected indicators based on multiple expert consultations and validation using the Delphi method, which is a structured forecasting technique that gathers expert opinions, refining responses iteratively to achieve consensus [38].

### Phase 2: data collection and standardization

In Phase 2, we collected data from the sources listed in Table 2. Most indicators were derived from national and Fukuoka Prefectural Official databases, supplemented by internal data obtained from the Fukuoka Prefectural Office.

**Table 2:** Data sources utilized for the construction of FOHI

Index categories	Data sources
External Drivers Index (EDI)	Portal site of official statistics of Japan (e-Stat) [35], Ministry of Land, Infrastructure, Transport and Tourism [39], Fukuoka Prefecture Government [40], Ministry of Internal Affairs and Communications [41], Agency for Natural Resources and Energy [42], Fukuoka Prefecture Government Internal Data^a^
Internal Drivers Index (IDI)	Fukuoka Prefecture Website [36], Fukuoka Prefecture Government Internal Data^a^, Ministry of Health, Labour and Welfare [43]
Core Drivers Index (CDI)	Fukuoka Prefecture One Health Portal Site [33], Fukuoka One Health Certification Website [44], Fukuoka One Health Declaration Business Search [45], Miyama City One Health Special Site [46], Fukuoka Prefecture Website [36], Portal site of official statistics of Japan (e-Stat) [35], Fukuoka Infectious Disease Surveillance Weekly Report [47], Ministry of Agriculture, Forestry, and Fisheries [48], Fukuoka Agriculture, Forestry, and Fisheries Support Group [49], JANIS (Japan Nosocomial Infections Surveillance [50], Ministry of the Environment [51], Heat Illness Prevention Information [52], Fukuoka Prefecture Government Internal Data^a^

^a^The internal data from the Fukuoka Prefecture Government is accessible only in hard copy. FOHI Fukuoka One Health Index

The collected data for each indicator underwent rigorous standardization using the robust scaling method to address the heterogeneous nature of municipal-level data and potential outliers [53, 54]. This initial transformation centers the data around the median and scales it based on the interquartile range. The data was then linearly re-scaled to a uniform 0–100 scale for score standardization, while maintaining the relative relationships between municipalities.

Only two indicators had missing values. For the indicator A1.2 in Table 6, there are 32 water quality data monitoring points in 17 municipalities in Fukuoka prefecture. Therefore, the mean value from these 17 municipalities were imputed for missing in the remaining 43 municipalities. For the indicator C5.3 in Table 6, there are only 12 municipal observational monitoring sites. Therefore, the same imputation method as the indicator A1.2 was used for the indicator C5.3.

The robust scaling method is expressed by Eq. (1):

$$\begin{array}{c}{X}_{\text{scaled}}=\frac{X-{\text{median}}\left(X\right)}{{\text{IQR}}\left(X\right)}\times 100\#\end{array}$$

where X represents the original value, median(X) is the median of the indicator across all municipalities, and IQR(X) is the interquartile range. This transformation centers the data and scales it based on the interquartile range, making it less sensitive to extreme values. After this, we designated the highest observation value (ranked first among all 60 municipalities) as the optimal value, and the lowest observation value (ranked last among all municipalities) as the worst value to be standardized. We then linearly re-scaled the standardized data to ensure each indicator’s score falls within a uniform range of 0–100 points. For indicators where higher values indicate poorer performance, we applied reverse scoring after standardization to maintain interpretive consistency, ensuring higher scores uniformly represent better performance across all metrics. We deliberately avoided applying Min–Max normalization directly to the raw data as used in GOHI, as such an approach is susceptible to distortion from extreme outliers [55]. Instead, we first applied robust scaling to reduce the influence of outliers before performing the Min–Max normalization as a second step on the already robust-scaled data. For binary indicators (Yes/No), we assigned scores of 100 and 0 respectively. For ordinal indicators, we applied proportional scoring (e.g., 0, 33.3, 66.7, and 100 for the four levels). For temporal consistency and data reliability, we established 2022 as our baseline year, aligning with the implementation of the FOHPAP. To address missing data points, we conducted multiple imputation procedures using contextually relevant variables identified through rigorous expert consultation processes.

### Phase 3: weight determination and score calculation

In Phase 3, we determined “weighing” for each 3 categories and 13 key indices using the Fuzzy Analytic Hierarchy Process (FAHP), which incorporates fuzzy logic to handle uncertainty and imprecision in decision-making by using fuzzy pairwise comparisons to derive more reliable priority weights [56]. Equal weights were assigned to the 34 different measurement indicators within the same key indicator, according to the consensus from 23 One Health experts (Table 2). All 23 experts participated in a questionnaire survey which included 23 series of pairwise questions to compare “which of 3 categories and 13 key indices are more important”. Based on the results of each pairwise question, the relative importance of 3 categories and 13 key indices were determined at different hierarchical levels. Results from expert evaluations were systematically collected through repeated pairwise comparisons for each pair of indicators from 23 experts, transforming qualitative judgments into quantitative ratios [57].

To systematically aggregate the binary judgments from all experts, we constructed fuzzy judgment matrices for each hierarchical level. For each pairwise comparison between indicators i and $j$, we counted the number of experts who preferred indicator $i$ over indicator $j$, denoted as ${a}_{ij}$. This value could range from 0 to 23, reflecting the strength of collective expert preference. The reciprocal relationship between matrix elements was maintained as ${a}_{ji}$ = 23/${a}_{ij}$, ensuring mathematical consistency in the preference structure. This approach effectively converted binary judgments into a quantitative framework that captured both the direction and strength of collective expert preferences.

The aggregated judgment matrices were constructed at multiple hierarchical levels: (1) a top-level matrix comparing the three main categories (EDI, IDI, CDI), (2) a matrix for the five key indices within EDI, (3) a matrix for the three key indices within IDI, and (4) a matrix for the five key indices within CDI. For each matrix, we calculated the Consistency Ratio (CR) to verify judgment consistency. The CR values were 0.026, 0.081, 0.068 for the top-level, EDI, and IDI matrices respectively, all well below the strict threshold of 0.1, while the CDI matrix had a CR value of 0.131, which exceeded the stringent standard but remained below the secondary threshold of 0.15. This indicates that expert judgments maintained satisfactory consistency across all hierarchical levels, with most matrices demonstrating high consistency under the rigorous criterion.

The weight vector for each level was calculated using the geometric mean method, which is particularly suitable for fuzzy hierarchical analyses due to its ability to minimize the influence of extreme values and maintain ratio scale properties. For each indicator $i$, the weight was calculated as:

$${W}_{i}=\frac{{\left(\prod_{j=1}^{n} {a}_{ij}\right)}^{1/n}}{\sum_{k=1}^{n} {\left(\prod_{j=1}^{n} {a}_{kj}\right)}^{1/n}}$$

where ${W}_{i}$ represents the normalized weight of indicator $i$; ${a}_{ij}$ represents the fuzzy preference value of indicator $i$ over indicator $j$ (transformed to a 0–1 scale by dividing by the total number of experts); $n$ is the total number of indicators being compared.

This weighting methodology was applied across all hierarchical levels of our assessment framework. For the system component level, the comparisons revealed varying degrees of relative importance. The final One Health performance score for each municipality was calculated through a hierarchical aggregation process that integrates the standardized indicator scores with their corresponding FAHP-derived weights. The calculation follows a bottom-up approach, progressively combining scores from the 34 measurement indicators to 13 main indices, and finally to the three index categories.

$$KI=\sum ({X}_{i}\times {w}_{i})$$

where ${X}_{i}$ represents the standardized score for measurement indicator $i$, and ${w}_{i}$ represents its corresponding weight derived from the FAHP process. Based on expert recommendations, measurement indicators under the same Key Indicator were assigned equal weights in this study.

The Category scores (CS) are then computed by aggregating their respective Key Indicators:

$$CS=\sum (K{I}_{j}\times w{k}_{j})$$

where $K{I}_{j}$ represents the score of Key Indicator $j$, and $w{k}_{j}$ represents the weight assigned to that key indicator within its category.

Finally, the overall One Health score (OHS) for each municipality is calculated by combining the three Categories:

$$OHS=(EDI\times {w}_{1})+(IDI\times {w}_{2})+(CDI\times {w}_{3})$$

where EDI, IDI, and CDI represent the scores for the External Drivers Index, Internal Drivers Index, and Core Drivers Index respectively, and w₁, w₂, w₃ represent their corresponding weights in the overall assessment framework. This hierarchical aggregation ensures that the final score reflects both the relative importance of different components and their interconnected relationships within the One Health system.

### Final analysis and assessment

After calculating One Health scores for 60 municipalities in Fukuoka Prefecture, we employed various statistical methods to analyze the distribution of these scores. Common statistical approaches including descriptive statistics, box plots, and violin plots were utilized to examine the overall level of One Health governance and internal variations across the prefecture. Beyond standard statistical methods, we constructed spatial distribution maps of the scores and created bar charts of the top 20 municipalities in each of the three index categories. These additional analyses enabled a more detailed examination of regional differences, assessed the applicability of our indicator system at the municipal level, and provided scientific evidence for developing strategies to enhance One Health performance throughout Fukuoka Prefecture.

**Fig. 2:**
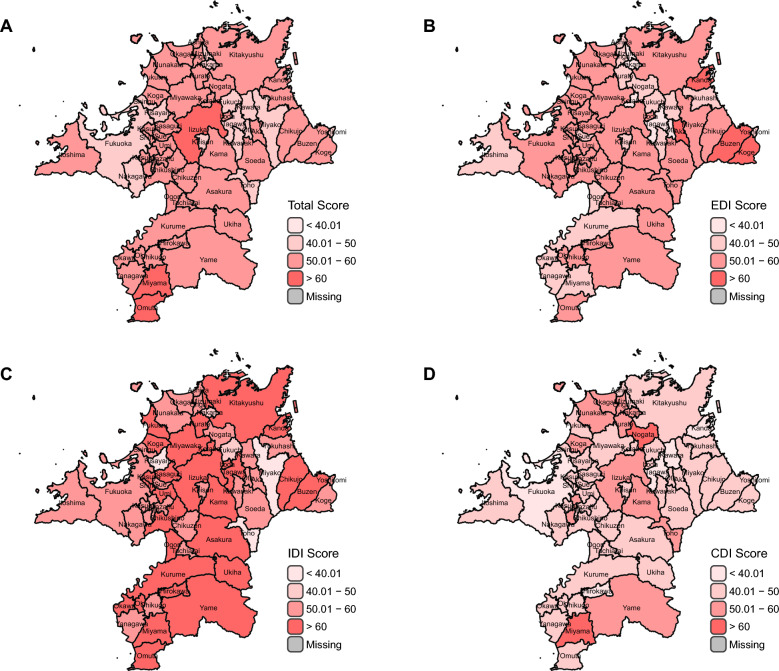
FOHI Scores among 60 municipalities in Fukuoka by Total **A**, EDI **B**, IDI **C**, and CDI **D** categories. Created based on National Land Numerical Information (Administrative Boundary Data), Ministry of Land, Infrastructure, Transport and Tourism, Japan (https://nlftp.mlit.go.jp/ksj/gml/datalist/KsjTmplt-N03-2025.html), retrieved on November 15, 2024. FOHI Fukuoka One Health Index, EDI External Drivers Index, IDI Internal Drivers Index, CDI Core Drivers Index

Additionally, we employed Latent Class Analysis (LCA) [58] to identify different One Health performance patterns among 60 municipalities in Fukuoka Prefecture. This analytical approach was guided by our hypothesis that geographically neighboring municipalities might exhibit similar patterns in One Health performance due to various forms of inter-municipal influence, including knowledge sharing, policy diffusion, shared environmental conditions, and collaborative initiatives. We assumed that each municipality belongs to one of n latent classes based on their scoring patterns across the three core indices: EDI, IDI, and CDI. To facilitate the analysis, we categorized the scores of these three core indices into four distinct score groups; “below 40.01,” “40.01–50,” “50.01–60,” and “above 60” as shown in Fig. 2. Using a data-driven approach, LCA determined the number of existing latent classes based on the distribution patterns of municipalities across the three core indices’ score groups. The analysis also calculated the posterior probability of each municipality belonging to each latent class, with the class having the highest posterior probability considered as the municipality’s latent class membership. The optimal number of latent classes was determined by the model with the lowest Bayesian Information Criterion (BIC) value among models containing 2–6 latent classes.

All statistical analyses and data visualizations were performed using R software version 4.2.1 (R Foundation for Statistical Computing, Vienna, Austria) [59]. The hierarchical fuzzy analytic hierarchy process (FAHP) was implemented using custom R scripts based on the algorithm described in the method section. Weight calculations, consistency ratio evaluations, and matrix manipulations were performed using the base R functions. For data visualization, the ggplot2 package [60] was utilized to create figures displaying weight distributions and comparative analyses across municipalities.

## Results

### Indicator selection, expert validation, and internal consistency

The initial selection of 64 potentially suitable indicators, and then 34 indicators were finally determined based on multiple expert consultations. The composition of expert advisory committee detailed in the Table 3.

**Table 3:** Characteristics of the expert advisory committee assisting with the adaptation of GOHI to Fukuoka (*n* = 23)

Item	Category	Counts	Percentage (%)
Gender	Female	9	39.1
	Male	14	60.9
Age category	21–30	1	4.3
	31–40	3	13.0
	41–50	8	34.8
	51–60	8	34.8
	61 +	3	13.0
Research area	Human medicine, nursing, public health, pharmacy	8	34.8
	Veterinary Science	3	13.0
	Environmental Science	3	13.0
	Agriculture	2	8.7
	Psychology	2	8.7
	Economy/management	2	8.7
	Others	3	13.0
Work experience	0–9	5	21.7
	10–19	8	34.8
	20 +	10	43.5

*GOHI* Global One Health Index

These selected 34 indicators maintained structural consistency to correspond with the GOHI core framework, which includes three categories of the EDI, IDI, CDI and 13 key indices (Table 4). To better reflect Fukuoka’s local context, we slightly modified and adapted GOHI sub-indicators to be practical and measurable to the Fukuoka municipal level based on three rounds of consultations with 23 senior experts from Kyushu University and Fukuoka Prefectural Office. This ensured both scientific rigor and local applicability to 7 policy pillars of FOHPAP. The demographic characteristics of the expert advisory committee members are detailed in Table 3, with 60.9% males and the majority (82.6%) aged 41 or older. The experts represent diverse fields including human medicine, nursing, public health, pharmacy (34.8%), veterinary science (13.0%), environmental sciences (13.0%), agriculture (8.7%), psychology (8.7%), and economy/management (8.7%). Additionally, 43.5% of them had more than 20 years of relevant work experience.

**Table 4:** GOHI’s 3 index categories and 13 key indices

Index categories	Key indices
1. External Drivers Index	1. Earth systems
	2. Institutional systems
	3. Economic systems
	4. Social systems
	5. Technological systems
2. Internal Drivers Index	6. Human health
	7. Animal health and ecosystem diversity
	8. Environmental resources
3. Core Drivers Index	9. One health governance
	10. Zoonotic diseases
	11. Food security
	12. Antimicrobial resistance (AMR)
	13. climate change

*GOHI* Global One Health Index

To assess the internal consistency and reliability of the FOHI framework, we conducted Cronbach’s alpha analysis. We focused primarily on evaluating the reliability of the three main categories (EDI, IDI, CDI) in relation to the overall FOHI score, as this represents the most critical structural relationship in our assessment framework. The Cronbach’s alpha for this level was 0.62 (standardized α = 0.63), which indicates an acceptable level of internal consistency for a newly developed composite index in the field of public health and policy assessment [61]. This finding supports the structural integrity of the FOHI’s three-category framework while acknowledging the inherent complexity and multidimensionality of One Health implementation across diverse municipal contexts.

We intentionally did not conduct reliability analysis at the lower hierarchical levels (within each category) because these components were deliberately designed to capture distinct and complementary aspects of One Health implementation rather than to measure the same underlying construct. In multidimensional assessment frameworks like FOHI, it is methodologically appropriate to prioritize content validity and comprehensive coverage of the conceptual domain over high internal consistency at all levels [62]. This approach ensures that the index captures the full breadth of One Health implementation while maintaining acceptable reliability at the primary structural level.

### Weight Distribution Across Indices and Categories

Table 5 displays the weight information for the 3 categories and 13 key indices. We found that among the 3 categories, the CDI had the highest weight (40.9%), followed by IDI (36.6%) and EDI (22.5%). Within EDI, the percentages of weight for key indices were highest in Earth System (24.2%), followed by Social Systems (24.1%), Institutional Systems (21.7%), Technological Systems (16.8%), and Economic Systems (13.2%). Within IDI, more than half of the weight (53.1%) was allocated to Human Health, followed by Animal Health and Ecosystem Diversity (26.9%) and Environmental Resources (20.0%). Finally, within CDI, the highest weight percentage was for One Health Governance (28.6%), followed by Zoonotic Diseases (22.2%), Food Security (21.4%), Climate Change (14.4%), and AMR (13.4%).

**Table 5:** Calculated weights of one health index system for fukuoka prefecture

Index categories	Weight (%)	Key indices	Weight (%)
External Drivers Index	22.5	Earth systems	24.2
		Institutional systems	21.7
		Economic systems	13.2
		Social systems	24.1
		Technological systems	16.8
Intrinsic Drivers Index	36.6	Human health	53.1
		Animal health and ecosystem diversity	26.9
		Environmental resources	20.0
Core Drivers Index	40.9	One health governance	28.6
		Zoonotic diseases	22.2
		Food security	21.4
		Antimicrobial resistance (AMR)	13.4
		Climate change	14.4

### Localized FOHI indicators and municipal-level data overview

Table 6 presents the localized 34 FOHI which were constructed by adapting GOHI’s 3 index categories and 13 key indices. Data for indicator A1.2 were not available in all municipalities but 32 water quality data monitoring points at 17 different municipalities across Fukuoka prefecture. Therefore, the mean value from these 17 municipalities were imputed for missing in the remaining 43 municipalities. Similarly, the data for indicator C5.3 were available from 12 municipal observational monitoring sites across Fukuoka prefecture. Therefore, the same imputation method as the indicator A1.2 was used for the indicator C5.3.

**Table 6:** Municipal level’s 34 FOHI based on GOHI’s 3 index categories and 13 key indices

Index categories	key indices	Fukuoka one health indicator (FOHI)	Year of data collected
A External Drivers Index	A1 Earth systems	A1.1 Forest area (% of total land area)	2020
		A1.2 Biochemical Oxygen Demand level (mg/L) ^a^1	2022
	A2 Institutional systems	A2.1 Government revenue (% of GDP)	2022
		A2.2 Municipal regulations for pet evacuation during disasters (Yes = 1; No = 0)	2024
	A3 Economic systems	A3.1 Unemployment (% of total labor force)	2020
	A4 Social systems	A4.1 Natural population growth rate (%)	2022
	A5 Technological systems	A5.1 Renewable electricity generation (% of total electricity demand)	2022
B Internal Drivers Index	B1 Human health	B1.1 Number of hospital and clinic beds (per 1,000 population)	2021
		B1.2 Number of doctors (per 1,000 population)	2021
		B1.3 Suicide rate (per 10,000 population)	2022
	B2 Animal health and ecosystem diversity	B2.1 Existence of livestock disease surveillance system (Yes = 1; No = 0)	2024
		B2.2 Existence of Wild animal disease surveillance system (Yes = 1; No = 0)	2024
	B3 Environmental resources	B3.1 Waste disposal recycling rate (%) (total amount recycled + amount collected by group) / (total amount treated + amount collected by group) × 100	2022
C Core Drivers Index	C1 One health governance	C1.1 One Health policy agreement in municipal council (Yes = 1; No = 0)	2024
		C1.2 One Health declaration in municipal council (Yes = 1; No = 0)	2024
		C1.3 Number of registered agricultural, forestry, fishery products (per 10,000 population)	2024
		C1.4 Number of Registered One Health declaration businesses implementing One Health Practice (per 10,000 population)	2024
		C1.5 One Health website presence scale (3 = dedicated website; 2 = dedicated webpage; 1 = section on government website; 0 = no web presence)	2024
		C1.6 Number of One Health promotion and/or education facilities within municipalities	2024
	C2 Zoonotic Diseases	C2.1 Influenza vaccination rate for age 65 + (%)	2022
		C2.2 Influenza Weekly Average Number of Cases per Sentinel Surveillance Site^b^2	2022
		C2.3 COVID-19 Weekly Average Number of Cases per Sentinel Surveillance Site^b^2	2022
		C2.4 Infectious Gastroenteritis Weekly Average Number of Cases per Sentinel Surveillance Site*2	2022
		C2.5 Tuberculosis incidence (per 100,000 population) ^b^2	2022
	C3 Food security	C3.1 Food waste per capita (kg)	2022
		C3.2 Good Agricultural Practices (GAP) certified agricultural producers (per 10,000 population)	2024
		C3.3 Food chain inspection implementation rate (%) ^c^3	2022
		C3.4 Arable land per capita (ha)	2022
		C3.5 Local food promotion restaurants (per 10,000 population)	2024
	C4 Antimicrobial resistance (AMR)	C4.1 AMR surveillance system (Yes = 1; No = 0)	2022
		C4.2 Japan Nosocomial Infections Surveillance (JANIS) hospitals (per 10,000 population)	2024
	C5 Climate change	C5.1 CO2 emissions (tons/capita)	2021
		C5.2 CO2 emissions (kg/GDP)	2021
		C5.3 Days with WBGT index above 25 (days/year) ^d^4	2022

Note: The values for the FOHI A1.2, A3.1, B1.3, C2.2, C2.3, C2.4, C2.5, C3.1, C5.1, C5.2, C5.3 were revered to maintain that higher values uniformly represent better performance across all indicators. FOHI Fukuoka One Health Index, GOHI Global One Health Index

^a^1 FOHI A1.2 data was collected from 32 water quality data monitoring points in 17 different municipalities in Fukuoka prefecture

^b^2 FOHI C2.2–C3.1 data were collected from twelve (12) public health centers; (1) Kita-Kyushu, (2) Fukuoka, (3) Kurume, (4) Chikushi, (5) Kasuya, (6) Itoshima, (7) Munakata/Onga, (8) Kaho/Kurate, (9) Tagawa, (10) Kita-Chikugo, (11) Minami-Chikugo, (12) Kyochiku

^c^3 FOHI C3.3 data was collected from 9 regional sites; (1) Chikushi, (2) Kasuya, (3) Itoshima, (4) Munakata/Onga, (5) Kaho/Kurate, (6) Tagawa, (7) Kita-Chikugo, (8) Minami-Chikugo, (9) Kyochiku

^d^4 FOHI C5.3 data was collected from 12 observational monitoring sites: (1) Munakata, (2) Yahata, (3) Yukihashi, (4) Izuka, (5) Maebaru, (6) Fukuoka, (7) Dazaifu, (8) Soeda, (9) Asakura, (10) Kurume, (11) Kuroki, (12) Omuta

The data for indicators C2.2, C2.3, C2.4, and C2.5 were only available at 12 regional public health centers which collect the case reports from sentinel surveillance sites such as hospitals or clinics which cover all 60 municipalities. Similarly, the data for indicator C3.3 were available from 9 regional sites which cover all 60 municipalities in Fukuoka prefecture. For these indicators that data are not originally from each municipality but regional sites covering all municipalities, the same data values from the regional sites were allocated to each municipality within the region. The data for the remaining 28 indicators were available at all 60 municipality levels.

Half of the data (17 out of 34 total indicators) were from the year of 2022 as a baseline, but other 17 indicators were not available in 2022, so data from another year between 2020 and 2024 were used (Table 6). Based on the Delphi method, the following 14 indicators were newly changed from GOHI indicators to be adapted to Fukuoka local context; Indicators A2.2, B1.1, B1.2, B2.1, B2.2, C1.1, C1.2, C1.3, C1.4, C1.5, C1.6, C2.4, C3.2, C3.5. The other 20 indicators were derived from the GOHI (Table 6). Only 6 indicators; A2.2, B2.1, B2.2, C1.1, C1.2, C4.1 were dichotomous categories as Yes or No (Table 6). All other data were either numerical values or percentages (proportions or rates).

### Municipal FOHI scores and performance patterns

Table 7 shows the mean of total FOHI score for Fukuoka Prefecture was 52.27 out of 100 full score with a rage from 41.01 to 63.71. The mean scores for Fukuoka Prefecture were highest in IDI (59.17 with a rage from 31.10 to 78.07), followed by EDI (50.43 with a rage from 29.21 to 66.13) and CDI (47.11 with a range from 37.49 to 71.89).

**Table 7:** Summary statistics for FOHI scores for Fukuoka prefecture

Category	Mean	*SD*	Min	Median	Max
Total score	52.27	4.29	41.01	52.33	63.71
EDI	50.43	7.35	29.21	51.03	66.13
IDI	59.17	8.79	31.10	59.64	78.07
CDI	47.11	6.18	37.49	46.32	71.89

Figure 2 shows the FOHI scores among all 60 municipalities in Fukuoka by Total (A) and 3 categories (B–D). In the total score (Fig. 2A), three (3) municipalities scored more than 60, compared with 4 municipalities in EDI (Fig. 2B, 30 municipalities in IDI (Fig. 2C), and only 2 municipalities in CDI (Fig. 2D). The numbers of municipalities with scores between 50–60 were 42 in Fig. 2A, 34 in Fig. 2B, 25 in Fig. 2C, and only 11 in Fig. 2D. These results indicate that while many municipalities in Fukuoka exhibit advanced performance in the IDI and EDI, there remains substantial room for improvement in the CDI. Nonetheless, Miyama achieved scores over 60 in total, CDI, and IDI.

**Fig. 3:**
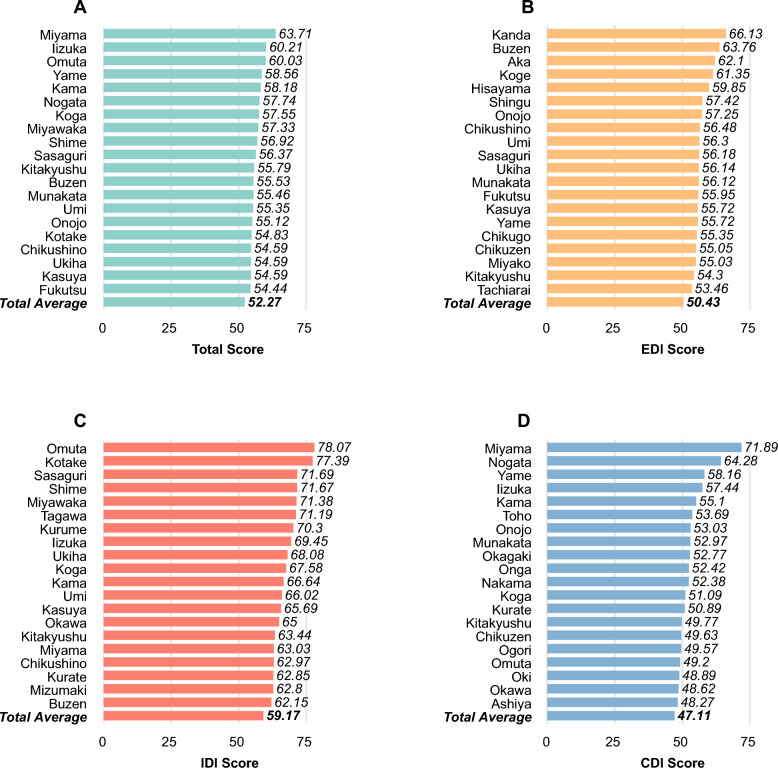
Top-20 Municipalities in FOHI Score in Total **A**, EDI **B**, IDI **C**, and CDI **D** by Region. *FOHI* Fukuoka One Health Index, *EDI* External Drivers Index, *IDI* Internal Drivers Index, *CDI* Core Drivers Index

Figure 3 shows the top-20 municipalities in FOHI Score in total, EDI, IDI, and CDI. Total average scores for Fukuoka prefecture were 52.27 (Fig. 3A), 50.43 in EDI (Fig. 3B), 59.17 in IDI (Fig. 3C), 47.11 in CDI (Fig. 3D). Miyama had the highest score in total (63.71), in CDI (71.89) and ranked 16th in IDI (63.03). Izuka had the second highest score in total (60.21), ranked 8th in IDI (69.45) and 4th in CDI (57.44). Omuta ranked third in total (60.03), 1st in IDI (78.07), and 17th in CDI (49.20).

**Fig. 4:**
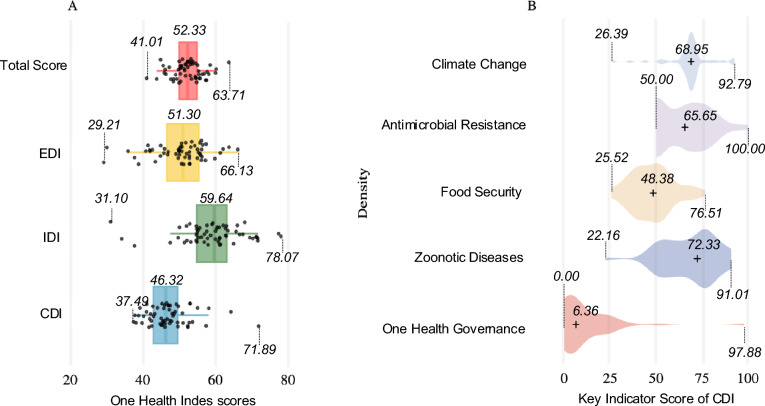
Density Plots Distribution of FOHI Components (**A**) and CDI Key Indicators (**B**). *FOHI* Fukuoka One Health Index, *EDI* External Drivers Index, *IDI* Internal Drivers Index, *CDI* Core Drivers Index

Figure 4A shows that the FOHI score for the total, EDI, IDI, and CDI vary considerably. Their median scores (median: lowest–highest scores) are as follows: Total score (52.33; 41.01–63.71), EDI score (51.03; 29.21–66.13), IDI score (59.64; 31.10–78.07), and CDI score (46.32; 37.49–71.89). IDI score has the highest median, while CDI score has the lowest median. Within the GOHI framework, the CDI has five dimensions; Climate change, AMR, Food security, Zoonotic Diseases, and One Health governance (Fig. 4B). Figure 4B shows that the median score was the highest in Zoonotic Diseases (72.33), followed by Climate change (68.95), AMR (65.65), Food security (48.38) and One Health governance (6.36). One Health governance had the lowest median score, the longest range, and was highly skewed to the right. AMR and Food security were slightly skewed to the right. These patterns suggest that fewer municipalities with higher scores are observed in the One Health governance, AMR, and food security. In contrast, Zoonotic Diseases was skewed to the left, indicating that fewer municipalities with lower scores are observed. For the Climate Change, most municipalities clustered around median, with other municipalities scattered in both lower and higher score ranges.

### Heat map and latent class analysis of municipal one health profiles

**Fig. 5:**
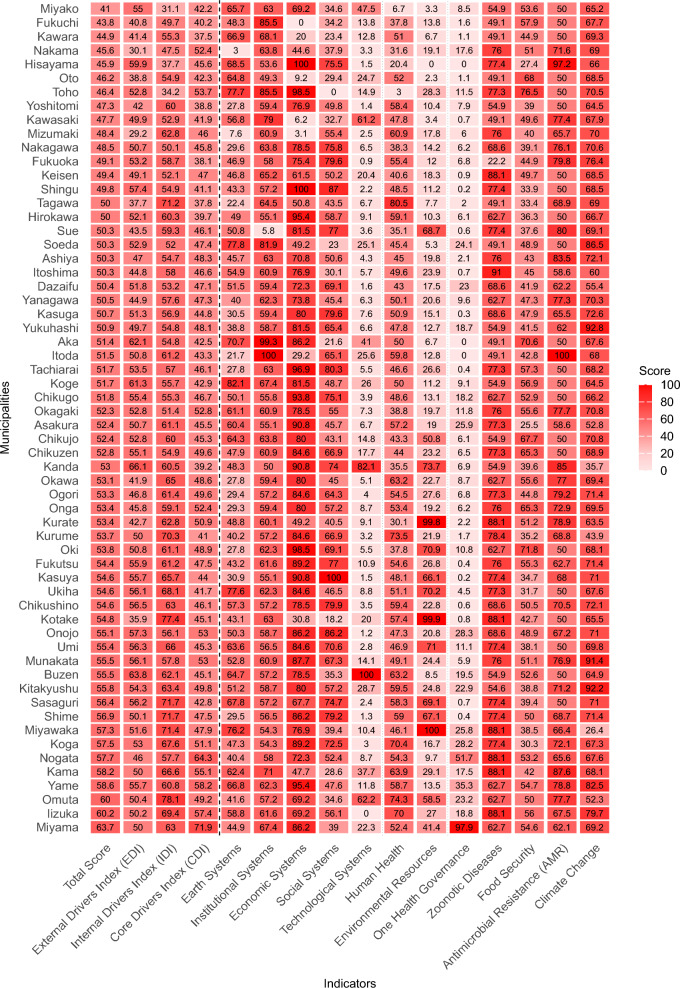
Comprehensive heat map of One Health performance across Fukuoka’s 60 municipalities. The B2. Animal Health and Ecosystem Diversity was omitted from the Fig. 5 since all municipalities had full score of 100

Figure 5 presents a comprehensive heat map visualizing the performance of all 60 municipalities across the multi-level FOHI framework. This visualization simultaneously displays both Level-1 (three index categories: EDI, IDI, CDI) and Level-2 (13 key indices) scores, enabling direct comparison of performance patterns across municipalities. The color gradient from light red (lower scores) to dark red (higher scores) illustrates the variation in implementation across different aspects of One Health.

The heat map reveals several notable patterns. First, there is considerable heterogeneity in municipal performance across different indices, with some municipalities demonstrating consistently high scores across multiple categories (e.g., Miyama, Iizuka, Omuta), while others show more variable performance. Second, the One Health Governance index (under CDI) displays the most uniformly low scores (lighter colors) across most municipalities, reinforcing our earlier finding that governance represents a critical gap in the region’s One Health implementation. Third, vertical patterns in the heat map highlight indices where performance is generally strong across municipalities (e.g., Animal Health and Ecosystem Diversity under IDI) or weak (e.g., One Health Governance).

**Fig. 6:**
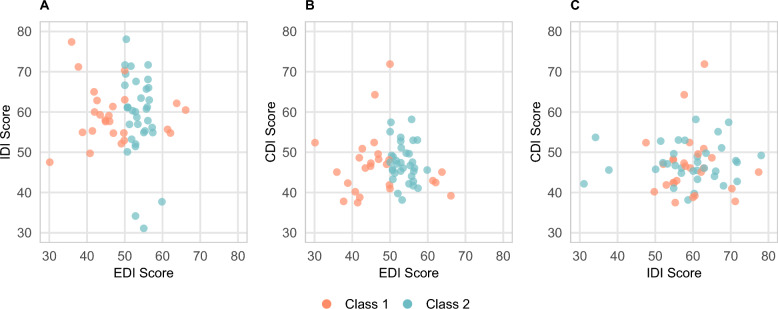
Latent Class Analysis based on EDI, IDI, CDI Scores across Fukuoka Municipalities. *EDI* External Drivers Index, *IDI* Internal Drivers Index, *CDI* Core Drivers Index

Figure 6A, B, and C show that Latent Class Analysis (LCA) identified two distinct classes across the 60 municipalities in Fukuoka Prefecture, primarily differentiated by their External Drivers Index (EDI) scores. The distribution pattern analysis reveals that Class 1 (Orange color) includes 26 municipalities and is characterized by a wider range of EDI scores, clustering predominantly below 50 with some municipalities scoring 60 or higher. In contrast, Class 2 (Blue color) consists of 34 municipalities with EDI scores concentrated between 50 and 60. These clustering patterns suggest the formation of two distinct municipal profiles: one with “typical” EDI scores (mostly between 50 and 60) and another with "non-typical" EDI scores (either below 50 or above 60).

## Discussion

This study demonstrates the feasibility and utility of adapting the GOHI to municipal-level contexts by developing the FOHI. As the first known attempt to localize GOHI for subnational administrative units, the FOHI offers a comprehensive framework to assess One Health implementation across 60 municipalities in Fukuoka Prefecture. The results provide critical insights into local capacities, gaps, and governance dynamics that are essential for operationalizing One Health strategies at the community level—where most health interventions ultimately unfold.

Our findings reveal substantial variation in One Health implementation across Fukuoka’s 60 municipalities, with a mean total score of 52.27 (range: 41.01–63.71). The highest performance was observed in the IDI with an average score of 59.17, suggesting relatively strong health system infrastructure and service resources compared to EDI (50.43) and CDI (47.11). The particularly low average score in the CDI suggests that while many municipalities have developed foundational infrastructure and service capacity, the translation of these capabilities into core One Health activities—specifically governance structures and food security measures—requires significant strengthening across most municipalities. This pattern aligns with findings from Zhang et al. [63], who identified similar gaps between policy infrastructure and operational implementation outcomes in their systematic analysis of One Health. In practical terms, our findings suggest that strong health infrastructure alone is insufficient for effective One Health implementation without accompanying governance and cross-sectoral coordination. As Zhang et al. [63] emphasized, bridging the gap between capacity and action requires focused policy efforts at the local level. In Fukuoka, FOHI results can guide targeted interventions—such as strengthening governance, enhancing intersectoral coordination, and improving accountability—to translate existing capacities into sustained One Health outcomes.

The lowest performance area, One Health governance (median score: 6.36), serves as a critical bottleneck for overall implementation, supported by other previous studies [64, 65] stating that integrated governance frameworks are fundamental enablers for effective One Health collaboration. Within the CDI, the contrast between the high performance in zoonotic disease management (median score: 72.33) and the poor performance in local governance structures reflects Japan’s strong public health surveillance system and Fukuoka Prefecture’s early adoption of integrated disease monitoring (e.g., monitoring wild animal and livestock meat processing, livestock’s AMR and waste, wild animals, birds, mosquitos, water, and air pollution), but reveals that technical capacity has developed faster than institutional arrangements needed to sustain multi-sectoral collaboration. In fact, the One Health multi-sectoral collaborations among the Health and Medical Care Department, the Citizen Life Department, Environment Department, and Agriculture, Forestry, and Fisheries Department have started in Fukuoka after FOHPAP was enacted in 2022. Fukuoka’s One Health governance is still in an early stage and in progress. This phenomenon, which Manlove et al. [66] termed “disciplinary siloing” highlights the persistent challenges of translating sectoral expertise into truly integrated approaches.

The variation in municipal performance across different One Health dimensions has important implications for policy and resource allocation. Municipalities with high IDI scores but low CDI scores, such as Omuta (ranked 1st in IDI but 17th in CDI), represent settings where existing healthcare infrastructure could be more effectively leveraged for One Health implementation through targeted governance interventions. Conversely, municipalities with more balanced performance across all indices, such as Miyama (ranked 1st overall and in CDI, 16th in IDI), may offer valuable models for integrated implementation that could inform capacity-building efforts in other regions. This difference could be partially due to the fact that Miyama not only declared as One Health promotion city in September 2021 as a first Japan’s municipality but also passed the city level One Health policy agreement in 2021 [67], while Omuta has declared as One Health promotion city later in January 2023 [67]. Nonetheless, the notable success of Miyama across multiple indices suggests it has developed effective mechanisms for cross-sectoral collaboration that could serve as a blueprint for other municipalities.

The latent class analysis identified two distinct municipal profiles, primarily differentiated by their EDI scores. Class 1 municipalities (*n* = 26) generally had lower EDI scores (below 50), while Class 2 municipalities (*n* = 34) clustered between EDI scores of 50–60. These two profiles demonstrated clear geographic clustering, as revealed by the spatial distribution analysis. Class 1 municipalities were predominantly located in inland and rural areas, often characterized by limited economic activity, lower population density, and aging infrastructure. In contrast, Class 2 municipalities were more commonly found in coastal and urbanized regions, with relatively stronger environmental, better transportation, and socio-economic conditions. Importantly, despite differences in structural conditions, considerable variability in CDI and IDI scores within each class indicates that local implementation outcomes and service capacities remain modifiable. This suggests that even municipalities with relatively weaker external conditions can strengthen their One Health performance through targeted governance and system-level interventions. In addition, this geographic patterning highlights the potential for regional coordination and inter-municipal knowledge exchange to enhance One Health implementation across diverse local contexts.

The FOHI framework’s adaptation process revealed several key challenges in localizing global assessment tools. First, data imputation was necessary for several indicators where municipal-level data were missing, which may affect score accuracy in specific cases. In particular, for two indicators (A1.2 and C5.3), over 70% of municipalities lacked direct measurements and required imputation using the mean values from a limited subset of municipalities. While this approach ensured completeness for index calculation, it may have obscured actual regional variation and introduced potential bias, limiting the interpretive robustness of these specific indicators. Second, the FOHI integrates data from a range of years (2020–2024), using 2022 as the intended baseline. This temporal inconsistency reflects data availability constraints but introduces the assumption that indicators from different years are reasonably comparable and representative of conditions in 2022. However, given the evolving public health and policy landscape—especially during the COVID-19 pandemic—this assumption may not always hold. These limitations highlight the need for caution in interpreting the results and underscore the importance of improving data availability and temporal alignment in future applications. Third, the weighting methodology, while systematic, relies on expert judgment that may not fully capture community priorities or stakeholder perspectives beyond the expert panel. Finally, and most importantly, direct transposition of GOHI to municipal levels was not always feasible due to differences in data availability, institutional structures, and policy priorities at the local level. Consequently, 14 of the original GOHI indicators were newly modified or entirely replaced to ensure contextual relevance and data compatibility with Fukuoka’s municipal systems. The newly developed indicators addressed specific local activities such as municipal-level One Health declarations, local business certification programs, education initiatives at elementary and secondary schools, disaster preparedness for pets, and surveillance systems specific to Fukuoka’s livestock and wildlife management practices. Similarly, indicators on Good Agricultural Practices (GAP) and local food promotion were adopted in place of GOHI’s broader food security metrics to capture grassroots-level policy engagement. Despite these limitations, the GOHI provides a valuable framework for tracking future progress in One Health implementation across Fukuoka Prefecture.

While our study utilized geographic visualization to display the distribution of FOHI scores across municipalities, future research could benefit from extending spatial visualization to include LCA-derived classes and employing formal spatial statistical methods. Such approaches would allow for the identification of significant spatial clusters, outliers, and spatial dependencies in both performance scores and implementation patterns, potentially revealing geographic determinants that influence One Health performance. These spatial analytical techniques could provide additional insights into how geographic proximity, shared resources, or regional policies might influence One Health implementation across neighboring municipalities, thereby enhancing the targeting of interventions and resource allocation.

Further research should focus on three priority areas: (1) longitudinal monitoring to assess changes in municipal performance over time; (2) qualitative case studies of high-performing municipalities to identify transferable implementation strategies; and (3) economic analyses using a cost–benefit approach to evaluate municipal interventions aimed at improving specific One Health indices. In parallel, local policymakers can use FOHI results not only to recognize performance gaps but also to design targeted interventions—such as investing in governance capacity where CDI scores are low, or initiating inter-municipal mentorship programs where high-performing municipalities support those with lower scores. Additionally, FOHI findings can inform the allocation of prefectural funding and technical assistance to ensure more equitable and effective implementation across diverse municipalities.

## Conclusions

This study demonstrates that adapting the GOHI framework to municipal settings is feasible and produces meaningful insights into local One Health implementation patterns. The FOHI’s systematic approach to evaluating municipal capacity provides a foundation for targeted policy interventions and resource allocation to strengthen community-level resilience against emerging health threats. The methodology developed here offers a blueprint for similar adaptations in other regions, potentially accelerating the operationalization of One Health principles in local governance contexts worldwide. Furthermore, the clear variation in performance across different One Health dimensions highlights the importance of tailored capacity-building approaches that address specific gaps in municipal preparedness for tackling health threats while building on existing strengths in healthcare infrastructure and public health surveillance.

## Supplementary Information

Supplementary Material 1. Supplemental tables ranking summary and raw data.

Supplementary Material 2. Supplemental material Fuzzy Analytic Hierarchy Process (FAHP).

Supplementary Material 3.Supplemental material Latent Class Analysis (LCA).

Supplementary Material 4. Technical File for Fukuoka One Health Indicators Detailed Information.

Supplementary Material 5. Questionnaire for weighting.

Supplementary Material 6. List of GOHI Sub-Indicators.

## Data Availability

The data from the study are deposited at Institute for Asian and Oceanian Studies, Kyushu University, Fukuoka, Japan. The data can be accessible based on personal communication to corresponding author (yokota.fumihiko.785@m.kyushu-u.ac.jp).

## Abbreviations

AMR: Antimicrobial Resistance
BIC: Bayesian Information Criterion
CDI: Core Drivers Index
EDI: External Drivers Index
FAHP: Fuzzy Analytic Hierarchy Process
FOHI: Fukuoka One Health Index
FOHPAP: Fukuoka One Health Promotion Action Plan
GAP: Good Agricultural Practices
GDP: Gross Domestic Product
GOHI: Global One Health Index
IQR: Interquartile Range
IDI: Internal Drivers Index
JANIS: Japan Nosocomial Infections Surveillance
LCA: Latent Class Analysis
OH: One Health
*SD*: Standard Deviation
WHO: World Health Organization
WOAH: World Organization for Animal Health
WBGT: Wet Bulb Globe Temperature
