# Supplementary material for: Adaptation of global One Health evaluation framework to municipal levels in Fukuoka, Japan
Source: Infect Dis Poverty. 2025 Nov 13;14:116. doi: 10.1186/s40249-025-01380-y (PMC12613462; doi:10.1186/s40249-025-01380-y)
Supplement: Supplementary file 1 — Supplementary Material 1. Supplemental tables ranking summary and raw data. [file 40249_2025_1380_MOESM1_ESM.docx]

| Supplement Table 1A. Calculated score ranking of 3 categories by municipality (n=60) | | | | | | | | | | | |
| --- | --- | --- | --- | --- | --- | --- | --- | --- | --- | --- | --- |
| **#** | **Municipality** | **1.Total Score** | **#** | **Municipality** | **2.External Drivers Index(EDI)** | **#** | **Municipality** | **3.Internal Drivers Index(IDI)** | **#** | **Municipality** | **4.Core Drivers Index(CDI)** |
| 1 | Miyama | 63.71198 | 1 | Kanda | 66.13288 | 1 | Omuta | 78.06524 | 1 | Miyama | 71.88646 |
| 2 | Iizuka | 60.20987 | 2 | Buzen | 63.75837 | 2 | Kotake | 77.39229 | 2 | Nogata | 64.28366 |
| 3 | Omuta | 60.03016 | 3 | Aka | 62.09849 | 3 | Sasaguri | 71.69256 | 3 | Yame | 58.15899 |
| 4 | Yame | 58.55799 | 4 | Koge | 61.34816 | 4 | Shime | 71.66751 | 4 | Iizuka | 57.43704 |
| 5 | Kama | 58.17634 | 5 | Hisayama | 59.8515 | 5 | Miyawaka | 71.37971 | 5 | Kama | 55.10254 |
| 6 | Nogata | 57.74259 | 6 | Shingu | 57.41997 | 6 | Tagawa | 71.19093 | 6 | Toho | 53.69386 |
| 7 | Koga | 57.54677 | 7 | Onojo | 57.25249 | 7 | Kurume | 70.29821 | 7 | Onojo | 53.03167 |
| 8 | Miyawaka | 57.33308 | 8 | Chikushino | 56.47595 | 8 | Iizuka | 69.44781 | 8 | Munakata | 52.97184 |
| 9 | Shime | 56.91657 | 9 | Umi | 56.29684 | 9 | Ukiha | 68.07758 | 9 | Okagaki | 52.76669 |
| 10 | Sasaguri | 56.366 | 10 | Sasaguri | 56.1804 | 10 | Koga | 67.57909 | 10 | Onga | 52.41686 |
| 11 | Kitakyushu | 55.78775 | 11 | Ukiha | 56.14135 | 11 | Kama | 66.63782 | 11 | Nakama | 52.38326 |
| 12 | Buzen | 55.5266 | 12 | Munakata | 56.12078 | 12 | Umi | 66.01513 | 12 | Koga | 51.09044 |
| 13 | Munakata | 55.46037 | 13 | Fukutsu | 55.9465 | 13 | Kasuya | 65.69332 | 13 | Kurate | 50.88559 |
| 14 | Umi | 55.35007 | 14 | Kasuya | 55.72462 | 14 | Okawa | 65.00161 | 14 | Kitakyushu | 49.76668 |
| 15 | Onojo | 55.11851 | 15 | Yame | 55.71574 | 15 | Kitakyushu | 63.43521 | 15 | Chikuzen | 49.62897 |
| 16 | Kotake | 54.82801 | 16 | Chikugo | 55.35345 | 16 | Miyama | 63.02549 | 16 | Ogori | 49.56533 |
| 17 | Chikushino | 54.59421 | 17 | Chikuzen | 55.05115 | 17 | Chikushino | 62.9725 | 17 | Omuta | 49.2019 |
| 18 | Ukiha | 54.58895 | 18 | Miyako | 55.02971 | 18 | Kurate | 62.84915 | 18 | Oki | 48.88577 |
| 19 | Kasuya | 54.58875 | 19 | Kitakyushu | 54.29896 | 19 | Mizumaki | 62.79666 | 19 | Okawa | 48.61635 |
| 20 | Fukutsu | 54.43506 | 20 | Tachiarai | 53.46155 | 20 | Buzen | 62.14884 | 20 | Ashiya | 48.26977 |
| 21 | Oki | 53.80053 | 21 | Fukuoka | 53.19643 | 21 | Ogori | 61.35368 | 21 | Yukuhashi | 48.08433 |
| 22 | Kurume | 53.72634 | 22 | Koga | 52.97583 | 22 | Fukutsu | 61.2241 | 22 | Miyawaka | 47.91204 |
| 23 | Kurate | 53.40709 | 23 | Soeda | 52.86149 | 23 | Itoda | 61.17895 | 23 | Fukutsu | 47.52883 |
| 24 | Onga | 53.35721 | 24 | Toho | 52.83527 | 24 | Oki | 61.14006 | 24 | Shime | 47.50335 |
| 25 | Ogori | 53.26428 | 25 | Okagaki | 52.82681 | 25 | Asakura | 61.06549 | 25 | Soeda | 47.40055 |
| 26 | Okawa | 53.08806 | 26 | Chikujo | 52.82098 | 26 | Yame | 60.75456 | 26 | Yanagawa | 47.29003 |
| 27 | Kanda | 53.04842 | 27 | Hirokawa | 52.07396 | 27 | Kanda | 60.4808 | 27 | Dazaifu | 47.10514 |
| 28 | Chikuzen | 52.77457 | 28 | Dazaifu | 51.81607 | 28 | Hirokawa | 60.32257 | 28 | Keisen | 47.03233 |
| 29 | Chikujo | 52.39711 | 29 | Miyawaka | 51.62518 | 29 | Chikujo | 60.03661 | 29 | Chikugo | 46.70227 |
| 30 | Asakura | 52.36047 | 30 | Kasuga | 51.25342 | 30 | Yoshitomi | 60.00099 | 30 | Itoshima | 46.55076 |
| 31 | Okagaki | 52.29462 | 31 | Oki | 50.80385 | 31 | Sue | 59.27514 | 31 | Tachiarai | 46.08347 |
| 32 | Chikugo | 51.79426 | 32 | Itoda | 50.75724 | 32 | Onga | 59.07102 | 32 | Sue | 46.07767 |
| 33 | Koge | 51.7461 | 33 | Nakagawa | 50.69294 | 33 | Fukuoka | 58.69009 | 33 | Chikushino | 46.0622 |
| 34 | Tachiarai | 51.72511 | 34 | Asakura | 50.68174 | 34 | Itoshima | 57.98288 | 34 | Mizumaki | 46.0379 |
| 35 | Itoda | 51.51111 | 35 | Omuta | 50.4005 | 35 | Munakata | 57.83534 | 35 | Nakagawa | 45.76279 |
| 36 | Aka | 51.39214 | 36 | Iizuka | 50.24346 | 36 | Nogata | 57.6719 | 36 | Hisayama | 45.58917 |
| 37 | Yukuhashi | 50.91404 | 37 | Shime | 50.05093 | 37 | Yanagawa | 57.62287 | 37 | Asakura | 45.49796 |
| 38 | Kasuga | 50.69736 | 38 | Kama | 50.01684 | 38 | Tachiarai | 56.96197 | 38 | Chikujo | 45.3293 |
| 39 | Yanagawa | 50.53611 | 39 | Miyama | 49.9906 | 39 | Kasuga | 56.924 | 39 | Umi | 45.2875 |
| 40 | Dazaifu | 50.40475 | 40 | Kurume | 49.97304 | 40 | Onojo | 56.13689 | 40 | Buzen | 45.06674 |
| 41 | Itoshima | 50.348 | 41 | Kawasaki | 49.85349 | 41 | Koge | 55.67103 | 41 | Kotake | 45.06538 |
| 42 | Ashiya | 50.34405 | 42 | Yukuhashi | 49.74977 | 42 | Chikugo | 55.29406 | 42 | Kasuga | 44.82067 |
| 43 | Soeda | 50.32498 | 43 | Keisen | 49.10091 | 43 | Kawara | 55.28747 | 43 | Kasuya | 44.02879 |
| 44 | Sue | 50.31508 | 44 | Ashiya | 46.97836 | 44 | Oto | 54.94219 | 44 | Itoda | 43.27768 |
| 45 | Hirokawa | 50.04864 | 45 | Ogori | 46.84353 | 45 | Chikuzen | 54.88863 | 45 | Koge | 42.94424 |
| 46 | Tagawa | 49.99563 | 46 | Nogata | 45.98588 | 46 | Shingu | 54.88081 | 46 | Sasaguri | 42.7572 |
| 47 | Shingu | 49.80375 | 47 | Onga | 45.78684 | 47 | Yukuhashi | 54.79425 | 47 | Aka | 42.47481 |
| 48 | Keisen | 49.36285 | 48 | Yanagawa | 44.92129 | 48 | Aka | 54.766 | 48 | Oto | 42.34336 |
| 49 | Fukuoka | 49.05169 | 49 | Itoshima | 44.84357 | 49 | Ashiya | 54.73568 | 49 | Miyako | 42.15767 |
| 50 | Nakagawa | 48.45685 | 50 | Sue | 43.45793 | 50 | Dazaifu | 53.22389 | 50 | Kawasaki | 41.88091 |
| 51 | Mizumaki | 48.3776 | 51 | Kurate | 42.65315 | 51 | Kawasaki | 52.93137 | 51 | Ukiha | 41.66673 |
| 52 | Kawasaki | 47.71939 | 52 | Yoshitomi | 41.98454 | 52 | Keisen | 52.12929 | 52 | Shingu | 41.06536 |
| 53 | Yoshitomi | 47.27445 | 53 | Okawa | 41.86081 | 53 | Soeda | 52.03172 | 53 | Kurume | 40.96925 |
| 54 | Toho | 46.35956 | 54 | Kawara | 41.41575 | 54 | Okagaki | 51.43914 | 54 | Fukuchi | 40.18605 |
| 55 | Oto | 46.15599 | 55 | Fukuchi | 40.79467 | 55 | Nakagawa | 50.09112 | 55 | Hirokawa | 39.74169 |
| 56 | Hisayama | 45.91754 | 56 | Oto | 38.81017 | 56 | Fukuchi | 49.72919 | 56 | Kanda | 39.19004 |
| 57 | Nakama | 45.591 | 57 | Tagawa | 37.71372 | 57 | Nakama | 47.52807 | 57 | Yoshitomi | 38.80405 |
| 58 | Kawara | 44.8855 | 58 | Kotake | 35.91068 | 58 | Hisayama | 37.70253 | 58 | Fukuoka | 38.14551 |
| 59 | Fukuchi | 43.81405 | 59 | Nakama | 30.11858 | 59 | Toho | 34.17264 | 59 | Tagawa | 37.80164 |
| 60 | Miyako | 41.0139 | 60 | Mizumaki | 29.21299 | 60 | Miyako | 31.1029 | 60 | Kawara | 37.49174 |

| Supplement Table 1B: Calculated score ranking of 13 key indicators by municipality (#1-#4) | | | | | | | | | | | |
| --- | --- | --- | --- | --- | --- | --- | --- | --- | --- | --- | --- |
| **#** | **Municipality** | **1.Earth Systems** | **#** | **Municipality** | **2.Institutional Systems** | **#** | **Municipality** | **3.Economic Systems** | **#** | **Municipality** | **4.Social Systems** |
| 1 | Koge | 82.08919 | 1 | Itoda | 100 | 1 | Hisayama | 100 | 1 | Kasuya | 100 |
| 2 | Soeda | 77.8125 | 2 | Aka | 99.27536 | 2 | Shingu | 100 | 2 | Shingu | 86.98885 |
| 3 | Toho | 77.67491 | 3 | Toho | 85.50725 | 3 | Oki | 98.46154 | 3 | Onojo | 86.24535 |
| 4 | Ukiha | 77.63198 | 4 | Fukuchi | 85.50725 | 4 | Toho | 98.46154 | 4 | Tachiarai | 80.2974 |
| 5 | Miyawaka | 76.19282 | 5 | Soeda | 81.88406 | 5 | Tachiarai | 96.92308 | 5 | Chikushino | 79.92565 |
| 6 | Aka | 70.74494 | 6 | Kawasaki | 78.98551 | 6 | Yame | 95.38461 | 6 | Kasuga | 79.5539 |
| 7 | Hisayama | 68.53376 | 7 | Kama | 71.01449 | 7 | Hirokawa | 95.38461 | 7 | Fukuoka | 79.5539 |
| 8 | Sasaguri | 67.81856 | 8 | Kawara | 68.11594 | 8 | Chikugo | 93.84615 | 8 | Shime | 79.18216 |
| 9 | Kawara | 66.91591 | 9 | Miyama | 67.3913 | 9 | Asakura | 90.76923 | 9 | Fukutsu | 76.95167 |
| 10 | Yame | 66.80142 | 10 | Koge | 67.3913 | 10 | Kasuya | 90.76923 | 10 | Sue | 76.95167 |
| 11 | Miyako | 65.74124 | 11 | Keisen | 65.21739 | 11 | Kanda | 90.76923 | 11 | Nakagawa | 75.83643 |
| 12 | Oto | 64.75286 | 12 | Tagawa | 64.49275 | 12 | Fukutsu | 89.23077 | 12 | Hisayama | 75.46468 |
| 13 | Buzen | 64.71295 | 13 | Nakagawa | 63.76812 | 13 | Koga | 89.23077 | 13 | Chikugo | 75.09294 |
| 14 | Chikujo | 64.30763 | 14 | Nakama | 63.76812 | 14 | Munakata | 87.69231 | 14 | Sasaguri | 74.72119 |
| 15 | Umi | 63.57798 | 15 | Chikujo | 63.76812 | 15 | Miyama | 86.15385 | 15 | Kanda | 73.97769 |
| 16 | Kama | 62.36216 | 16 | Miyako | 63.04348 | 16 | Aka | 86.15385 | 16 | Koga | 72.49071 |
| 17 | Okagaki | 61.14534 | 17 | Tachiarai | 63.04348 | 17 | Onojo | 86.15385 | 17 | Umi | 70.63197 |
| 18 | Asakura | 60.43157 | 18 | Ashiya | 63.04348 | 18 | Shime | 86.15385 | 18 | Oki | 69.14498 |
| 19 | Iizuka | 58.78418 | 19 | Kotake | 63.04348 | 19 | Ukiha | 84.61539 | 19 | Dazaifu | 69.14498 |
| 20 | Chikushino | 57.29584 | 20 | Ukiha | 62.31884 | 20 | Kurume | 84.61539 | 20 | Munakata | 67.28625 |
| 21 | Kawasaki | 56.78444 | 21 | Yame | 62.31884 | 21 | Ogori | 84.61539 | 21 | Kurume | 66.9145 |
| 22 | Itoshima | 54.90622 | 22 | Oki | 62.31884 | 22 | Umi | 84.61539 | 22 | Chikuzen | 66.9145 |
| 23 | Munakata | 52.75748 | 23 | Yanagawa | 62.31884 | 23 | Chikuzen | 84.61539 | 23 | Yukuhashi | 65.42751 |
| 24 | Dazaifu | 51.52906 | 24 | Iizuka | 61.5942 | 24 | Yukuhashi | 81.53846 | 24 | Itoda | 65.05576 |
| 25 | Kitakyushu | 51.20557 | 25 | Fukutsu | 61.5942 | 25 | Koge | 81.53846 | 25 | Ogori | 64.31227 |
| 26 | Sue | 50.76076 | 26 | Okagaki | 60.86957 | 26 | Sue | 81.53846 | 26 | Hirokawa | 58.73606 |
| 27 | Onojo | 50.33128 | 27 | Itoshima | 60.86957 | 27 | Kitakyushu | 80 | 27 | Kitakyushu | 57.24907 |
| 28 | Chikugo | 50.10002 | 28 | Mizumaki | 60.86957 | 28 | Kasuga | 80 | 28 | Onga | 57.24907 |
| 29 | Hirokawa | 49.02192 | 29 | Chikuzen | 60.86957 | 29 | Okawa | 80 | 29 | Iizuka | 56.13383 |
| 30 | Kurate | 48.83981 | 30 | Munakata | 60.86957 | 30 | Onga | 80 | 30 | Mizumaki | 55.39033 |
| 31 | Kanda | 48.29269 | 31 | Kurate | 60.14493 | 31 | Chikujo | 80 | 31 | Okagaki | 55.01859 |
| 32 | Fukuchi | 48.29032 | 32 | Kasuga | 59.42029 | 32 | Okagaki | 78.46154 | 32 | Nogata | 52.41636 |
| 33 | Chikuzen | 47.89164 | 33 | Okawa | 59.42029 | 33 | Buzen | 78.46154 | 33 | Ashiya | 50.55762 |
| 34 | Koga | 47.3371 | 34 | Yoshitomi | 59.42029 | 34 | Nakagawa | 78.46154 | 34 | Keisen | 50.18587 |
| 35 | Fukuoka | 46.92226 | 35 | Dazaifu | 59.42029 | 35 | Chikushino | 78.46154 | 35 | Yoshitomi | 49.81413 |
| 36 | Keisen | 46.78871 | 36 | Onga | 59.42029 | 36 | Miyawaka | 76.92308 | 36 | Koge | 48.69888 |
| 37 | Ashiya | 45.74041 | 37 | Kitakyushu | 58.69565 | 37 | Yoshitomi | 76.92308 | 37 | Yame | 47.58364 |
| 38 | Miyama | 44.85376 | 38 | Onojo | 58.69565 | 38 | Itoshima | 76.92308 | 38 | Ukiha | 46.4684 |
| 39 | Shingu | 43.32746 | 39 | Yukuhashi | 58.69565 | 39 | Fukuoka | 75.38462 | 39 | Asakura | 45.72491 |
| 40 | Fukutsu | 43.17745 | 40 | Fukuoka | 57.97101 | 40 | Yanagawa | 73.84615 | 40 | Yanagawa | 45.35316 |
| 41 | Kotake | 43.07061 | 41 | Nogata | 57.97101 | 41 | Dazaifu | 72.30769 | 41 | Okawa | 44.98141 |
| 42 | Omuta | 41.60569 | 42 | Omuta | 57.24638 | 42 | Nogata | 72.30769 | 42 | Tagawa | 43.49442 |
| 43 | Nogata | 40.41375 | 43 | Kurume | 57.24638 | 43 | Ashiya | 70.76923 | 43 | Chikujo | 43.12268 |
| 44 | Kurume | 40.24112 | 44 | Buzen | 57.24638 | 44 | Miyako | 69.23077 | 44 | Kurate | 40.52044 |
| 45 | Yanagawa | 40 | 45 | Ogori | 57.24638 | 45 | Omuta | 69.23077 | 45 | Miyawaka | 39.4052 |
| 46 | Yukuhashi | 38.81249 | 46 | Sasaguri | 57.24638 | 46 | Iizuka | 69.23077 | 46 | Miyama | 39.03346 |
| 47 | Kasuya | 30.85427 | 47 | Shingu | 57.24638 | 47 | Sasaguri | 67.69231 | 47 | Nakama | 37.91822 |
| 48 | Kasuga | 30.47029 | 48 | Chikushino | 57.24638 | 48 | Keisen | 61.53846 | 48 | Buzen | 35.31598 |
| 49 | Nakagawa | 29.60358 | 49 | Umi | 56.52174 | 49 | Tagawa | 50.76923 | 49 | Miyako | 34.57249 |
| 50 | Shime | 29.52984 | 50 | Shime | 56.52174 | 50 | Kurate | 49.23077 | 50 | Omuta | 34.57249 |
| 51 | Ogori | 29.38652 | 51 | Chikugo | 55.7971 | 51 | Soeda | 49.23077 | 51 | Fukuchi | 34.20074 |
| 52 | Onga | 29.34658 | 52 | Asakura | 55.07246 | 52 | Kama | 47.69231 | 52 | Kawasaki | 32.71375 |
| 53 | Tachiarai | 27.83863 | 53 | Hirokawa | 55.07246 | 53 | Nakama | 44.61539 | 53 | Itoshima | 30.11152 |
| 54 | Okawa | 27.8125 | 54 | Kasuya | 55.07246 | 54 | Kotake | 30.76924 | 54 | Oto | 29.36803 |
| 55 | Oki | 27.8125 | 55 | Miyawaka | 54.34783 | 55 | Itoda | 29.23078 | 55 | Kama | 28.62453 |
| 56 | Yoshitomi | 27.8125 | 56 | Koga | 54.34783 | 56 | Kawara | 20 | 56 | Kawara | 23.42007 |
| 57 | Tagawa | 22.4324 | 57 | Hisayama | 53.62319 | 57 | Oto | 9.23077 | 57 | Soeda | 23.04833 |
| 58 | Itoda | 21.70562 | 58 | Kanda | 50 | 58 | Kawasaki | 6.15385 | 58 | Aka | 21.56134 |
| 59 | Mizumaki | 7.62217 | 59 | Oto | 49.27536 | 59 | Mizumaki | 3.07693 | 59 | Kotake | 18.21561 |
| 60 | Nakama | 3.02962 | 60 | Sue | 5.7971 | 60 | Fukuchi | 0 | 60 | Toho | 0 |

| Supplement Table 1B: Calculated score ranking of 13 key indicators by municipality (#5-#8) | | | | | | | | | | | |
| --- | --- | --- | --- | --- | --- | --- | --- | --- | --- | --- | --- |
| **#** | **Municipality** | **5.Technological Systems** | **#** | **Municipality** | **6.Human Health** | **#** | **Municipality** | **7.Animal Health and Ecosystem Diversity** | **#** | **Municipality** | **8.Environmental Resources** |
| 1 | Buzen | 100 | 1 | Tagawa | 80.54928 | 1 | Ukiha | 100 | 1 | Miyawaka | 100 |
| 2 | Kanda | 82.07329 | 2 | Omuta | 74.33645 | 2 | Miyako | 100 | 2 | Kotake | 99.89247 |
| 3 | Omuta | 62.18271 | 3 | Kurume | 73.50611 | 3 | Miyama | 100 | 3 | Kurate | 99.78495 |
| 4 | Kawasaki | 61.15094 | 4 | Koga | 70.37145 | 4 | Kurate | 100 | 4 | Kanda | 73.65591 |
| 5 | Miyako | 47.45678 | 5 | Iizuka | 69.99466 | 5 | Yame | 100 | 5 | Umi | 70.96774 |
| 6 | Aka | 40.99591 | 6 | Kama | 63.88656 | 6 | Kitakyushu | 100 | 6 | Oki | 70.86022 |
| 7 | Kama | 37.65845 | 7 | Okawa | 63.23953 | 7 | Asakura | 100 | 7 | Ukiha | 70.21506 |
| 8 | Kitakyushu | 28.72903 | 8 | Buzen | 63.22374 | 8 | Aka | 100 | 8 | Sasaguri | 69.13979 |
| 9 | Koge | 25.97848 | 9 | Mizumaki | 60.91148 | 9 | Kawasaki | 100 | 9 | Sue | 68.70968 |
| 10 | Itoda | 25.593 | 10 | Itoda | 59.77138 | 10 | Kasuga | 100 | 10 | Shime | 67.09678 |
| 11 | Soeda | 25.0915 | 11 | Kitakyushu | 59.47518 | 11 | Oto | 100 | 11 | Kasuya | 66.12903 |
| 12 | Oto | 24.65354 | 12 | Chikushino | 59.37475 | 12 | Okawa | 100 | 12 | Omuta | 58.49462 |
| 13 | Miyama | 22.34814 | 13 | Hirokawa | 59.09146 | 13 | Tachiarai | 100 | 13 | Chikujo | 50.75269 |
| 14 | Keisen | 20.37857 | 14 | Shime | 59.02999 | 14 | Omuta | 100 | 14 | Miyama | 41.39785 |
| 15 | Kotake | 20.03445 | 15 | Yame | 58.68727 | 15 | Oki | 100 | 15 | Kama | 29.13979 |
| 16 | Chikuzen | 17.71206 | 16 | Yoshitomi | 58.44477 | 16 | Onojo | 100 | 16 | Toho | 28.27957 |
| 17 | Toho | 14.9154 | 17 | Sasaguri | 58.3056 | 17 | Toho | 100 | 17 | Ogori | 27.63441 |
| 18 | Chikujo | 14.76857 | 18 | Kotake | 57.43279 | 18 | Iizuka | 100 | 18 | Iizuka | 26.98925 |
| 19 | Munakata | 14.11525 | 19 | Asakura | 57.20209 | 19 | Fukuoka | 100 | 19 | Fukutsu | 26.77419 |
| 20 | Fukuchi | 13.82789 | 20 | Fukuoka | 55.365 | 20 | Fukutsu | 100 | 20 | Tachiarai | 26.55914 |
| 21 | Kawara | 12.84885 | 21 | Fukutsu | 54.577 | 21 | Fukuchi | 100 | 21 | Kitakyushu | 24.83871 |
| 22 | Yame | 11.80422 | 22 | Ogori | 54.49631 | 22 | Okagaki | 100 | 22 | Munakata | 24.4086 |
| 23 | Fukutsu | 10.89782 | 23 | Nogata | 54.33951 | 23 | Miyawaka | 100 | 23 | Itoshima | 23.87097 |
| 24 | Miyawaka | 10.39943 | 24 | Onga | 53.36197 | 24 | Koga | 100 | 24 | Chikuzen | 23.22581 |
| 25 | Hirokawa | 9.12764 | 25 | Miyama | 52.44886 | 25 | Hirokawa | 100 | 25 | Chikushino | 22.7957 |
| 26 | Kurate | 9.11385 | 26 | Oto | 51.99713 | 26 | Keisen | 100 | 26 | Okawa | 22.68817 |
| 27 | Ukiha | 8.76283 | 27 | Ukiha | 51.08648 | 27 | Yukuhashi | 100 | 27 | Kurume | 21.93548 |
| 28 | Nogata | 8.743 | 28 | Kawara | 50.98279 | 28 | Yoshitomi | 100 | 28 | Onojo | 20.75269 |
| 29 | Onga | 8.69064 | 29 | Kasuga | 50.8994 | 29 | Kama | 100 | 29 | Yanagawa | 20.64516 |
| 30 | Kasuga | 7.61296 | 30 | Yanagawa | 50.10475 | 30 | Kurume | 100 | 30 | Ashiya | 19.78495 |
| 31 | Okagaki | 7.25399 | 31 | Aka | 50 | 31 | Hisayama | 100 | 31 | Okagaki | 19.67742 |
| 32 | Tagawa | 6.7386 | 32 | Koge | 50 | 32 | Buzen | 100 | 32 | Onga | 19.24731 |
| 33 | Asakura | 6.66673 | 33 | Itoshima | 49.56491 | 33 | Yanagawa | 100 | 33 | Nakama | 19.13978 |
| 34 | Yukuhashi | 6.61916 | 34 | Munakata | 49.08379 | 34 | Ashiya | 100 | 34 | Asakura | 19.03226 |
| 35 | Nakagawa | 6.46281 | 35 | Chikugo | 48.55854 | 35 | Itoshima | 100 | 35 | Keisen | 18.27957 |
| 36 | Yanagawa | 6.32578 | 36 | Shingu | 48.51071 | 36 | Itoda | 100 | 36 | Mizumaki | 17.84946 |
| 37 | Itoshima | 5.67549 | 37 | Kasuya | 48.13619 | 37 | Nakagawa | 100 | 37 | Dazaifu | 17.52688 |
| 38 | Tachiarai | 5.5417 | 38 | Yukuhashi | 47.779 | 38 | Kasuya | 100 | 38 | Koga | 16.66667 |
| 39 | Oki | 5.48554 | 39 | Kawasaki | 47.76068 | 39 | Koge | 100 | 39 | Kasuga | 15.05376 |
| 40 | Okawa | 5.13737 | 40 | Onojo | 47.26358 | 40 | Mizumaki | 100 | 40 | Nakagawa | 14.19355 |
| 41 | Ashiya | 4.30378 | 41 | Umi | 46.91519 | 41 | Dazaifu | 100 | 41 | Fukuchi | 13.76344 |
| 42 | Ogori | 3.97803 | 42 | Tachiarai | 46.62555 | 42 | Soeda | 100 | 42 | Yame | 13.54839 |
| 43 | Chikugo | 3.90745 | 43 | Miyawaka | 46.06051 | 43 | Tagawa | 100 | 43 | Chikugo | 13.11828 |
| 44 | Sue | 3.60133 | 44 | Soeda | 45.37476 | 44 | Kawara | 100 | 44 | Itoda | 12.7957 |
| 45 | Chikushino | 3.47539 | 45 | Ashiya | 44.98827 | 45 | Ogori | 100 | 45 | Yukuhashi | 12.68817 |
| 46 | Nakama | 3.29028 | 46 | Chikuzen | 43.97696 | 46 | Kotake | 100 | 46 | Fukuoka | 12.04301 |
| 47 | Kurume | 3.21241 | 47 | Chikujo | 43.28264 | 47 | Sasaguri | 100 | 47 | Koge | 11.1828 |
| 48 | Koga | 2.96491 | 48 | Dazaifu | 42.99189 | 48 | Shingu | 100 | 48 | Shingu | 11.1828 |
| 49 | Umi | 2.7843 | 49 | Keisen | 40.64468 | 49 | Sue | 100 | 49 | Yoshitomi | 10.43011 |
| 50 | Mizumaki | 2.53518 | 50 | Okagaki | 38.81603 | 50 | Kanda | 100 | 50 | Hirokawa | 10.32258 |
| 51 | Sasaguri | 2.44922 | 51 | Nakagawa | 38.34665 | 51 | Umi | 100 | 51 | Nogata | 9.67742 |
| 52 | Shingu | 2.22067 | 52 | Fukuchi | 37.82699 | 52 | Onga | 100 | 52 | Buzen | 8.49462 |
| 53 | Dazaifu | 1.55109 | 53 | Oki | 37.76795 | 53 | Nogata | 100 | 53 | Tagawa | 7.74194 |
| 54 | Hisayama | 1.5288 | 54 | Kanda | 35.46957 | 54 | Shime | 100 | 54 | Aka | 6.66667 |
| 55 | Kasuya | 1.50691 | 55 | Sue | 35.06544 | 55 | Nakama | 100 | 55 | Kawara | 6.66667 |
| 56 | Yoshitomi | 1.3596 | 56 | Nakama | 31.64805 | 56 | Chikugo | 100 | 56 | Soeda | 5.26882 |
| 57 | Shime | 1.26225 | 57 | Kurate | 30.06454 | 57 | Chikuzen | 100 | 57 | Kawasaki | 3.44086 |
| 58 | Onojo | 1.18014 | 58 | Hisayama | 20.35908 | 58 | Chikushino | 100 | 58 | Miyako | 3.33333 |
| 59 | Fukuoka | 0.93798 | 59 | Miyako | 6.66207 | 59 | Chikujo | 100 | 59 | Oto | 2.25806 |
| 60 | Iizuka | 0 | 60 | Toho | 3.02566 | 60 | Munakata | 100 | 60 | Hisayama | 0 |

| Supplement Table 1B. Calculated score ranking of 13 key indicators by municipality (#9-#12) | | | | | | | | | | | |
| --- | --- | --- | --- | --- | --- | --- | --- | --- | --- | --- | --- |
| **#** | **Municipality** | **9.One Health Governance** | **#** | **Municipality** | **10.Zoonotic Diseases** | **#** | **Municipality** | **11.Food Security** | **#** | **Municipality** | **12.Antimicrobial Resistance (AMR)** |
| 1 | Miyama | 97.88044 | 1 | Itoshima | 91.00582 | 1 | Toho | 76.51364 | 1 | Itoda | 100 |
| 2 | Nogata | 51.7058 | 2 | Kurate | 88.14282 | 2 | Oki | 71.77792 | 2 | Hisayama | 97.18737 |
| 3 | Yame | 35.26405 | 3 | Iizuka | 88.14282 | 3 | Aka | 70.6248 | 3 | Kama | 87.60345 |
| 4 | Onojo | 28.32467 | 4 | Miyawaka | 88.14282 | 4 | Oto | 68.01439 | 4 | Kanda | 84.98369 |
| 5 | Koga | 28.1539 | 5 | Keisen | 88.14282 | 5 | Chikujo | 67.74892 | 5 | Ashiya | 83.5229 |
| 6 | Asakura | 25.92148 | 6 | Kama | 88.14282 | 6 | Onga | 65.3457 | 6 | Sue | 79.97526 |
| 7 | Miyawaka | 25.7803 | 7 | Kotake | 88.14282 | 7 | Chikuzen | 65.32391 | 7 | Fukuoka | 79.76117 |
| 8 | Soeda | 24.11021 | 8 | Nogata | 88.14282 | 8 | Fukuchi | 57.85378 | 8 | Ogori | 79.19679 |
| 9 | Omuta | 23.18038 | 9 | Kurume | 78.41164 | 9 | Tachiarai | 57.34395 | 9 | Kurate | 78.91998 |
| 10 | Dazaifu | 23.0011 | 10 | Koga | 77.44908 | 10 | Koge | 56.85826 | 10 | Yame | 78.84825 |
| 11 | Kitakyushu | 22.89604 | 11 | Hisayama | 77.44908 | 11 | Iizuka | 55.95732 | 11 | Okagaki | 77.69348 |
| 12 | Buzen | 19.47634 | 12 | Kasuya | 77.44908 | 12 | Okawa | 55.63033 | 12 | Omuta | 77.69122 |
| 13 | Iizuka | 18.76388 | 13 | Sasaguri | 77.44908 | 13 | Okagaki | 55.56816 | 13 | Kawasaki | 77.44432 |
| 14 | Yukuhashi | 18.73035 | 14 | Shingu | 77.44908 | 14 | Fukutsu | 55.33792 | 14 | Yanagawa | 77.27571 |
| 15 | Chikugo | 18.2255 | 15 | Sue | 77.44908 | 15 | Yame | 54.68037 | 15 | Okawa | 76.97005 |
| 16 | Nakama | 17.6363 | 16 | Umi | 77.44908 | 16 | Miyama | 54.62508 | 16 | Munakata | 76.8966 |
| 17 | Kama | 17.46561 | 17 | Shime | 77.44908 | 17 | Miyako | 53.56111 | 17 | Nakagawa | 76.11351 |
| 18 | Okagaki | 11.82033 | 18 | Ukiha | 77.2942 | 18 | Nogata | 53.24355 | 18 | Onga | 72.9205 |
| 19 | Toho | 11.53345 | 19 | Asakura | 77.2942 | 19 | Chikugo | 52.90059 | 19 | Koga | 72.12828 |
| 20 | Umi | 11.11111 | 20 | Tachiarai | 77.2942 | 20 | Buzen | 52.64056 | 20 | Nakama | 71.62189 |
| 21 | Oki | 10.7844 | 21 | Toho | 77.2942 | 21 | Kurate | 51.17417 | 21 | Kitakyushu | 71.2447 |
| 22 | Yanagawa | 9.63692 | 22 | Ogori | 77.2942 | 22 | Munakata | 51.14314 | 22 | Chikushino | 70.48888 |
| 23 | Koge | 9.05766 | 23 | Chikuzen | 77.2942 | 23 | Nakama | 51.03249 | 23 | Tagawa | 68.8823 |
| 24 | Okawa | 8.65907 | 24 | Fukutsu | 76.01581 | 24 | Chikushino | 50.53242 | 24 | Kurume | 68.75298 |
| 25 | Miyako | 8.4508 | 25 | Okagaki | 76.01581 | 25 | Shime | 50.02803 | 25 | Shime | 68.70578 |
| 26 | Yoshitomi | 7.94278 | 26 | Ashiya | 76.01581 | 26 | Omuta | 49.95734 | 26 | Kasuya | 67.95357 |
| 27 | Kanda | 6.91455 | 27 | Mizumaki | 76.01581 | 27 | Keisen | 49.68895 | 27 | Iizuka | 67.47596 |
| 28 | Fukuoka | 6.79534 | 28 | Onga | 76.01581 | 28 | Kawasaki | 49.55979 | 28 | Onojo | 67.16022 |
| 29 | Ogori | 6.75332 | 29 | Nakama | 76.01581 | 29 | Onojo | 48.89804 | 29 | Miyawaka | 66.41825 |
| 30 | Chikuzen | 6.49221 | 30 | Munakata | 76.01581 | 30 | Soeda | 48.86359 | 30 | Mizumaki | 65.70308 |
| 31 | Nakagawa | 6.22134 | 31 | Kasuga | 68.638 | 31 | Kasuga | 47.89967 | 31 | Nogata | 65.595 |
| 32 | Onga | 6.166 | 32 | Onojo | 68.638 | 32 | Yanagawa | 47.28961 | 32 | Kasuga | 65.47673 |
| 33 | Hirokawa | 6.13066 | 33 | Nakagawa | 68.638 | 33 | Itoshima | 45.00634 | 33 | Fukutsu | 62.74284 |
| 34 | Chikujo | 6.11892 | 34 | Dazaifu | 68.638 | 34 | Fukuoka | 44.87732 | 34 | Dazaifu | 62.19799 |
| 35 | Mizumaki | 5.95592 | 35 | Chikushino | 68.638 | 35 | Kawara | 44.87103 | 35 | Miyama | 62.10557 |
| 36 | Munakata | 5.90659 | 36 | Miyama | 62.73136 | 36 | Ogori | 44.7515 | 36 | Yukuhashi | 61.98714 |
| 37 | Ukiha | 4.46405 | 37 | Yame | 62.73136 | 37 | Ashiya | 42.99072 | 37 | Asakura | 58.5922 |
| 38 | Kurate | 2.21202 | 38 | Okawa | 62.73136 | 38 | Itoda | 42.84911 | 38 | Itoshima | 58.56495 |
| 39 | Ashiya | 2.14436 | 39 | Omuta | 62.73136 | 39 | Kotake | 42.70188 | 39 | Ukiha | 50 |
| 40 | Tagawa | 1.98795 | 40 | Oki | 62.73136 | 40 | Kama | 41.98518 | 40 | Miyako | 50 |
| 41 | Kurume | 1.67618 | 41 | Hirokawa | 62.73136 | 41 | Dazaifu | 41.90262 | 41 | Aka | 50 |
| 42 | Fukuchi | 1.55731 | 42 | Yanagawa | 62.73136 | 42 | Yukuhashi | 41.50769 | 42 | Oto | 50 |
| 43 | Oto | 1.06874 | 43 | Chikugo | 62.73136 | 43 | Mizumaki | 40.00522 | 43 | Tachiarai | 50 |
| 44 | Kawara | 1.05186 | 44 | Miyako | 54.89486 | 44 | Kanda | 39.61942 | 44 | Oki | 50 |
| 45 | Keisen | 0.86682 | 45 | Yukuhashi | 54.89486 | 45 | Sasaguri | 39.39142 | 45 | Toho | 50 |
| 46 | Kotake | 0.77167 | 46 | Yoshitomi | 54.89486 | 46 | Nakagawa | 39.14674 | 46 | Fukuchi | 50 |
| 47 | Itoshima | 0.73565 | 47 | Buzen | 54.89486 | 47 | Yoshitomi | 39.04537 | 47 | Hirokawa | 50 |
| 48 | Sasaguri | 0.71377 | 48 | Koge | 54.89486 | 48 | Kitakyushu | 38.76031 | 48 | Keisen | 50 |
| 49 | Kawasaki | 0.70596 | 49 | Kanda | 54.89486 | 49 | Miyawaka | 38.53292 | 49 | Yoshitomi | 50 |
| 50 | Chikushino | 0.63147 | 50 | Chikujo | 54.89486 | 50 | Umi | 38.13284 | 50 | Buzen | 50 |
| 51 | Sue | 0.57318 | 51 | Kitakyushu | 54.57219 | 51 | Sue | 37.60755 | 51 | Koge | 50 |
| 52 | Fukutsu | 0.40611 | 52 | Aka | 49.12686 | 52 | Hirokawa | 36.25327 | 52 | Soeda | 50 |
| 53 | Tachiarai | 0.39724 | 53 | Kawasaki | 49.12686 | 53 | Kurume | 35.15548 | 53 | Kawara | 50 |
| 54 | Shime | 0.35769 | 54 | Oto | 49.12686 | 54 | Kasuya | 34.71496 | 54 | Kotake | 50 |
| 55 | Kasuga | 0.34527 | 55 | Fukuchi | 49.12686 | 55 | Shingu | 33.90885 | 55 | Sasaguri | 50 |
| 56 | Kasuya | 0.24725 | 56 | Itoda | 49.12686 | 56 | Tagawa | 33.44735 | 56 | Shingu | 50 |
| 57 | Shingu | 0.1669 | 57 | Soeda | 49.12686 | 57 | Ukiha | 31.71215 | 57 | Umi | 50 |
| 58 | Aka | 0.03316 | 58 | Tagawa | 49.12686 | 58 | Koga | 30.29919 | 58 | Chikugo | 50 |
| 59 | Itoda | 0.01137 | 59 | Kawara | 49.12686 | 59 | Hisayama | 27.39151 | 59 | Chikuzen | 50 |
| 60 | Hisayama | 0 | 60 | Fukuoka | 22.15599 | 60 | Asakura | 25.51635 | 60 | Chikujo | 50 |

Supplement Table 1B. Calculated score ranking of 13 key indicators by municipality (#13)

| **#** | **Municipality** | **13.Climate Change** |
| --- | --- | --- |
| 1 | Yukuhashi | 92.79001 |
| 2 | Kitakyushu | 92.16729 |
| 3 | Munakata | 91.35604 |
| 4 | Soeda | 86.4719 |
| 5 | Yame | 82.51571 |
| 6 | Iizuka | 79.70464 |
| 7 | Fukuoka | 76.38881 |
| 8 | Kasuga | 72.5851 |
| 9 | Ashiya | 72.07219 |
| 10 | Chikushino | 72.05357 |
| 11 | Shime | 71.41184 |
| 12 | Fukutsu | 71.35967 |
| 13 | Ogori | 71.35563 |
| 14 | Onojo | 71.01742 |
| 15 | Sasaguri | 70.98627 |
| 16 | Kasuya | 70.97967 |
| 17 | Okagaki | 70.81285 |
| 18 | Chikujo | 70.75604 |
| 19 | Nakagawa | 70.57413 |
| 20 | Toho | 70.47967 |
| 21 | Yanagawa | 70.32389 |
| 22 | Mizumaki | 70.0382 |
| 23 | Umi | 69.79077 |
| 24 | Onga | 69.50288 |
| 25 | Okawa | 69.35036 |
| 26 | Kawara | 69.31648 |
| 27 | Miyama | 69.20613 |
| 28 | Sue | 69.0578 |
| 29 | Tagawa | 69.01837 |
| 30 | Nakama | 69.00302 |
| 31 | Chikuzen | 68.89701 |
| 32 | Oto | 68.54686 |
| 33 | Keisen | 68.53808 |
| 34 | Shingu | 68.4711 |
| 35 | Tachiarai | 68.24071 |
| 36 | Oki | 68.09593 |
| 37 | Kama | 68.0771 |
| 38 | Itoda | 68.00309 |
| 39 | Kawasaki | 67.94744 |
| 40 | Fukuchi | 67.70744 |
| 41 | Aka | 67.6295 |
| 42 | Nogata | 67.62488 |
| 43 | Ukiha | 67.62303 |
| 44 | Koga | 67.30247 |
| 45 | Hirokawa | 66.67073 |
| 46 | Chikugo | 66.23911 |
| 47 | Hisayama | 65.96207 |
| 48 | Kotake | 65.45228 |
| 49 | Miyako | 65.19159 |
| 50 | Buzen | 64.8793 |
| 51 | Koge | 64.54311 |
| 52 | Yoshitomi | 64.50112 |
| 53 | Kurate | 63.47195 |
| 54 | Itoshima | 60 |
| 55 | Dazaifu | 55.41016 |
| 56 | Asakura | 52.81537 |
| 57 | Omuta | 52.31552 |
| 58 | Kurume | 43.93601 |
| 59 | Kanda | 35.70991 |
| 60 | Miyawaka | 26.38888 |

| Supplement Table 1C. Calculated score ranking of 34 measurable indicators by municipality (#1-#4) | | | | | | | | | | | |
| --- | --- | --- | --- | --- | --- | --- | --- | --- | --- | --- | --- |
| **#** | **Municipality** | **1.Forest area** | **#** | **Municipality** | **2.Biochemical Oxygen Demand level** | **#** | **Municipality** | **3.Government revenue** | **#** | **Municipality** | **4.Municipal regulations for pet evacuation during disasters** |
| 1 | Soeda | 100 | 1 | Chikugo | 100 | 1 | Itoda | 100 | 1 | Ukiha | 100 |
| 2 | Toho | 99.72482 | 2 | Ukiha | 95 | 2 | Aka | 98.55072 | 2 | Miyako | 100 |
| 3 | Aka | 85.86488 | 3 | Oto | 92.5 | 3 | Oto | 98.55072 | 3 | Miyama | 100 |
| 4 | Hisayama | 81.44252 | 4 | Koge | 90 | 4 | Toho | 71.01449 | 4 | Kurate | 100 |
| 5 | Sasaguri | 80.01213 | 5 | Miyawaka | 81.25 | 5 | Fukuchi | 71.01449 | 5 | Yame | 100 |
| 6 | Kawara | 78.20683 | 6 | Yanagawa | 80 | 6 | Soeda | 63.76812 | 6 | Kitakyushu | 100 |
| 7 | Yame | 77.97785 | 7 | Miyama | 67.5 | 7 | Kawasaki | 57.97101 | 7 | Asakura | 100 |
| 8 | Miyako | 75.85748 | 8 | Ashiya | 67.5 | 8 | Kama | 42.02899 | 8 | Aka | 100 |
| 9 | Koge | 74.17838 | 9 | Kurume | 61.875 | 9 | Kawara | 36.23188 | 9 | Kawasaki | 100 |
| 10 | Buzen | 73.80091 | 10 | Iizuka | 57.5 | 10 | Miyama | 34.78261 | 10 | Kasuga | 100 |
| 11 | Chikujo | 72.99027 | 11 | Miyako | 55.625 | 11 | Koge | 34.78261 | 11 | Okawa | 100 |
| 12 | Umi | 71.53096 | 12 | Kurate | 55.625 | 12 | Keisen | 30.43478 | 12 | Tachiarai | 100 |
| 13 | Miyawaka | 71.13565 | 13 | Yame | 55.625 | 13 | Tagawa | 28.98551 | 13 | Omuta | 100 |
| 14 | Kama | 69.09933 | 14 | Kitakyushu | 55.625 | 14 | Nakagawa | 27.53623 | 14 | Oki | 100 |
| 15 | Okagaki | 66.66569 | 15 | Asakura | 55.625 | 15 | Nakama | 27.53623 | 15 | Onojo | 100 |
| 16 | Asakura | 65.23813 | 16 | Aka | 55.625 | 16 | Chikujo | 27.53623 | 16 | Toho | 100 |
| 17 | Ukiha | 60.26395 | 17 | Kawasaki | 55.625 | 17 | Miyako | 26.08696 | 17 | Iizuka | 100 |
| 18 | Iizuka | 60.06837 | 18 | Kasuga | 55.625 | 18 | Tachiarai | 26.08696 | 18 | Fukuoka | 100 |
| 19 | Chikushino | 58.96669 | 19 | Okawa | 55.625 | 19 | Ashiya | 26.08696 | 19 | Fukutsu | 100 |
| 20 | Kawasaki | 57.94389 | 20 | Tachiarai | 55.625 | 20 | Kotake | 26.08696 | 20 | Fukuchi | 100 |
| 21 | Itoshima | 54.18745 | 21 | Omuta | 55.625 | 21 | Ukiha | 24.63768 | 21 | Okagaki | 100 |
| 22 | Munakata | 49.88996 | 22 | Oki | 55.625 | 22 | Yame | 24.63768 | 22 | Miyawaka | 100 |
| 23 | Dazaifu | 47.43313 | 23 | Onojo | 55.625 | 23 | Oki | 24.63768 | 23 | Koga | 100 |
| 24 | Kitakyushu | 46.78614 | 24 | Toho | 55.625 | 24 | Yanagawa | 24.63768 | 24 | Hirokawa | 100 |
| 25 | Fukuchi | 46.58065 | 25 | Fukuoka | 55.625 | 25 | Iizuka | 23.18841 | 25 | Keisen | 100 |
| 26 | Sue | 45.89652 | 26 | Fukutsu | 55.625 | 26 | Fukutsu | 23.18841 | 26 | Yukuhashi | 100 |
| 27 | Onojo | 45.03756 | 27 | Okagaki | 55.625 | 27 | Okagaki | 21.73913 | 27 | Yoshitomi | 100 |
| 28 | Hirokawa | 42.41884 | 28 | Koga | 55.625 | 28 | Itoshima | 21.73913 | 28 | Kama | 100 |
| 29 | Kurate | 42.05462 | 29 | Hirokawa | 55.625 | 29 | Mizumaki | 21.73913 | 29 | Kurume | 100 |
| 30 | Kanda | 40.96038 | 30 | Keisen | 55.625 | 30 | Chikuzen | 21.73913 | 30 | Hisayama | 100 |
| 31 | Nogata | 40.8275 | 31 | Yukuhashi | 55.625 | 31 | Munakata | 21.73913 | 31 | Buzen | 100 |
| 32 | Chikuzen | 40.15829 | 32 | Yoshitomi | 55.625 | 32 | Kurate | 20.28986 | 32 | Yanagawa | 100 |
| 33 | Koga | 39.0492 | 33 | Kama | 55.625 | 33 | Kasuga | 18.84058 | 33 | Ashiya | 100 |
| 34 | Fukuoka | 38.21952 | 34 | Hisayama | 55.625 | 34 | Okawa | 18.84058 | 34 | Itoshima | 100 |
| 35 | Keisen | 37.95242 | 35 | Buzen | 55.625 | 35 | Yoshitomi | 18.84058 | 35 | Itoda | 100 |
| 36 | Oto | 37.00572 | 36 | Itoshima | 55.625 | 36 | Dazaifu | 18.84058 | 36 | Nakagawa | 100 |
| 37 | Tagawa | 34.8648 | 37 | Nakagawa | 55.625 | 37 | Onga | 18.84058 | 37 | Kasuya | 100 |
| 38 | Itoda | 33.41124 | 38 | Kasuya | 55.625 | 38 | Kitakyushu | 17.3913 | 38 | Koge | 100 |
| 39 | Shingu | 31.02992 | 39 | Dazaifu | 55.625 | 39 | Onojo | 17.3913 | 39 | Mizumaki | 100 |
| 40 | Fukutsu | 30.7299 | 40 | Soeda | 55.625 | 40 | Yukuhashi | 17.3913 | 40 | Dazaifu | 100 |
| 41 | Kotake | 30.51623 | 41 | Kawara | 55.625 | 41 | Fukuoka | 15.94203 | 41 | Soeda | 100 |
| 42 | Omuta | 27.58638 | 42 | Ogori | 55.625 | 42 | Nogata | 15.94203 | 42 | Tagawa | 100 |
| 43 | Ashiya | 23.98082 | 43 | Kotake | 55.625 | 43 | Omuta | 14.49275 | 43 | Kawara | 100 |
| 44 | Miyama | 22.20751 | 44 | Sasaguri | 55.625 | 44 | Kurume | 14.49275 | 44 | Ogori | 100 |
| 45 | Yukuhashi | 21.99998 | 45 | Shingu | 55.625 | 45 | Buzen | 14.49275 | 45 | Kotake | 100 |
| 46 | Onga | 19.94317 | 46 | Sue | 55.625 | 46 | Ogori | 14.49275 | 46 | Sasaguri | 100 |
| 47 | Kurume | 18.60724 | 47 | Kanda | 55.625 | 47 | Sasaguri | 14.49275 | 47 | Shingu | 100 |
| 48 | Mizumaki | 11.49436 | 48 | Umi | 55.625 | 48 | Shingu | 14.49275 | 48 | Kanda | 100 |
| 49 | Kasuya | 6.08354 | 49 | Shime | 55.625 | 49 | Chikushino | 14.49275 | 49 | Umi | 100 |
| 50 | Nakama | 6.05924 | 50 | Chikuzen | 55.625 | 50 | Umi | 13.04348 | 50 | Onga | 100 |
| 51 | Kasuga | 5.31558 | 51 | Chikushino | 55.625 | 51 | Shime | 13.04348 | 51 | Nogata | 100 |
| 52 | Nakagawa | 3.58216 | 52 | Chikujo | 55.625 | 52 | Sue | 11.5942 | 52 | Shime | 100 |
| 53 | Shime | 3.43468 | 53 | Munakata | 55.625 | 53 | Chikugo | 11.5942 | 53 | Nakama | 100 |
| 54 | Ogori | 3.14804 | 54 | Fukuchi | 50 | 54 | Asakura | 10.14493 | 54 | Chikugo | 100 |
| 55 | Chikugo | 0.20003 | 55 | Nogata | 40 | 55 | Hirokawa | 10.14493 | 55 | Chikuzen | 100 |
| 56 | Tachiarai | 0.05227 | 56 | Onga | 38.74999 | 56 | Kasuya | 10.14493 | 56 | Chikushino | 100 |
| 57 | Okawa | 0 | 57 | Itoda | 9.99999 | 57 | Miyawaka | 8.69565 | 57 | Chikujo | 100 |
| 58 | Oki | 0 | 58 | Tagawa | 9.99999 | 58 | Koga | 8.69565 | 58 | Munakata | 100 |
| 59 | Yoshitomi | 0 | 59 | Mizumaki | 3.74999 | 59 | Hisayama | 7.24638 | 59 | Oto | 0 |
| 60 | Yanagawa | 0 | 60 | Nakama | 0 | 60 | Kanda | 0 | 60 | Sue | 0 |

| Supplement Table 1C. Calculated score ranking of 34 measurable indicators by municipality (#5-#8) | | | | | | | | | | | |
| --- | --- | --- | --- | --- | --- | --- | --- | --- | --- | --- | --- |
| **#** | **Municipality** | **5.Unemployment** | **#** | **Municipality** | **6.Natural population growth rate** | **#** | **Municipality** | **7.Renewable electricity generation** | **#** | **Municipality** | **8.Number of hospital and clinic beds** |
| 1 | Hisayama | 100 | 1 | Kasuya | 100 | 1 | Buzen | 100 | 1 | Tagawa | 100 |
| 2 | Shingu | 100 | 2 | Shingu | 86.98885 | 2 | Kanda | 82.07329 | 2 | Omuta | 91.5383 |
| 3 | Oki | 98.46154 | 3 | Onojo | 86.24535 | 3 | Omuta | 62.18271 | 3 | Sasaguri | 55.80957 |
| 4 | Toho | 98.46154 | 4 | Tachiarai | 80.2974 | 4 | Kawasaki | 61.15094 | 4 | Koga | 55.08106 |
| 5 | Tachiarai | 96.92308 | 5 | Chikushino | 79.92565 | 5 | Miyako | 47.45678 | 5 | Hirokawa | 54.52584 |
| 6 | Yame | 95.38461 | 6 | Kasuga | 79.5539 | 6 | Aka | 40.99591 | 6 | Fukuchi | 54.32227 |
| 7 | Hirokawa | 95.38461 | 7 | Fukuoka | 79.5539 | 7 | Kama | 37.65845 | 7 | Kama | 53.99839 |
| 8 | Chikugo | 93.84615 | 8 | Shime | 79.18216 | 8 | Kitakyushu | 28.72903 | 8 | Soeda | 53.68627 |
| 9 | Asakura | 90.76923 | 9 | Fukutsu | 76.95167 | 9 | Koge | 25.97848 | 9 | Kurume | 52.34155 |
| 10 | Kasuya | 90.76923 | 10 | Sue | 76.95167 | 10 | Itoda | 25.593 | 10 | Yame | 51.93919 |
| 11 | Kanda | 90.76923 | 11 | Nakagawa | 75.83643 | 11 | Soeda | 25.0915 | 11 | Miyawaka | 51.5445 |
| 12 | Fukutsu | 89.23077 | 12 | Hisayama | 75.46468 | 12 | Oto | 24.65354 | 12 | Iizuka | 50.25508 |
| 13 | Koga | 89.23077 | 13 | Chikugo | 75.09294 | 13 | Miyama | 22.34814 | 13 | Kitakyushu | 46.26644 |
| 14 | Munakata | 87.69231 | 14 | Sasaguri | 74.72119 | 14 | Keisen | 20.37857 | 14 | Okawa | 44.29698 |
| 15 | Miyama | 86.15385 | 15 | Kanda | 73.97769 | 15 | Kotake | 20.03445 | 15 | Ogori | 43.26869 |
| 16 | Aka | 86.15385 | 16 | Koga | 72.49071 | 16 | Chikuzen | 17.71206 | 16 | Buzen | 42.24187 |
| 17 | Onojo | 86.15385 | 17 | Umi | 70.63197 | 17 | Toho | 14.9154 | 17 | Onga | 39.42751 |
| 18 | Shime | 86.15385 | 18 | Oki | 69.14498 | 18 | Chikujo | 14.76857 | 18 | Mizumaki | 38.82512 |
| 19 | Ukiha | 84.61539 | 19 | Dazaifu | 69.14498 | 19 | Munakata | 14.11525 | 19 | Yukuhashi | 38.62512 |
| 20 | Kurume | 84.61539 | 20 | Munakata | 67.28625 | 20 | Fukuchi | 13.82789 | 20 | Nogata | 38.55792 |
| 21 | Ogori | 84.61539 | 21 | Kurume | 66.9145 | 21 | Kawara | 12.84885 | 21 | Asakura | 36.45488 |
| 22 | Umi | 84.61539 | 22 | Chikuzen | 66.9145 | 22 | Yame | 11.80422 | 22 | Kawasaki | 36.1984 |
| 23 | Chikuzen | 84.61539 | 23 | Yukuhashi | 65.42751 | 23 | Fukutsu | 10.89782 | 23 | Fukutsu | 36.17126 |
| 24 | Yukuhashi | 81.53846 | 24 | Itoda | 65.05576 | 24 | Miyawaka | 10.39943 | 24 | Yanagawa | 35.90762 |
| 25 | Koge | 81.53846 | 25 | Ogori | 64.31227 | 25 | Hirokawa | 9.12764 | 25 | Umi | 35.41663 |
| 26 | Sue | 81.53846 | 26 | Hirokawa | 58.73606 | 26 | Kurate | 9.11385 | 26 | Onojo | 34.76846 |
| 27 | Kitakyushu | 80 | 27 | Kitakyushu | 57.24907 | 27 | Ukiha | 8.76283 | 27 | Chikuzen | 34.68235 |
| 28 | Kasuga | 80 | 28 | Onga | 57.24907 | 28 | Nogata | 8.743 | 28 | Kurate | 34.51883 |
| 29 | Okawa | 80 | 29 | Iizuka | 56.13383 | 29 | Onga | 8.69064 | 29 | Okagaki | 33.19377 |
| 30 | Onga | 80 | 30 | Mizumaki | 55.39033 | 30 | Kasuga | 7.61296 | 30 | Fukuoka | 31.82915 |
| 31 | Chikujo | 80 | 31 | Okagaki | 55.01859 | 31 | Okagaki | 7.25399 | 31 | Miyama | 31.50894 |
| 32 | Okagaki | 78.46154 | 32 | Nogata | 52.41636 | 32 | Tagawa | 6.7386 | 32 | Chikushino | 31.23726 |
| 33 | Buzen | 78.46154 | 33 | Ashiya | 50.55762 | 33 | Asakura | 6.66673 | 33 | Munakata | 30.93468 |
| 34 | Nakagawa | 78.46154 | 34 | Keisen | 50.18587 | 34 | Yukuhashi | 6.61916 | 34 | Ukiha | 30.75759 |
| 35 | Chikushino | 78.46154 | 35 | Yoshitomi | 49.81413 | 35 | Nakagawa | 6.46281 | 35 | Shime | 30.44189 |
| 36 | Miyawaka | 76.92308 | 36 | Koge | 48.69888 | 36 | Yanagawa | 6.32578 | 36 | Kanda | 29.12426 |
| 37 | Yoshitomi | 76.92308 | 37 | Yame | 47.58364 | 37 | Itoshima | 5.67549 | 37 | Itoda | 27.08164 |
| 38 | Itoshima | 76.92308 | 38 | Ukiha | 46.4684 | 38 | Tachiarai | 5.5417 | 38 | Ashiya | 25.05008 |
| 39 | Fukuoka | 75.38462 | 39 | Asakura | 45.72491 | 39 | Oki | 5.48554 | 39 | Itoshima | 23.49597 |
| 40 | Yanagawa | 73.84615 | 40 | Yanagawa | 45.35316 | 40 | Okawa | 5.13737 | 40 | Hisayama | 22.71842 |
| 41 | Dazaifu | 72.30769 | 41 | Okawa | 44.98141 | 41 | Ashiya | 4.30378 | 41 | Yoshitomi | 22.53961 |
| 42 | Nogata | 72.30769 | 42 | Tagawa | 43.49442 | 42 | Ogori | 3.97803 | 42 | Kotake | 22.46489 |
| 43 | Ashiya | 70.76923 | 43 | Chikujo | 43.12268 | 43 | Chikugo | 3.90745 | 43 | Chikugo | 22.01666 |
| 44 | Miyako | 69.23077 | 44 | Kurate | 40.52044 | 44 | Sue | 3.60133 | 44 | Dazaifu | 21.62513 |
| 45 | Omuta | 69.23077 | 45 | Miyawaka | 39.4052 | 45 | Chikushino | 3.47539 | 45 | Kasuga | 21.5777 |
| 46 | Iizuka | 69.23077 | 46 | Miyama | 39.03346 | 46 | Nakama | 3.29028 | 46 | Oki | 21.5705 |
| 47 | Sasaguri | 67.69231 | 47 | Nakama | 37.91822 | 47 | Kurume | 3.21241 | 47 | Kasuya | 19.89868 |
| 48 | Keisen | 61.53846 | 48 | Buzen | 35.31598 | 48 | Koga | 2.96491 | 48 | Sue | 18.79119 |
| 49 | Tagawa | 50.76923 | 49 | Miyako | 34.57249 | 49 | Umi | 2.7843 | 49 | Miyako | 17.98788 |
| 50 | Kurate | 49.23077 | 50 | Omuta | 34.57249 | 50 | Mizumaki | 2.53518 | 50 | Tachiarai | 17.50513 |
| 51 | Soeda | 49.23077 | 51 | Fukuchi | 34.20074 | 51 | Sasaguri | 2.44922 | 51 | Keisen | 15.51607 |
| 52 | Kama | 47.69231 | 52 | Kawasaki | 32.71375 | 52 | Shingu | 2.22067 | 52 | Nakama | 12.36174 |
| 53 | Nakama | 44.61539 | 53 | Itoshima | 30.11152 | 53 | Dazaifu | 1.55109 | 53 | Nakagawa | 9.16736 |
| 54 | Kotake | 30.76924 | 54 | Oto | 29.36803 | 54 | Hisayama | 1.5288 | 54 | Shingu | 8.1416 |
| 55 | Itoda | 29.23078 | 55 | Kama | 28.62453 | 55 | Kasuya | 1.50691 | 55 | Oto | 7.98853 |
| 56 | Kawara | 20 | 56 | Kawara | 23.42007 | 56 | Yoshitomi | 1.3596 | 56 | Chikujo | 7.53541 |
| 57 | Oto | 9.23077 | 57 | Soeda | 23.04833 | 57 | Shime | 1.26225 | 57 | Kawara | 3.93117 |
| 58 | Kawasaki | 6.15385 | 58 | Aka | 21.56134 | 58 | Onojo | 1.18014 | 58 | Aka | 0 |
| 59 | Mizumaki | 3.07693 | 59 | Kotake | 18.21561 | 59 | Fukuoka | 0.93798 | 59 | Toho | 0 |
| 60 | Fukuchi | 0 | 60 | Toho | 0 | 60 | Iizuka | 0 | 60 | Koge | 0 |

| Supplement Table 1C. Calculated score ranking of 34 measurable indicators by municipality (#9-#12) | | | | | | | | | | | |
| --- | --- | --- | --- | --- | --- | --- | --- | --- | --- | --- | --- |
| **#** | **Municipality** | **9.Doctors** | **#** | **Municipality** | **10.Suicide rate** | **#** | **Municipality** | **11.Livestock farm inspection implementation rate** | **#** | **Municipality** | **12.Wild animal disease surveillance system** |
| 1 | Kurume | 100 | 1 | Aka | 100 | 1 | Ukiha | 100 | 1 | Ukiha | 100 |
| 2 | Iizuka | 97.54728 | 2 | Oto | 100 | 2 | Miyako | 100 | 2 | Miyako | 100 |
| 3 | Okawa | 84.98301 | 3 | Yoshitomi | 100 | 3 | Miyama | 100 | 3 | Miyama | 100 |
| 4 | Tagawa | 67.99818 | 4 | Buzen | 100 | 4 | Kurate | 100 | 4 | Kurate | 100 |
| 5 | Kitakyushu | 61.48781 | 5 | Itoda | 100 | 5 | Yame | 100 | 5 | Yame | 100 |
| 6 | Chikushino | 59.27292 | 6 | Koge | 100 | 6 | Kitakyushu | 100 | 6 | Kitakyushu | 100 |
| 7 | Omuta | 56.98315 | 7 | Kawara | 100 | 7 | Asakura | 100 | 7 | Asakura | 100 |
| 8 | Koga | 56.22393 | 8 | Kotake | 100 | 8 | Aka | 100 | 8 | Aka | 100 |
| 9 | Mizumaki | 55.60679 | 9 | Kama | 89.86542 | 9 | Kawasaki | 100 | 9 | Kawasaki | 100 |
| 10 | Fukuoka | 55.07312 | 10 | Shingu | 89.41463 | 10 | Kasuga | 100 | 10 | Kasuga | 100 |
| 11 | Onga | 48.14856 | 11 | Koga | 85.09041 | 11 | Oto | 100 | 11 | Oto | 100 |
| 12 | Kasuga | 44.58734 | 12 | Shime | 84.87571 | 12 | Okawa | 100 | 12 | Okawa | 100 |
| 13 | Yame | 41.12201 | 13 | Chikujo | 82.13447 | 13 | Tachiarai | 100 | 13 | Tachiarai | 100 |
| 14 | Hirokawa | 36.55351 | 14 | Ukiha | 81.21694 | 14 | Omuta | 100 | 14 | Omuta | 100 |
| 15 | Ashiya | 36.2164 | 15 | Itoshima | 80.95606 | 15 | Oki | 100 | 15 | Oki | 100 |
| 16 | Shime | 35.92666 | 16 | Miyama | 80.42443 | 16 | Onojo | 100 | 16 | Onojo | 100 |
| 17 | Asakura | 34.03606 | 17 | Asakura | 79.15871 | 17 | Toho | 100 | 17 | Toho | 100 |
| 18 | Ogori | 33.73316 | 18 | Tachiarai | 77.95178 | 18 | Iizuka | 100 | 18 | Iizuka | 100 |
| 19 | Fukutsu | 33.65192 | 19 | Nogata | 77.93404 | 19 | Fukuoka | 100 | 19 | Fukuoka | 100 |
| 20 | Kasuya | 30.7105 | 20 | Sasaguri | 77.36453 | 20 | Fukutsu | 100 | 20 | Fukutsu | 100 |
| 21 | Kurate | 26.03636 | 21 | Tagawa | 77.09948 | 21 | Fukuchi | 100 | 21 | Fukuchi | 100 |
| 22 | Kanda | 25.89627 | 22 | Chikugo | 74.95299 | 22 | Okagaki | 100 | 22 | Okagaki | 100 |
| 23 | Miyawaka | 25.62072 | 23 | Munakata | 74.62866 | 23 | Miyawaka | 100 | 23 | Miyawaka | 100 |
| 24 | Yukuhashi | 25.1811 | 24 | Mizumaki | 74.607 | 24 | Koga | 100 | 24 | Koga | 100 |
| 25 | Nogata | 22.93204 | 25 | Omuta | 74.41218 | 25 | Hirokawa | 100 | 25 | Hirokawa | 100 |
| 26 | Sasaguri | 22.68377 | 26 | Fukutsu | 74.2424 | 26 | Keisen | 100 | 26 | Keisen | 100 |
| 27 | Chikugo | 22.31151 | 27 | Chikushino | 73.49441 | 27 | Yukuhashi | 100 | 27 | Yukuhashi | 100 |
| 28 | Kama | 21.81703 | 28 | Hirokawa | 72.64324 | 28 | Yoshitomi | 100 | 28 | Yoshitomi | 100 |
| 29 | Kawasaki | 21.41347 | 29 | Keisen | 72.51104 | 29 | Kama | 100 | 29 | Kama | 100 |
| 30 | Yanagawa | 19.64484 | 30 | Yanagawa | 72.43326 | 30 | Kurume | 100 | 30 | Kurume | 100 |
| 31 | Miyama | 17.43764 | 31 | Onojo | 72.25069 | 31 | Hisayama | 100 | 31 | Hisayama | 100 |
| 32 | Munakata | 16.14316 | 32 | Nakagawa | 71.84839 | 32 | Buzen | 100 | 32 | Buzen | 100 |
| 33 | Tachiarai | 13.09349 | 33 | Umi | 71.54556 | 33 | Yanagawa | 100 | 33 | Yanagawa | 100 |
| 34 | Itoshima | 12.85156 | 34 | Kasuya | 70.96779 | 34 | Ashiya | 100 | 34 | Ashiya | 100 |
| 35 | Fukuchi | 12.22014 | 35 | Yame | 70.84394 | 35 | Itoshima | 100 | 35 | Itoshima | 100 |
| 36 | Itoda | 12.00387 | 36 | Kurume | 70.84144 | 36 | Itoda | 100 | 36 | Itoda | 100 |
| 37 | Okagaki | 11.63501 | 37 | Ogori | 70.49169 | 37 | Nakagawa | 100 | 37 | Nakagawa | 100 |
| 38 | Hisayama | 11.32862 | 38 | Dazaifu | 70.41247 | 38 | Kasuya | 100 | 38 | Kasuya | 100 |
| 39 | Chikuzen | 11.26487 | 39 | Kasuga | 68.71628 | 39 | Koge | 100 | 39 | Koge | 100 |
| 40 | Yoshitomi | 11.23945 | 40 | Fukuoka | 67.27886 | 40 | Mizumaki | 100 | 40 | Mizumaki | 100 |
| 41 | Ukiha | 11.15445 | 41 | Kawasaki | 66.71543 | 41 | Dazaifu | 100 | 41 | Dazaifu | 100 |
| 42 | Buzen | 10.65308 | 42 | Iizuka | 66.08814 | 42 | Soeda | 100 | 42 | Soeda | 100 |
| 43 | Nakama | 10.38185 | 43 | Kitakyushu | 65.07322 | 43 | Tagawa | 100 | 43 | Tagawa | 100 |
| 44 | Onojo | 9.78448 | 44 | Chikuzen | 64.98031 | 44 | Kawara | 100 | 44 | Kawara | 100 |
| 45 | Dazaifu | 9.5175 | 45 | Yukuhashi | 63.65488 | 45 | Ogori | 100 | 45 | Ogori | 100 |
| 46 | Umi | 9.15301 | 46 | Onga | 62.93591 | 46 | Kotake | 100 | 46 | Kotake | 100 |
| 47 | Miyako | 8.66041 | 47 | Oki | 61.92421 | 47 | Sasaguri | 100 | 47 | Sasaguri | 100 |
| 48 | Kotake | 7.26629 | 48 | Okawa | 61.83907 | 48 | Shingu | 100 | 48 | Shingu | 100 |
| 49 | Shingu | 7.07197 | 49 | Soeda | 61.01223 | 49 | Sue | 100 | 49 | Sue | 100 |
| 50 | Sue | 6.29683 | 50 | Ashiya | 59.3433 | 50 | Kanda | 100 | 50 | Kanda | 100 |
| 51 | Soeda | 5.78829 | 51 | Sue | 57.58688 | 51 | Umi | 100 | 51 | Umi | 100 |
| 52 | Oki | 5.65289 | 52 | Okagaki | 55.21766 | 52 | Onga | 100 | 52 | Onga | 100 |
| 53 | Keisen | 2.04056 | 53 | Miyawaka | 53.53841 | 53 | Nogata | 100 | 53 | Nogata | 100 |
| 54 | Chikujo | 1.3262 | 54 | Nakama | 51.9243 | 54 | Shime | 100 | 54 | Shime | 100 |
| 55 | Nakagawa | 0.52244 | 55 | Kanda | 43.42887 | 55 | Nakama | 100 | 55 | Nakama | 100 |
| 56 | Aka | 0 | 56 | Fukuchi | 42.38276 | 56 | Chikugo | 100 | 56 | Chikugo | 100 |
| 57 | Oto | 0 | 57 | Kurate | 29.85149 | 57 | Chikuzen | 100 | 57 | Chikuzen | 100 |
| 58 | Toho | 0 | 58 | Hisayama | 23.69465 | 58 | Chikushino | 100 | 58 | Chikushino | 100 |
| 59 | Koge | 0 | 59 | Toho | 6.05133 | 59 | Chikujo | 100 | 59 | Chikujo | 100 |
| 60 | Kawara | 0 | 60 | Miyako | 0 | 60 | Munakata | 100 | 60 | Munakata | 100 |

| Supplement Table 1C. Calculated score ranking of 34 measurable indicators by municipality (#13-#16) | | | | | | | | | | | |
| --- | --- | --- | --- | --- | --- | --- | --- | --- | --- | --- | --- |
| **#** | **Municipality** | **13.Recycling rate** | **#** | **Municipality** | **14.One Health policy agreement in council** | **#** | **Municipality** | **15.One Health declaration** | **#** | **Municipality** | **16.Registered agricultural enterprises implementing One Health practices** |
| 1 | Miyawaka | 100 | 1 | Miyama | 100 | 1 | Miyako | 100 | 1 | Yame | 100 |
| 2 | Kotake | 99.89247 | 2 | Kitakyushu | 100 | 2 | Miyama | 100 | 2 | Miyama | 61.84792 |
| 3 | Kurate | 99.78495 | 3 | Asakura | 100 | 3 | Yame | 100 | 3 | Ukiha | 55.47652 |
| 4 | Kanda | 73.65591 | 4 | Fukuoka | 100 | 4 | Kitakyushu | 100 | 4 | Oki | 50.89543 |
| 5 | Umi | 70.96774 | 5 | Okagaki | 100 | 5 | Asakura | 100 | 5 | Asakura | 27.15481 |
| 6 | Oki | 70.86022 | 6 | Miyawaka | 100 | 6 | Okawa | 100 | 6 | Yanagawa | 21.82972 |
| 7 | Ukiha | 70.21506 | 7 | Nakagawa | 100 | 7 | Omuta | 100 | 7 | Yukuhashi | 12.39088 |
| 8 | Sasaguri | 69.13979 | 8 | Dazaifu | 100 | 8 | Oki | 100 | 8 | Itoshima | 7.34571 |
| 9 | Sue | 68.70968 | 9 | Umi | 100 | 9 | Onojo | 100 | 9 | Buzen | 6.14327 |
| 10 | Shime | 67.09678 | 10 | Nogata | 100 | 10 | Toho | 100 | 10 | Ogori | 4.8109 |
| 11 | Kasuya | 66.12903 | 11 | Chikugo | 100 | 11 | Iizuka | 100 | 11 | Kurume | 4.0216 |
| 12 | Omuta | 58.49462 | 12 | Munakata | 100 | 12 | Okagaki | 100 | 12 | Kanda | 3.05527 |
| 13 | Chikujo | 50.75269 | 13 | Ukiha | 0 | 13 | Miyawaka | 100 | 13 | Iizuka | 2.46107 |
| 14 | Miyama | 41.39785 | 14 | Miyako | 0 | 14 | Koga | 100 | 14 | Fukuoka | 1.63738 |
| 15 | Kama | 29.13979 | 15 | Kurate | 0 | 15 | Hirokawa | 100 | 15 | Tagawa | 1.12085 |
| 16 | Toho | 28.27957 | 16 | Yame | 0 | 16 | Yukuhashi | 100 | 16 | Chikushino | 1.0233 |
| 17 | Ogori | 27.63441 | 17 | Aka | 0 | 17 | Yoshitomi | 100 | 17 | Toho | 0.95136 |
| 18 | Iizuka | 26.98925 | 18 | Kawasaki | 0 | 18 | Kama | 100 | 18 | Tachiarai | 0.89308 |
| 19 | Fukutsu | 26.77419 | 19 | Kasuga | 0 | 19 | Buzen | 100 | 19 | Soeda | 0.78961 |
| 20 | Tachiarai | 26.55914 | 20 | Oto | 0 | 20 | Yanagawa | 100 | 20 | Aka | 0.59683 |
| 21 | Kitakyushu | 24.83871 | 21 | Okawa | 0 | 21 | Koge | 100 | 21 | Kitakyushu | 0.53729 |
| 22 | Munakata | 24.4086 | 22 | Tachiarai | 0 | 22 | Mizumaki | 100 | 22 | Onga | 0.46916 |
| 23 | Itoshima | 23.87097 | 23 | Omuta | 0 | 23 | Dazaifu | 100 | 23 | Kasuya | 0.33074 |
| 24 | Chikuzen | 23.22581 | 24 | Oki | 0 | 24 | Soeda | 100 | 24 | Chikuzen | 0.29552 |
| 25 | Chikushino | 22.7957 | 25 | Onojo | 0 | 25 | Ogori | 100 | 25 | Nogata | 0.28729 |
| 26 | Okawa | 22.68817 | 26 | Toho | 0 | 26 | Kanda | 100 | 26 | Itoda | 0.20469 |
| 27 | Kurume | 21.93548 | 27 | Iizuka | 0 | 27 | Umi | 100 | 27 | Okawa | 0.16561 |
| 28 | Onojo | 20.75269 | 28 | Fukutsu | 0 | 28 | Onga | 100 | 28 | Munakata | 0.14681 |
| 29 | Yanagawa | 20.64516 | 29 | Fukuchi | 0 | 29 | Nogata | 100 | 29 | Ashiya | 0.13724 |
| 30 | Ashiya | 19.78495 | 30 | Koga | 0 | 30 | Nakama | 100 | 30 | Kawasaki | 0.11235 |
| 31 | Okagaki | 19.67742 | 31 | Hirokawa | 0 | 31 | Chikugo | 100 | 31 | Chikugo | 0.1087 |
| 32 | Onga | 19.24731 | 32 | Keisen | 0 | 32 | Chikuzen | 100 | 32 | Nakama | 0.08852 |
| 33 | Nakama | 19.13978 | 33 | Yukuhashi | 0 | 33 | Chikujo | 100 | 33 | Miyako | 0.08439 |
| 34 | Asakura | 19.03226 | 34 | Yoshitomi | 0 | 34 | Ukiha | 0 | 34 | Okagaki | 0.05669 |
| 35 | Keisen | 18.27957 | 35 | Kama | 0 | 35 | Kurate | 0 | 35 | Dazaifu | 0.02497 |
| 36 | Mizumaki | 17.84946 | 36 | Kurume | 0 | 36 | Aka | 0 | 36 | Kurate | 0 |
| 37 | Dazaifu | 17.52688 | 37 | Hisayama | 0 | 37 | Kawasaki | 0 | 37 | Kasuga | 0 |
| 38 | Koga | 16.66667 | 38 | Buzen | 0 | 38 | Kasuga | 0 | 38 | Oto | 0 |
| 39 | Kasuga | 15.05376 | 39 | Yanagawa | 0 | 39 | Oto | 0 | 39 | Omuta | 0 |
| 40 | Nakagawa | 14.19355 | 40 | Ashiya | 0 | 40 | Tachiarai | 0 | 40 | Onojo | 0 |
| 41 | Fukuchi | 13.76344 | 41 | Itoshima | 0 | 41 | Fukuoka | 0 | 41 | Fukutsu | 0 |
| 42 | Yame | 13.54839 | 42 | Itoda | 0 | 42 | Fukutsu | 0 | 42 | Fukuchi | 0 |
| 43 | Chikugo | 13.11828 | 43 | Kasuya | 0 | 43 | Fukuchi | 0 | 43 | Miyawaka | 0 |
| 44 | Itoda | 12.7957 | 44 | Koge | 0 | 44 | Keisen | 0 | 44 | Koga | 0 |
| 45 | Yukuhashi | 12.68817 | 45 | Mizumaki | 0 | 45 | Kurume | 0 | 45 | Hirokawa | 0 |
| 46 | Fukuoka | 12.04301 | 46 | Soeda | 0 | 46 | Hisayama | 0 | 46 | Keisen | 0 |
| 47 | Koge | 11.1828 | 47 | Tagawa | 0 | 47 | Ashiya | 0 | 47 | Yoshitomi | 0 |
| 48 | Shingu | 11.1828 | 48 | Kawara | 0 | 48 | Itoshima | 0 | 48 | Kama | 0 |
| 49 | Yoshitomi | 10.43011 | 49 | Ogori | 0 | 49 | Itoda | 0 | 49 | Hisayama | 0 |
| 50 | Hirokawa | 10.32258 | 50 | Kotake | 0 | 50 | Nakagawa | 0 | 50 | Nakagawa | 0 |
| 51 | Nogata | 9.67742 | 51 | Sasaguri | 0 | 51 | Kasuya | 0 | 51 | Koge | 0 |
| 52 | Buzen | 8.49462 | 52 | Shingu | 0 | 52 | Tagawa | 0 | 52 | Mizumaki | 0 |
| 53 | Tagawa | 7.74194 | 53 | Sue | 0 | 53 | Kawara | 0 | 53 | Kawara | 0 |
| 54 | Aka | 6.66667 | 54 | Kanda | 0 | 54 | Kotake | 0 | 54 | Kotake | 0 |
| 55 | Kawara | 6.66667 | 55 | Onga | 0 | 55 | Sasaguri | 0 | 55 | Sasaguri | 0 |
| 56 | Soeda | 5.26882 | 56 | Shime | 0 | 56 | Shingu | 0 | 56 | Shingu | 0 |
| 57 | Kawasaki | 3.44086 | 57 | Nakama | 0 | 57 | Sue | 0 | 57 | Sue | 0 |
| 58 | Miyako | 3.33333 | 58 | Chikuzen | 0 | 58 | Shime | 0 | 58 | Umi | 0 |
| 59 | Oto | 2.25806 | 59 | Chikushino | 0 | 59 | Chikushino | 0 | 59 | Shime | 0 |
| 60 | Hisayama | 0 | 60 | Chikujo | 0 | 60 | Munakata | 0 | 60 | Chikujo | 0 |

| Supplement Table 1C. Calculated score ranking of 34 measurable indicators by municipality (#17-#20) | | | | | | | | | | | |
| --- | --- | --- | --- | --- | --- | --- | --- | --- | --- | --- | --- |
| **#** | **Municipality** | **17.Registered One Health declaration businesses** | **#** | **Municipality** | **18.One Health website presence scale** | **#** | **Municipality** | **19.One Health awareness certified facilities** | **#** | **Municipality** | **20.Influenza vaccination rate for age 65+** |
| 1 | Miyama | 100 | 1 | Miyama | 100 | 1 | Miyama | 100 | 1 | Soeda | 67.21146 |
| 2 | Chikugo | 42.6501 | 2 | Yame | 66.66667 | 2 | Omuta | 50 | 2 | Chikuzen | 65.92398 |
| 3 | Toho | 35.55023 | 3 | Onojo | 66.66667 | 3 | Soeda | 50 | 3 | Kotake | 65.82781 |
| 4 | Miyawaka | 21.34847 | 4 | Koga | 66.66667 | 4 | Nogata | 50 | 4 | Koge | 65.16105 |
| 5 | Koge | 21.01265 | 5 | Nogata | 66.66667 | 5 | Ukiha | 0 | 5 | Kawasaki | 65.04227 |
| 6 | Okawa | 18.56586 | 6 | Kitakyushu | 33.33333 | 6 | Miyako | 0 | 6 | Yanagawa | 65.03593 |
| 7 | Miyako | 17.34336 | 7 | Asakura | 33.33333 | 7 | Kurate | 0 | 7 | Asakura | 64.78974 |
| 8 | Yanagawa | 17.21159 | 8 | Iizuka | 33.33333 | 8 | Yame | 0 | 8 | Keisen | 64.08768 |
| 9 | Buzen | 14.81029 | 9 | Miyawaka | 33.33333 | 9 | Kitakyushu | 0 | 9 | Aka | 64.0033 |
| 10 | Oki | 14.4079 | 10 | Yukuhashi | 33.33333 | 10 | Asakura | 0 | 10 | Kawara | 63.95056 |
| 11 | Yoshitomi | 14.32336 | 11 | Kama | 33.33333 | 11 | Aka | 0 | 11 | Miyama | 63.6599 |
| 12 | Kurate | 13.27212 | 12 | Buzen | 33.33333 | 12 | Kawasaki | 0 | 12 | Fukuchi | 62.78802 |
| 13 | Asakura | 13.14393 | 13 | Dazaifu | 33.33333 | 13 | Kasuga | 0 | 13 | Tagawa | 62.74833 |
| 14 | Ashiya | 12.82043 | 14 | Nakama | 33.33333 | 14 | Oto | 0 | 14 | Ogori | 62.66238 |
| 15 | Iizuka | 11.76291 | 15 | Ukiha | 0 | 15 | Okawa | 0 | 15 | Tachiarai | 62.64765 |
| 16 | Yame | 11.5843 | 16 | Miyako | 0 | 16 | Tachiarai | 0 | 16 | Miyawaka | 61.93427 |
| 17 | Tagawa | 11.55409 | 17 | Kurate | 0 | 17 | Oki | 0 | 17 | Buzen | 61.91638 |
| 18 | Soeda | 11.06474 | 18 | Aka | 0 | 18 | Onojo | 0 | 18 | Yame | 61.8646 |
| 19 | Nogata | 10.13901 | 19 | Kawasaki | 0 | 19 | Toho | 0 | 19 | Toho | 61.74496 |
| 20 | Fukuchi | 9.34388 | 20 | Kasuga | 0 | 20 | Iizuka | 0 | 20 | Chikujo | 61.70698 |
| 21 | Kurume | 8.71656 | 21 | Oto | 0 | 21 | Fukuoka | 0 | 21 | Yoshitomi | 61.39933 |
| 22 | Ukiha | 8.29211 | 22 | Okawa | 0 | 22 | Fukutsu | 0 | 22 | Kurate | 61.32375 |
| 23 | Yukuhashi | 8.25181 | 23 | Tachiarai | 0 | 23 | Fukuchi | 0 | 23 | Yukuhashi | 61.23129 |
| 24 | Kanda | 7.13552 | 24 | Omuta | 0 | 24 | Okagaki | 0 | 24 | Hirokawa | 61.15499 |
| 25 | Fukuoka | 6.8929 | 25 | Oki | 0 | 25 | Miyawaka | 0 | 25 | Okagaki | 61.10953 |
| 26 | Oto | 6.41243 | 26 | Toho | 0 | 26 | Koga | 0 | 26 | Fukutsu | 60.96827 |
| 27 | Kawara | 6.31115 | 27 | Fukuoka | 0 | 27 | Hirokawa | 0 | 27 | Nakama | 60.88262 |
| 28 | Nakama | 5.78832 | 28 | Fukutsu | 0 | 28 | Keisen | 0 | 28 | Miyako | 60.86503 |
| 29 | Omuta | 5.74895 | 29 | Fukuchi | 0 | 29 | Yukuhashi | 0 | 29 | Iizuka | 60.83435 |
| 30 | Ogori | 5.58298 | 30 | Okagaki | 0 | 30 | Yoshitomi | 0 | 30 | Kama | 60.5285 |
| 31 | Chikuzen | 5.52145 | 31 | Hirokawa | 0 | 31 | Kama | 0 | 31 | Dazaifu | 60.23145 |
| 32 | Keisen | 5.20092 | 32 | Keisen | 0 | 32 | Kurume | 0 | 32 | Munakata | 60.02047 |
| 33 | Kama | 4.79366 | 33 | Yoshitomi | 0 | 33 | Hisayama | 0 | 33 | Kitakyushu | 60.01115 |
| 34 | Dazaifu | 4.66497 | 34 | Kurume | 0 | 34 | Buzen | 0 | 34 | Ashiya | 59.88871 |
| 35 | Kotake | 4.63001 | 35 | Hisayama | 0 | 35 | Yanagawa | 0 | 35 | Omuta | 59.69015 |
| 36 | Sasaguri | 4.28264 | 36 | Yanagawa | 0 | 36 | Ashiya | 0 | 36 | Nogata | 59.35275 |
| 37 | Okagaki | 4.23642 | 37 | Ashiya | 0 | 37 | Itoshima | 0 | 37 | Chikugo | 58.92988 |
| 38 | Kawasaki | 4.1983 | 38 | Itoshima | 0 | 38 | Itoda | 0 | 38 | Shingu | 58.83266 |
| 39 | Nakagawa | 3.99472 | 39 | Itoda | 0 | 39 | Nakagawa | 0 | 39 | Onojo | 58.7693 |
| 40 | Kitakyushu | 3.86378 | 40 | Nakagawa | 0 | 40 | Kasuya | 0 | 40 | Kurume | 58.67508 |
| 41 | Onga | 3.50627 | 41 | Kasuya | 0 | 41 | Koge | 0 | 41 | Kanda | 58.16945 |
| 42 | Hirokawa | 3.4506 | 42 | Koge | 0 | 42 | Mizumaki | 0 | 42 | Fukuoka | 58.1274 |
| 43 | Chikushino | 3.44772 | 43 | Mizumaki | 0 | 43 | Dazaifu | 0 | 43 | Sasaguri | 58.04412 |
| 44 | Sue | 3.4391 | 44 | Soeda | 0 | 44 | Tagawa | 0 | 44 | Sue | 57.88924 |
| 45 | Chikujo | 3.38016 | 45 | Tagawa | 0 | 45 | Kawara | 0 | 45 | Oto | 57.72021 |
| 46 | Onojo | 3.28136 | 46 | Kawara | 0 | 46 | Ogori | 0 | 46 | Chikushino | 57.37557 |
| 47 | Fukutsu | 2.43667 | 47 | Ogori | 0 | 47 | Kotake | 0 | 47 | Kasuya | 57.23602 |
| 48 | Mizumaki | 2.40218 | 48 | Kotake | 0 | 48 | Sasaguri | 0 | 48 | Shime | 56.96419 |
| 49 | Koga | 2.25672 | 49 | Sasaguri | 0 | 49 | Shingu | 0 | 49 | Kasuga | 56.92052 |
| 50 | Shime | 2.14614 | 50 | Shingu | 0 | 50 | Sue | 0 | 50 | Koga | 56.62051 |
| 51 | Tachiarai | 2.08576 | 51 | Sue | 0 | 51 | Kanda | 0 | 51 | Ukiha | 56.55779 |
| 52 | Kasuga | 2.07161 | 52 | Kanda | 0 | 52 | Umi | 0 | 52 | Nakagawa | 55.39034 |
| 53 | Munakata | 2.05726 | 53 | Umi | 0 | 53 | Onga | 0 | 53 | Itoshima | 55.19569 |
| 54 | Itoshima | 1.96534 | 54 | Onga | 0 | 54 | Shime | 0 | 54 | Oki | 55.13168 |
| 55 | Kasuya | 1.37323 | 55 | Shime | 0 | 55 | Nakama | 0 | 55 | Umi | 53.91166 |
| 56 | Shingu | 1.00138 | 56 | Chikugo | 0 | 56 | Chikugo | 0 | 56 | Mizumaki | 53.7049 |
| 57 | Aka | 0 | 57 | Chikuzen | 0 | 57 | Chikuzen | 0 | 57 | Hisayama | 51.27795 |
| 58 | Hisayama | 0 | 58 | Chikushino | 0 | 58 | Chikushino | 0 | 58 | Okawa | 47.27335 |
| 59 | Itoda | 0 | 59 | Chikujo | 0 | 59 | Chikujo | 0 | 59 | Itoda | 45.2182 |
| 60 | Umi | 0 | 60 | Munakata | 0 | 60 | Munakata | 0 | 60 | Onga | 43.79651 |

| Supplement Table 1C. Calculated score ranking of 34 measurable indicators by municipality (#21-#24) | | | | | | | | | | | |
| --- | --- | --- | --- | --- | --- | --- | --- | --- | --- | --- | --- |
| **#** | **Municipality** | **21.Influenza** | **#** | **Municipality** | **22.COVID-19** | **#** | **Municipality** | **23.Infectious gastroenteritis** | **#** | **Municipality** | **24.Tuberculosis incidence** |
| 1 | Aka | 100 | 1 | Kurume | 100 | 1 | Fukutsu | 100 | 1 | Itoshima | 100 |
| 2 | Kawasaki | 100 | 2 | Aka | 96.86366 | 2 | Okagaki | 100 | 2 | Kurate | 85.6331 |
| 3 | Oto | 100 | 3 | Kawasaki | 96.86366 | 3 | Ashiya | 100 | 3 | Iizuka | 85.6331 |
| 4 | Fukuchi | 100 | 4 | Oto | 96.86366 | 4 | Mizumaki | 100 | 4 | Miyawaka | 85.6331 |
| 5 | Itoda | 100 | 5 | Fukuchi | 96.86366 | 5 | Onga | 100 | 5 | Keisen | 85.6331 |
| 6 | Soeda | 100 | 6 | Itoda | 96.86366 | 6 | Nakama | 100 | 6 | Kama | 85.6331 |
| 7 | Tagawa | 100 | 7 | Soeda | 96.86366 | 7 | Munakata | 100 | 7 | Kotake | 85.6331 |
| 8 | Kawara | 100 | 8 | Tagawa | 96.86366 | 8 | Aka | 97.89752 | 8 | Nogata | 85.6331 |
| 9 | Kurume | 98.10264 | 9 | Kawara | 96.86366 | 9 | Kawasaki | 97.89752 | 9 | Koga | 71.13403 |
| 10 | Ukiha | 91.85381 | 10 | Itoshima | 93.2886 | 10 | Oto | 97.89752 | 10 | Hisayama | 71.13403 |
| 11 | Asakura | 91.85381 | 11 | Miyako | 91.6132 | 11 | Fukuchi | 97.89752 | 11 | Kasuya | 71.13403 |
| 12 | Tachiarai | 91.85381 | 12 | Yukuhashi | 91.6132 | 12 | Itoda | 97.89752 | 12 | Sasaguri | 71.13403 |
| 13 | Toho | 91.85381 | 13 | Yoshitomi | 91.6132 | 13 | Soeda | 97.89752 | 13 | Shingu | 71.13403 |
| 14 | Ogori | 91.85381 | 14 | Buzen | 91.6132 | 14 | Tagawa | 97.89752 | 14 | Sue | 71.13403 |
| 15 | Chikuzen | 91.85381 | 15 | Koge | 91.6132 | 15 | Kawara | 97.89752 | 15 | Umi | 71.13403 |
| 16 | Kurate | 91.54332 | 16 | Kanda | 91.6132 | 16 | Kurate | 92.38868 | 16 | Shime | 71.13403 |
| 17 | Iizuka | 91.54332 | 17 | Chikujo | 91.6132 | 17 | Iizuka | 92.38868 | 17 | Ukiha | 68.41559 |
| 18 | Miyawaka | 91.54332 | 18 | Koga | 90.54184 | 18 | Miyawaka | 92.38868 | 18 | Asakura | 68.41559 |
| 19 | Keisen | 91.54332 | 19 | Hisayama | 90.54184 | 19 | Keisen | 92.38868 | 19 | Tachiarai | 68.41559 |
| 20 | Kama | 91.54332 | 20 | Kasuya | 90.54184 | 20 | Kama | 92.38868 | 20 | Toho | 68.41559 |
| 21 | Kotake | 91.54332 | 21 | Sasaguri | 90.54184 | 21 | Kotake | 92.38868 | 21 | Ogori | 68.41559 |
| 22 | Nogata | 91.54332 | 22 | Shingu | 90.54184 | 22 | Nogata | 92.38868 | 22 | Chikuzen | 68.41559 |
| 23 | Miyako | 91.38048 | 23 | Sue | 90.54184 | 23 | Miyako | 89.92764 | 23 | Kasuga | 65.21915 |
| 24 | Yukuhashi | 91.38048 | 24 | Umi | 90.54184 | 24 | Yukuhashi | 89.92764 | 24 | Onojo | 65.21915 |
| 25 | Yoshitomi | 91.38048 | 25 | Shime | 90.54184 | 25 | Yoshitomi | 89.92764 | 25 | Nakagawa | 65.21915 |
| 26 | Buzen | 91.38048 | 26 | Kitakyushu | 89.28642 | 26 | Buzen | 89.92764 | 26 | Dazaifu | 65.21915 |
| 27 | Koge | 91.38048 | 27 | Kurate | 88.02563 | 27 | Koge | 89.92764 | 27 | Chikushino | 65.21915 |
| 28 | Kanda | 91.38048 | 28 | Iizuka | 88.02563 | 28 | Kanda | 89.92764 | 28 | Fukutsu | 62.482 |
| 29 | Chikujo | 91.38048 | 29 | Miyawaka | 88.02563 | 29 | Chikujo | 89.92764 | 29 | Okagaki | 62.482 |
| 30 | Miyama | 89.42029 | 30 | Keisen | 88.02563 | 30 | Kurume | 89.55766 | 30 | Ashiya | 62.482 |
| 31 | Yame | 89.42029 | 31 | Kama | 88.02563 | 31 | Ukiha | 85.88565 | 31 | Mizumaki | 62.482 |
| 32 | Okawa | 89.42029 | 32 | Kotake | 88.02563 | 32 | Asakura | 85.88565 | 32 | Onga | 62.482 |
| 33 | Omuta | 89.42029 | 33 | Nogata | 88.02563 | 33 | Tachiarai | 85.88565 | 33 | Nakama | 62.482 |
| 34 | Oki | 89.42029 | 34 | Miyama | 86.84347 | 34 | Toho | 85.88565 | 34 | Munakata | 62.482 |
| 35 | Hirokawa | 89.42029 | 35 | Yame | 86.84347 | 35 | Ogori | 85.88565 | 35 | Kurume | 60.93651 |
| 36 | Yanagawa | 89.42029 | 36 | Okawa | 86.84347 | 36 | Chikuzen | 85.88565 | 36 | Miyama | 45.59419 |
| 37 | Chikugo | 89.42029 | 37 | Omuta | 86.84347 | 37 | Kitakyushu | 75.3276 | 37 | Yame | 45.59419 |
| 38 | Koga | 86.77899 | 38 | Oki | 86.84347 | 38 | Koga | 73.97157 | 38 | Okawa | 45.59419 |
| 39 | Hisayama | 86.77899 | 39 | Hirokawa | 86.84347 | 39 | Hisayama | 73.97157 | 39 | Omuta | 45.59419 |
| 40 | Kasuya | 86.77899 | 40 | Yanagawa | 86.84347 | 40 | Kasuya | 73.97157 | 40 | Oki | 45.59419 |
| 41 | Sasaguri | 86.77899 | 41 | Chikugo | 86.84347 | 41 | Sasaguri | 73.97157 | 41 | Hirokawa | 45.59419 |
| 42 | Shingu | 86.77899 | 42 | Fukutsu | 83.17905 | 42 | Shingu | 73.97157 | 42 | Yanagawa | 45.59419 |
| 43 | Sue | 86.77899 | 43 | Okagaki | 83.17905 | 43 | Sue | 73.97157 | 43 | Chikugo | 45.59419 |
| 44 | Umi | 86.77899 | 44 | Ashiya | 83.17905 | 44 | Umi | 73.97157 | 44 | Fukuoka | 44.31198 |
| 45 | Shime | 86.77899 | 45 | Mizumaki | 83.17905 | 45 | Shime | 73.97157 | 45 | Kitakyushu | 26.00394 |
| 46 | Fukutsu | 85.46983 | 46 | Onga | 83.17905 | 46 | Itoshima | 68.72351 | 46 | Miyako | 18.81595 |
| 47 | Okagaki | 85.46983 | 47 | Nakama | 83.17905 | 47 | Miyama | 63.34181 | 47 | Yukuhashi | 18.81595 |
| 48 | Ashiya | 85.46983 | 48 | Munakata | 83.17905 | 48 | Yame | 63.34181 | 48 | Yoshitomi | 18.81595 |
| 49 | Mizumaki | 85.46983 | 49 | Ukiha | 80.77899 | 49 | Okawa | 63.34181 | 49 | Buzen | 18.81595 |
| 50 | Onga | 85.46983 | 50 | Asakura | 80.77899 | 50 | Omuta | 63.34181 | 50 | Koge | 18.81595 |
| 51 | Nakama | 85.46983 | 51 | Tachiarai | 80.77899 | 51 | Oki | 63.34181 | 51 | Kanda | 18.81595 |
| 52 | Munakata | 85.46983 | 52 | Toho | 80.77899 | 52 | Hirokawa | 63.34181 | 52 | Chikujo | 18.81595 |
| 53 | Kitakyushu | 84.80731 | 53 | Ogori | 80.77899 | 53 | Yanagawa | 63.34181 | 53 | Aka | 0 |
| 54 | Itoshima | 84.02283 | 54 | Chikuzen | 80.77899 | 54 | Chikugo | 63.34181 | 54 | Kawasaki | 0 |
| 55 | Kasuga | 80.72272 | 55 | Kasuga | 80.10526 | 55 | Kasuga | 55.34259 | 55 | Oto | 0 |
| 56 | Onojo | 80.72272 | 56 | Onojo | 80.10526 | 56 | Onojo | 55.34259 | 56 | Fukuchi | 0 |
| 57 | Nakagawa | 80.72272 | 57 | Nakagawa | 80.10526 | 57 | Nakagawa | 55.34259 | 57 | Itoda | 0 |
| 58 | Dazaifu | 80.72272 | 58 | Dazaifu | 80.10526 | 58 | Dazaifu | 55.34259 | 58 | Soeda | 0 |
| 59 | Chikushino | 80.72272 | 59 | Chikushino | 80.10526 | 59 | Chikushino | 55.34259 | 59 | Tagawa | 0 |
| 60 | Fukuoka | 0 | 60 | Fukuoka | 0 | 60 | Fukuoka | 0 | 60 | Kawara | 0 |

| Supplement Table 1C. Calculated score ranking of 34 measurable indicators by municipality (#25-#28) | | | | | | | | | | | |
| --- | --- | --- | --- | --- | --- | --- | --- | --- | --- | --- | --- |
| **#** | **Municipality** | **25.Food waste per capita** | **#** | **Municipality** | **26.Good Agricultural Practices (GAP) certified agricultural producers** | **#** | **Municipality** | **27.Food chain inspection implementation rate** | **#** | **Municipality** | **28.Arable land per capita** |
| 1 | Aka | 100 | 1 | Oto | 100 | 1 | Okagaki | 100 | 1 | Toho | 100 |
| 2 | Toho | 100 | 2 | Onga | 82.01881 | 2 | Nogata | 100 | 2 | Aka | 90.28479 |
| 3 | Koge | 99.90902 | 3 | Keisen | 81.10678 | 3 | Shime | 100 | 3 | Koge | 89.27174 |
| 4 | Kotake | 99.8341 | 4 | Oki | 74.89564 | 4 | Nakama | 100 | 4 | Miyama | 79.88336 |
| 5 | Miyako | 99.75784 | 5 | Asakura | 40.99513 | 5 | Chikugo | 100 | 5 | Miyako | 77.58078 |
| 6 | Ashiya | 99.72305 | 6 | Kurate | 34.49579 | 6 | Chikuzen | 100 | 6 | Yame | 75.0803 |
| 7 | Chikujo | 99.71235 | 7 | Tagawa | 33.78425 | 7 | Chikushino | 100 | 7 | Asakura | 68.29775 |
| 8 | Soeda | 99.62271 | 8 | Fukuchi | 24.28585 | 8 | Chikujo | 100 | 8 | Chikujo | 67.6625 |
| 9 | Itoda | 99.5906 | 9 | Yukuhashi | 21.44742 | 9 | Onojo | 95.32049 | 9 | Ukiha | 65.07773 |
| 10 | Kama | 99.36449 | 10 | Buzen | 19.24685 | 10 | Toho | 95.32049 | 10 | Chikuzen | 55.23624 |
| 11 | Okawa | 99.34576 | 11 | Ukiha | 18.47328 | 11 | Iizuka | 95.32049 | 11 | Tachiarai | 53.71454 |
| 12 | Umi | 99.27351 | 12 | Chikuzen | 17.22106 | 12 | Fukuoka | 95.32049 | 12 | Oki | 49.92248 |
| 13 | Kawara | 99.19725 | 13 | Kurume | 17.2066 | 13 | Fukutsu | 95.32049 | 13 | Miyawaka | 45.68243 |
| 14 | Kurate | 99.16113 | 14 | Yame | 17.20512 | 14 | Fukuchi | 95.32049 | 14 | Buzen | 44.36736 |
| 15 | Ogori | 99.12366 | 15 | Iizuka | 16.67628 | 15 | Kawasaki | 86.27098 | 15 | Yanagawa | 43.15303 |
| 16 | Okagaki | 99.10761 | 16 | Itoshima | 15.32443 | 16 | Kasuga | 86.27098 | 16 | Soeda | 40.62692 |
| 17 | Yanagawa | 99.0474 | 17 | Dazaifu | 14.54978 | 17 | Oto | 86.27098 | 17 | Oto | 40.3616 |
| 18 | Oki | 99.02198 | 18 | Fukutsu | 7.59984 | 18 | Okawa | 86.27098 | 18 | Kurate | 38.98904 |
| 19 | Kasuga | 98.97114 | 19 | Fukuoka | 5.64005 | 19 | Tachiarai | 86.27098 | 19 | Kama | 37.70625 |
| 20 | Yoshitomi | 98.96579 | 20 | Kitakyushu | 0.56313 | 20 | Omuta | 86.27098 | 20 | Kawara | 31.70798 |
| 21 | Yukuhashi | 98.89087 | 21 | Miyako | 0 | 21 | Oki | 86.27098 | 21 | Itoda | 29.14437 |
| 22 | Kanda | 98.79855 | 22 | Miyama | 0 | 22 | Munakata | 66.5258 | 22 | Okawa | 28.47016 |
| 23 | Mizumaki | 98.76644 | 23 | Aka | 0 | 23 | Miyawaka | 38.34361 | 23 | Chikugo | 27.88295 |
| 24 | Buzen | 98.55772 | 24 | Kawasaki | 0 | 24 | Aka | 33.46398 | 24 | Fukuchi | 26.57534 |
| 25 | Nakagawa | 98.52026 | 25 | Kasuga | 0 | 25 | Mizumaki | 33.46398 | 25 | Hirokawa | 25.40755 |
| 26 | Onga | 98.34098 | 26 | Okawa | 0 | 26 | Dazaifu | 33.46398 | 26 | Onga | 25.07148 |
| 27 | Tachiarai | 98.29817 | 27 | Tachiarai | 0 | 27 | Soeda | 33.46398 | 27 | Keisen | 23.88464 |
| 28 | Omuta | 98.23127 | 28 | Omuta | 0 | 28 | Tagawa | 33.46398 | 28 | Ogori | 21.77764 |
| 29 | Sasaguri | 98.16705 | 29 | Onojo | 0 | 29 | Kawara | 33.46398 | 29 | Kurume | 19.56341 |
| 30 | Nakama | 97.79511 | 30 | Toho | 0 | 30 | Ogori | 33.46398 | 30 | Yoshitomi | 19.21412 |
| 31 | Shime | 97.78173 | 31 | Okagaki | 0 | 31 | Kotake | 33.46398 | 31 | Yukuhashi | 19.12807 |
| 32 | Fukuchi | 97.67737 | 32 | Miyawaka | 0 | 32 | Sasaguri | 33.46398 | 32 | Kawasaki | 17.90829 |
| 33 | Nogata | 97.64393 | 33 | Koga | 0 | 33 | Miyako | 27.30317 | 33 | Hisayama | 16.72318 |
| 34 | Onojo | 97.45662 | 34 | Hirokawa | 0 | 34 | Miyama | 27.30317 | 34 | Kotake | 14.7749 |
| 35 | Miyama | 96.99637 | 35 | Yoshitomi | 0 | 35 | Kurate | 27.30317 | 35 | Munakata | 14.48935 |
| 36 | Dazaifu | 96.92814 | 36 | Kama | 0 | 36 | Yame | 27.30317 | 36 | Iizuka | 13.31917 |
| 37 | Sue | 96.45586 | 37 | Hisayama | 0 | 37 | Kitakyushu | 27.30317 | 37 | Itoshima | 12.66844 |
| 38 | Iizuka | 96.34347 | 38 | Yanagawa | 0 | 38 | Asakura | 27.30317 | 38 | Okagaki | 12.39901 |
| 39 | Kitakyushu | 94.95739 | 39 | Ashiya | 0 | 39 | Shingu | 25.41505 | 39 | Fukutsu | 11.60695 |
| 40 | Chikushino | 94.24963 | 40 | Itoda | 0 | 40 | Sue | 25.41505 | 40 | Tagawa | 10.04139 |
| 41 | Munakata | 94.16534 | 41 | Nakagawa | 0 | 41 | Kanda | 25.41505 | 41 | Kanda | 9.90514 |
| 42 | Fukutsu | 93.89107 | 42 | Kasuya | 0 | 42 | Umi | 25.41505 | 42 | Nogata | 8.12795 |
| 43 | Yame | 92.8689 | 43 | Koge | 0 | 43 | Onga | 25.41505 | 43 | Omuta | 6.69945 |
| 44 | Chikuzen | 91.91764 | 44 | Mizumaki | 0 | 44 | Buzen | 21.90986 | 44 | Chikushino | 5.70082 |
| 45 | Oto | 90.72689 | 45 | Soeda | 0 | 45 | Yanagawa | 21.90986 | 45 | Koga | 5.22464 |
| 46 | Kasuya | 89.60572 | 46 | Kawara | 0 | 46 | Ashiya | 21.90986 | 46 | Nakagawa | 5.18294 |
| 47 | Kawasaki | 89.05985 | 47 | Ogori | 0 | 47 | Itoshima | 21.90986 | 47 | Nakama | 4.67235 |
| 48 | Chikugo | 88.68924 | 48 | Kotake | 0 | 48 | Itoda | 21.90986 | 48 | Sasaguri | 4.63016 |
| 49 | Hirokawa | 88.31998 | 49 | Sasaguri | 0 | 49 | Nakagawa | 21.90986 | 49 | Ashiya | 4.3965 |
| 50 | Itoshima | 86.2743 | 50 | Shingu | 0 | 50 | Kasuya | 21.90986 | 50 | Kasuya | 3.47402 |
| 51 | Keisen | 84.91096 | 51 | Sue | 0 | 51 | Koge | 21.90986 | 51 | Shingu | 3.38085 |
| 52 | Koga | 81.70799 | 52 | Kanda | 0 | 52 | Ukiha | 14.64637 | 52 | Sue | 3.20639 |
| 53 | Shingu | 78.34847 | 53 | Umi | 0 | 53 | Koga | 0 | 53 | Mizumaki | 2.57177 |
| 54 | Kurume | 74.21832 | 54 | Nogata | 0 | 54 | Hirokawa | 0 | 54 | Umi | 1.6164 |
| 55 | Hisayama | 73.81293 | 55 | Shime | 0 | 55 | Keisen | 0 | 55 | Kitakyushu | 1.50388 |
| 56 | Fukuoka | 73.75139 | 56 | Nakama | 0 | 56 | Yukuhashi | 0 | 56 | Dazaifu | 1.17882 |
| 57 | Miyawaka | 70.20724 | 57 | Chikugo | 0 | 57 | Yoshitomi | 0 | 57 | Shime | 0.75548 |
| 58 | Tagawa | 53.27857 | 58 | Chikushino | 0 | 58 | Kama | 0 | 58 | Fukuoka | 0.65381 |
| 59 | Ukiha | 44.97545 | 59 | Chikujo | 0 | 59 | Kurume | 0 | 59 | Onojo | 0.21188 |
| 60 | Asakura | 0 | 60 | Munakata | 0 | 60 | Hisayama | 0 | 60 | Kasuga | 0 |

| Supplement Table 1C. Calculated score ranking of 34 measurable indicators by municipality (#29-#32) | | | | | | | | | | | |
| --- | --- | --- | --- | --- | --- | --- | --- | --- | --- | --- | --- |
| **#** | **Municipality** | **29.Local food promotion restaurants** | **#** | **Municipality** | **30.AMR surveillance system** | **#** | **Municipality** | **31.Japan Nosocomial Infections Surveillance (JANIS) participating medical facilities** | **#** | **Municipality** | **32.CO2 emissions (kg/GDP)** |
| 1 | Aka | 100 | 1 | Ukiha | 100 | 1 | Itoda | 100 | 1 | Itoshima | 100 |
| 2 | Toho | 63.76132 | 2 | Miyako | 100 | 2 | Hisayama | 94.37474 | 2 | Fukuoka | 91.2884 |
| 3 | Onga | 62.88687 | 3 | Miyama | 100 | 3 | Kama | 75.2069 | 3 | Kasuga | 88.11005 |
| 4 | Itoshima | 47.58672 | 4 | Kurate | 100 | 4 | Kanda | 69.96739 | 4 | Ashiya | 88.06526 |
| 5 | Chikujo | 39.40631 | 5 | Yame | 100 | 5 | Ashiya | 67.04581 | 5 | Chikushino | 86.98056 |
| 6 | Munakata | 37.513 | 6 | Kitakyushu | 100 | 6 | Sue | 59.95053 | 6 | Dazaifu | 85.93872 |
| 7 | Chikuzen | 35.65088 | 7 | Asakura | 100 | 7 | Fukuoka | 59.52234 | 7 | Shime | 85.47777 |
| 8 | Buzen | 33.20386 | 8 | Aka | 100 | 8 | Ogori | 58.39358 | 8 | Toho | 85.23992 |
| 9 | Ashiya | 32.19182 | 9 | Kawasaki | 100 | 9 | Kurate | 57.83996 | 9 | Kasuya | 85.23479 |
| 10 | Koge | 30.14989 | 10 | Kasuga | 100 | 10 | Yame | 57.69651 | 10 | Ogori | 85.18346 |
| 11 | Fukutsu | 29.71809 | 11 | Oto | 100 | 11 | Okagaki | 55.38696 | 11 | Sasaguri | 84.24738 |
| 12 | Miyama | 26.5712 | 12 | Okawa | 100 | 12 | Omuta | 55.38244 | 12 | Fukutsu | 84.18492 |
| 13 | Kurume | 25.72623 | 13 | Tachiarai | 100 | 13 | Kawasaki | 54.88864 | 13 | Onojo | 83.95788 |
| 14 | Keisen | 23.32034 | 14 | Omuta | 100 | 14 | Yanagawa | 54.55142 | 14 | Chikujo | 83.70257 |
| 15 | Okagaki | 22.79474 | 15 | Oki | 100 | 15 | Okawa | 53.94009 | 15 | Munakata | 83.49624 |
| 16 | Yame | 22.75586 | 16 | Onojo | 100 | 16 | Munakata | 53.79319 | 16 | Kurume | 83.32956 |
| 17 | Oki | 21.53448 | 17 | Toho | 100 | 17 | Nakagawa | 52.22701 | 17 | Okagaki | 82.31272 |
| 18 | Yanagawa | 20.57997 | 18 | Iizuka | 100 | 18 | Onga | 45.841 | 18 | Yanagawa | 82.23282 |
| 19 | Okawa | 20.34934 | 19 | Fukuoka | 100 | 19 | Koga | 44.25656 | 19 | Nakagawa | 81.4371 |
| 20 | Fukuoka | 20.1468 | 20 | Fukutsu | 100 | 20 | Nakama | 43.24378 | 20 | Umi | 80.85002 |
| 21 | Soeda | 19.84524 | 21 | Fukuchi | 100 | 21 | Kitakyushu | 42.48941 | 21 | Iizuka | 80.70229 |
| 22 | Shingu | 17.96026 | 22 | Okagaki | 100 | 22 | Chikushino | 40.97776 | 22 | Okawa | 80.63608 |
| 23 | Iizuka | 17.74103 | 23 | Miyawaka | 100 | 23 | Tagawa | 37.7646 | 23 | Mizumaki | 80.47706 |
| 24 | Yoshitomi | 17.1265 | 24 | Koga | 100 | 24 | Kurume | 37.50595 | 24 | Onga | 80.17287 |
| 25 | Miyako | 16.96706 | 25 | Hirokawa | 100 | 25 | Shime | 37.41155 | 25 | Omuta | 79.86984 |
| 26 | Tagawa | 16.83737 | 26 | Keisen | 100 | 26 | Kasuya | 35.90715 | 26 | Kitakyushu | 79.85151 |
| 27 | Asakura | 16.50206 | 27 | Yukuhashi | 100 | 27 | Iizuka | 34.95192 | 27 | Yame | 79.51678 |
| 28 | Nogata | 16.04548 | 28 | Yoshitomi | 100 | 28 | Onojo | 34.32045 | 28 | Shingu | 79.19016 |
| 29 | Kama | 15.47586 | 29 | Kama | 100 | 29 | Miyawaka | 32.8365 | 29 | Tagawa | 79.12808 |
| 30 | Hirokawa | 15.47213 | 30 | Kurume | 100 | 30 | Mizumaki | 31.40615 | 30 | Sue | 79.07535 |
| 31 | Kawasaki | 15.05977 | 31 | Hisayama | 100 | 31 | Nogata | 31.19001 | 31 | Yukuhashi | 78.98912 |
| 32 | Ogori | 15.02008 | 32 | Buzen | 100 | 32 | Kasuga | 30.95346 | 32 | Hisayama | 78.8779 |
| 33 | Kitakyushu | 13.27694 | 33 | Yanagawa | 100 | 33 | Fukutsu | 25.48567 | 33 | Kawara | 78.48281 |
| 34 | Koga | 13.15454 | 34 | Ashiya | 100 | 34 | Dazaifu | 24.39597 | 34 | Miyama | 78.35203 |
| 35 | Chikugo | 12.14212 | 35 | Itoshima | 100 | 35 | Miyama | 24.21114 | 35 | Asakura | 78.08738 |
| 36 | Nakagawa | 10.74713 | 36 | Itoda | 100 | 36 | Yukuhashi | 23.97428 | 36 | Soeda | 77.99716 |
| 37 | Yukuhashi | 10.68894 | 37 | Nakagawa | 100 | 37 | Asakura | 17.18439 | 37 | Nakama | 77.203 |
| 38 | Omuta | 10.31107 | 38 | Kasuya | 100 | 38 | Itoshima | 17.1299 | 38 | Chikuzen | 77.09318 |
| 39 | Chikushino | 8.99444 | 39 | Koge | 100 | 39 | Ukiha | 0 | 39 | Koga | 76.3814 |
| 40 | Dazaifu | 8.36689 | 40 | Mizumaki | 100 | 40 | Miyako | 0 | 40 | Nogata | 76.22842 |
| 41 | Kotake | 8.30419 | 41 | Dazaifu | 100 | 41 | Aka | 0 | 41 | Keisen | 75.54176 |
| 42 | Kurate | 7.93476 | 42 | Soeda | 100 | 42 | Oto | 0 | 42 | Oto | 75.48035 |
| 43 | Tachiarai | 7.48187 | 43 | Tagawa | 100 | 43 | Tachiarai | 0 | 43 | Tachiarai | 75.45913 |
| 44 | Itoda | 6.85924 | 44 | Kawara | 100 | 44 | Oki | 0 | 44 | Oki | 75.00821 |
| 45 | Miyawaka | 6.757 | 45 | Ogori | 100 | 45 | Toho | 0 | 45 | Hirokawa | 74.9634 |
| 46 | Mizumaki | 6.46267 | 46 | Kotake | 100 | 46 | Fukuchi | 0 | 46 | Ukiha | 74.89445 |
| 47 | Nakama | 5.93239 | 47 | Sasaguri | 100 | 47 | Hirokawa | 0 | 47 | Kama | 74.74994 |
| 48 | Kawara | 5.6597 | 48 | Shingu | 100 | 48 | Keisen | 0 | 48 | Chikugo | 72.76827 |
| 49 | Fukuchi | 5.58626 | 49 | Sue | 100 | 49 | Yoshitomi | 0 | 49 | Kawasaki | 72.26062 |
| 50 | Kanda | 4.79923 | 50 | Kanda | 100 | 50 | Buzen | 0 | 50 | Fukuchi | 72.00472 |
| 51 | Sue | 4.11215 | 51 | Umi | 100 | 51 | Koge | 0 | 51 | Aka | 71.32742 |
| 52 | Shime | 3.84922 | 52 | Onga | 100 | 52 | Soeda | 0 | 52 | Itoda | 70.9044 |
| 53 | Kasuya | 3.69444 | 53 | Nogata | 100 | 53 | Kawara | 0 | 53 | Kotake | 69.98864 |
| 54 | Umi | 3.21859 | 54 | Shime | 100 | 54 | Kotake | 0 | 54 | Kurate | 68.58615 |
| 55 | Kasuga | 3.18476 | 55 | Nakama | 100 | 55 | Sasaguri | 0 | 55 | Buzen | 68.04846 |
| 56 | Onojo | 2.94265 | 56 | Chikugo | 100 | 56 | Shingu | 0 | 56 | Miyako | 67.40422 |
| 57 | Ukiha | 2.12462 | 57 | Chikuzen | 100 | 57 | Umi | 0 | 57 | Yoshitomi | 67.26485 |
| 58 | Sasaguri | 1.92029 | 58 | Chikushino | 100 | 58 | Chikugo | 0 | 58 | Koge | 66.549 |
| 59 | Oto | 0 | 59 | Chikujo | 100 | 59 | Chikuzen | 0 | 59 | Kanda | 14.16584 |
| 60 | Hisayama | 0 | 60 | Munakata | 100 | 60 | Chikujo | 0 | 60 | Miyawaka | 0 |

| Supplement Table 1C. Calculated score ranking of 34 measurable indicators by municipality (#33-#34) | | | | | |
| --- | --- | --- | --- | --- | --- |
| **#** | **Municipality** | **33.CO2 emissions (tonnes/capita)** | **#** | **Municipality** | **34.Days with WBGT index above 25** |
| 1 | Itoshima | 100 | 1 | Kitakyushu | 100 |
| 2 | Kasuga | 96.67484 | 2 | Yukuhashi | 100 |
| 3 | Dazaifu | 95.70192 | 3 | Munakata | 93.33333 |
| 4 | Fukutsu | 95.69822 | 4 | Soeda | 86.66667 |
| 5 | Chikushino | 95.6782 | 5 | Yame | 80 |
| 6 | Itoda | 95.55244 | 6 | Iizuka | 73.33333 |
| 7 | Okagaki | 95.38317 | 7 | Fukuoka | 60 |
| 8 | Nakagawa | 95.3039 | 8 | Ukiha | 52.77776 |
| 9 | Munakata | 95.26126 | 9 | Miyako | 52.77776 |
| 10 | Ogori | 94.68354 | 10 | Miyama | 52.77776 |
| 11 | Ashiya | 94.66799 | 11 | Kurate | 52.77776 |
| 12 | Shime | 94.61407 | 12 | Aka | 52.77776 |
| 13 | Soeda | 94.55711 | 13 | Kawasaki | 52.77776 |
| 14 | Onojo | 94.5563 | 14 | Kasuga | 52.77776 |
| 15 | Fukuoka | 94.26685 | 15 | Oto | 52.77776 |
| 16 | Sasaguri | 94.14218 | 16 | Okawa | 52.77776 |
| 17 | Mizumaki | 94.12021 | 17 | Tachiarai | 52.77776 |
| 18 | Kawasaki | 93.97362 | 18 | Oki | 52.77776 |
| 19 | Chikujo | 93.76605 | 19 | Onojo | 52.77776 |
| 20 | Aka | 93.63507 | 20 | Toho | 52.77776 |
| 21 | Yanagawa | 93.50724 | 21 | Fukutsu | 52.77776 |
| 22 | Fukuchi | 93.26953 | 22 | Fukuchi | 52.77776 |
| 23 | Nakama | 93.25357 | 23 | Okagaki | 52.77776 |
| 24 | Kawara | 93.22758 | 24 | Miyawaka | 52.77776 |
| 25 | Oto | 93.15158 | 25 | Koga | 52.77776 |
| 26 | Kasuya | 93.12837 | 26 | Hirokawa | 52.77776 |
| 27 | Keisen | 93.05502 | 27 | Keisen | 52.77776 |
| 28 | Chikuzen | 92.93934 | 28 | Yoshitomi | 52.77776 |
| 29 | Miyama | 92.91696 | 29 | Kama | 52.77776 |
| 30 | Umi | 92.75755 | 30 | Hisayama | 52.77776 |
| 31 | Kurume | 92.41447 | 31 | Buzen | 52.77776 |
| 32 | Onga | 92.28314 | 32 | Yanagawa | 52.77776 |
| 33 | Yukuhashi | 92.17091 | 33 | Ashiya | 52.77776 |
| 34 | Kama | 92.00295 | 34 | Itoda | 52.77776 |
| 35 | Tachiarai | 91.94818 | 35 | Nakagawa | 52.77776 |
| 36 | Oki | 91.81997 | 36 | Kasuya | 52.77776 |
| 37 | Sue | 91.60035 | 37 | Koge | 52.77776 |
| 38 | Iizuka | 91.4496 | 38 | Mizumaki | 52.77776 |
| 39 | Tagawa | 91.3899 | 39 | Tagawa | 52.77776 |
| 40 | Okawa | 91.20986 | 40 | Kawara | 52.77776 |
| 41 | Toho | 91.12326 | 41 | Ogori | 52.77776 |
| 42 | Yame | 90.54604 | 42 | Kotake | 52.77776 |
| 43 | Ukiha | 90.04213 | 43 | Sasaguri | 52.77776 |
| 44 | Omuta | 89.39225 | 44 | Shingu | 52.77776 |
| 45 | Shingu | 89.13873 | 45 | Sue | 52.77776 |
| 46 | Kitakyushu | 88.81764 | 46 | Kanda | 52.77776 |
| 47 | Nogata | 88.71558 | 47 | Umi | 52.77776 |
| 48 | Miyako | 87.80661 | 48 | Onga | 52.77776 |
| 49 | Koga | 87.27295 | 49 | Nogata | 52.77776 |
| 50 | Chikugo | 86.63264 | 50 | Shime | 52.77776 |
| 51 | Kotake | 86.26496 | 51 | Nakama | 52.77776 |
| 52 | Hirokawa | 86.16401 | 52 | Chikugo | 52.77776 |
| 53 | Koge | 86.06794 | 53 | Chikuzen | 52.77776 |
| 54 | Buzen | 85.91323 | 54 | Chikushino | 52.77776 |
| 55 | Yoshitomi | 85.18412 | 55 | Chikujo | 52.77776 |
| 56 | Asakura | 79.84076 | 56 | Asakura | 26.66667 |
| 57 | Kurate | 79.74614 | 57 | Omuta | 20 |
| 58 | Hisayama | 79.41488 | 58 | Itoshima | 20 |
| 59 | Kanda | 23.11829 | 59 | Dazaifu | 20 |
| 60 | Miyawaka | 0 | 60 | Kurume | 0 |

| Supplementary Table 2A: Summary Statistics for FOHI 3 index Scores | | | | | |
| --- | --- | --- | --- | --- | --- |
|  | **Mean** | **SD** | **Min** | **Median** | **Max** |
| External Drivers Index (EDI) | 50.43 | 7.35 | 29.21 | 51.03 | 66.13 |
| Intrinsic Drivers Index (IDI) | 59.17 | 8.79 | 31.10 | 59.64 | 78.07 |
| Core Drivers Index (CDI) | 47.11 | 6.18 | 37.49 | 46.32 | 71.89 |
| Total Score | 52.27 | 4.29 | 41.01 | 52.33 | 63.71 |

| Supplementary table 2B: Summary Statistics for FOHI 13 Key Indicator Scores | | | | | |
| --- | --- | --- | --- | --- | --- |
|  | **Mean** | **SD** | **Min** | **Median** | **Max** |
| Earth Systems | 48.73 | 17.43 | 3.03 | 48.57 | 82.09 |
| Institutional Systems | 61.78 | 12.34 | 5.80 | 60.51 | 100.00 |
| Economic Systems | 71.92 | 25.02 | 0.00 | 80.00 | 100.00 |
| Social Systems | 54.81 | 20.66 | 0.00 | 55.20 | 100.00 |
| Technological Systems | 15.13 | 19.89 | 0.00 | 7.43 | 100.00 |
| Human Health | 49.82 | 13.93 | 3.03 | 50.05 | 80.55 |
| Animal Health and Ecosystem Diversity | 100.00 | 0.00 | 100.00 | 100.00 | 100.00 |
| Environmental Resources | 29.15 | 26.08 | 0.00 | 19.73 | 100.00 |
| One Health Governance | 11.20 | 15.65 | 0.00 | 6.36 | 97.88 |
| Zoonotic Diseases | 68.35 | 14.09 | 22.16 | 72.33 | 91.01 |
| Food Security | 47.83 | 11.20 | 25.52 | 48.38 | 76.51 |
| Antimicrobial Resistance (AMR) | 64.78 | 13.61 | 50.00 | 65.65 | 100.00 |
| Climate Change | 68.14 | 10.74 | 26.39 | 68.95 | 92.79 |

| Supplementary table 2C: Summary Statistics for FOHI 34 Measurement indicators Scores | | | | | |
| --- | --- | --- | --- | --- | --- |
|  | **Mean** | **SD** | **Min** | **Median** | **Max** |
| Forest area (% of total land area) | 41.83 | 28.02 | 0.00 | 40.89 | 100.00 |
| Biochemical Oxygen Demand level (mg/L) | 55.62 | 17.54 | 0.00 | 55.62 | 100.00 |
| Government revenue (% of GDP) | 96.67 | 18.10 | 0.00 | 100.00 | 100.00 |
| Municipal regulations for pet evacuation during disasters (Yes=1; No=0) | 26.88 | 21.74 | 0.00 | 21.74 | 100.00 |
| Unemployment (% of total labor force) | 71.92 | 25.02 | 0.00 | 80.00 | 100.00 |
| Natural population growth rate (%) | 54.81 | 20.66 | 0.00 | 55.20 | 100.00 |
| Renewable electricity generation (% of total electricity demand) | 15.13 | 19.89 | 0.00 | 7.43 | 100.00 |
| Number of hospital and clinic beds (per 1,000 population) | 32.64 | 19.30 | 0.00 | 31.67 | 100.00 |
| Number of doctors (per 1,000 population) | 25.63 | 24.07 | 0.00 | 18.54 | 100.00 |
| Suicide rate (per 10,000 population) | 70.50 | 20.61 | 0.00 | 72.34 | 100.00 |
| Existence of livestock disease surveillance system (Yes=1; No=0) | 100.00 | 0.00 | 100.00 | 100.00 | 100.00 |
| Existence of Wild animal disease surveillance system (Yes=1; No=0) | 100.00 | 0.00 | 100.00 | 100.00 | 100.00 |
| Recycling rate (%) | 29.15 | 26.08 | 0.00 | 19.73 | 100.00 |
| One Health policy agreement in municipal council (Yes=1; No=0) | 20.00 | 40.34 | 0.00 | 0.00 | 100.00 |
| One Health declaration (Yes=1; No=0) | 55.00 | 50.17 | 0.00 | 100.00 | 100.00 |
| Number of registered agricultural, forestry, fishery products (per 10,000 population) | 6.12 | 17.83 | 0.00 | 0.11 | 100.00 |
| Number of Registered One Health declaration businesses implementing One Health Practice (per 10,000 population) | 9.61 | 14.29 | 0.00 | 5.55 | 100.00 |
| OneHealthwebsitepresencescale(3=dedicatedwebsite;2=dedicatedwebpage;1=sectionongovernmentwebsite;0=nowebpresence) | 11.11 | 22.69 | 0.00 | 0.00 | 100.00 |
| Number of One Health promotion and/or education facilities | 4.17 | 16.70 | 0.00 | 0.00 | 100.00 |
| Influenza vaccination rate for age 65+ (%) | 59.53 | 4.65 | 43.80 | 60.38 | 67.21 |
| Influenza Weekly Average Number of Cases per Sentinel Surveillance Site | 88.50 | 12.76 | 0.00 | 89.42 | 100.00 |
| COVID-19 Weekly Average Number of Cases per Sentinel Surveillance Site | 86.69 | 12.61 | 0.00 | 88.03 | 100.00 |
| Infectious Gastroenteritis Weekly Average Number of Cases per Sentinel Surveillance Site | 81.39 | 18.14 | 0.00 | 87.72 | 100.00 |
| Tuberculosis incidence (per 100,000 population) | 51.17 | 28.18 | 0.00 | 62.48 | 100.00 |
| Food waste per capita (kg) | 91.34 | 16.35 | 0.00 | 97.79 | 100.00 |
| Good Agricultural Practices (GAP) certified agricultural producers (per 10,000 population) | 10.71 | 22.36 | 0.00 | 0.00 | 100.00 |
| Food chain inspection implementation rate (%) | 47.71 | 36.30 | 0.00 | 33.46 | 100.00 |
| Arable land per capita (ha) | 27.63 | 26.73 | 0.00 | 19.17 | 100.00 |
| Local food promotion restaurants (per 10,000 population) | 18.24 | 17.47 | 0.00 | 15.27 | 100.00 |
| AMR surveillance system (Yes=1; No=0) | 100.00 | 0.00 | 100.00 | 100.00 | 100.00 |
| Japan Nosocomial Infections Surveillance (JANIS) hospitals (per 10,000 population) | 29.56 | 27.22 | 0.00 | 31.30 | 100.00 |
| CO2emissions(tonnes/capita) | 76.58 | 14.51 | 0.00 | 79.03 | 100.00 |
| CO2emissions(kg/GDP) | 88.88 | 15.20 | 0.00 | 92.59 | 100.00 |
| DayswithWBGTindexabove25(days/year) | 53.56 | 16.01 | 0.00 | 52.78 | 100.00 |

| Supplementary table 3: Raw data for FOHI | | | | | |
| --- | --- | --- | --- | --- | --- |
| **Municipality** | **1.Forest area (% of total land area)** | **2.Biochemical Oxygen Demand level (mg/L)** | **3.Government revenue (% of GDP)** | **4.Municipal regulations for pet evacuation during disasters (Yes=1; No=0)** | **5.Unemployment (% of total labor force)** |
| Miyama | 18.60089345 | 2 | 29 | 1 | 4 |
| Miyako | 63.53781068 | Missing | 23 | 1 | 5.1 |
| Ukiha | 50.47675805 | 0.9 | 22 | 1 | 4.1 |
| Munakata | 41.78756045 | Missing | 20 | 1 | 3.9 |
| Chikushino | 49.3901744 | Missing | 15 | 1 | 4.5 |
| Chikuzen | 33.63636364 | Missing | 20 | 1 | 4.1 |
| Chikugo | 0.16754428 | 0.7 | 13 | 1 | 3.5 |
| Chikujo | 61.13624937 | Missing | 24 | 1 | 4.4 |
| Nakama | 5.07518797 | 4.7 | 24 | 1 | 6.7 |
| Shime | 2.876869965 | Missing | 14 | 1 | 4 |
| Nogata | 34.19689119 | 3.1 | 16 | 1 | 4.9 |
| Onga | 16.70428894 | 3.15 | 18 | 1 | 4.4 |
| Umi | 59.91393578 | Missing | 14 | 1 | 4.1 |
| Kanda | 34.30818879 | Missing | 5 | 1 | 3.7 |
| Sue | 38.44267321 | Missing | 13 | 0 | 4.3 |
| Yukuhashi | 18.42706252 | Missing | 17 | 1 | 4.3 |
| Shingu | 25.99049128 | Missing | 15 | 1 | 3.1 |
| Sasaguri | 67.01772412 | Missing | 15 | 1 | 5.2 |
| Kotake | 25.56022409 | Missing | 23 | 1 | 7.6 |
| Ogori | 2.636783125 | Missing | 15 | 1 | 4.1 |
| Kawara | 65.50561798 | Missing | 30 | 1 | 8.3 |
| Tagawa | 29.20256645 | 4.3 | 25 | 1 | 6.3 |
| Soeda | 83.75945537 | Missing | 49 | 1 | 6.4 |
| Dazaifu | 39.72972973 | Missing | 18 | 1 | 4.9 |
| Mizumaki | 9.627611262 | 4.55 | 20 | 1 | 9.4 |
| Koge | 62.13141026 | 1.1 | 29 | 1 | 4.3 |
| Kasuya | 5.095541401 | Missing | 12 | 1 | 3.7 |
| Nakagawa | 3.000400053 | Missing | 24 | 1 | 4.5 |
| Itoda | 27.98507463 | 4.3 | 74 | 1 | 7.7 |
| Itoshima | 45.38711173 | Missing | 20 | 1 | 4.6 |
| Ashiya | 20.0862069 | 2.0 | 23 | 1 | 5 |
| Yanagawa | 0 | 1.5 | 22 | 1 | 4.8 |
| Buzen | 61.81523792 | Missing | 15 | 1 | 4.5 |
| Hisayama | 68.21581197 | Missing | 10 | 1 | 3.1 |
| Kurume | 15.58531919 | 2.225 | 15 | 1 | 4.1 |
| Kama | 57.87721893 | Missing | 34 | 1 | 6.5 |
| Yoshitomi | 0 | Missing | 18 | 1 | 4.6 |
| Keisen | 31.78873941 | Missing | 26 | 1 | 5.6 |
| Hirokawa | 35.52978387 | Missing | 12 | 1 | 3.4 |
| Koga | 32.70739244 | Missing | 11 | 1 | 3.8 |
| Miyawaka | 59.58282734 | 1.45 | 11 | 1 | 4.6 |
| Okagaki | 55.83881579 | Missing | 20 | 1 | 4.5 |
| Fukuchi | 39.01569187 | 2.7 | 54 | 1 | 9.6 |
| Fukutsu | 25.73919636 | Missing | 21 | 1 | 3.8 |
| Fukuoka | 32.01246142 | Missing | 16 | 1 | 4.7 |
| Iizuka | 50.31293788 | 2.4 | 21 | 1 | 5.1 |
| Toho | 83.52895901 | Missing | 54 | 1 | 3.2 |
| Onojo | 37.72321429 | Missing | 17 | 1 | 4 |
| Okawa | 0 | Missing | 22 | 1 | 3.2 |
| Oki | 23.10620012 | Missing | 15 | 1 | 5.1 |
| Omuta | 0.043782837 | Missing | 23 | 1 | 3.3 |
| Tachiarai | 0 | Missing | 18 | 1 | 4.4 |
| Oto | 30.99579243 | 1 | 73 | 0 | 9 |
| Kasuga | 4.45229682 | Missing | 18 | 1 | 4.4 |
| Kawasaki | 48.53348091 | Missing | 45 | 1 | 9.2 |
| Aka | 71.91994997 | Missing | 73 | 1 | 4 |
| Asakura | 54.64310324 | Missing | 12 | 1 | 3.7 |
| Kitakyushu | 39.18781726 | Missing | 17 | 1 | 4.4 |
| Yame | 65.31382141 | Missing | 22 | 1 | 3.4 |
| Kurate | 35.2247191 | Missing | 19 | 1 | 6.4 |

| Supplementary table 3: Raw data for FOHI | | | | | |
| --- | --- | --- | --- | --- | --- |
| **Municipality** | **6.Natural population growth rate (%)** | **7.Renewable electricity generation (% of total electricity demand)** | **8.Number of hospital and clinic beds (per 1,000 population)** | **9.Number of doctors (per 1,000 population)** | **10.Suicide rate (per 10,000 population)** |
| Miyama | -1.24 | 0.477153241 | 14.40346349 | 0.666056115 | 1.110093525 |
| Miyako | -1.36 | 0.993278789 | 8.222673787 | 0.330797221 | 5.670809508 |
| Ukiha | -1.04 | 0.19789774 | 14.06000355 | 0.426060714 | 1.065151784 |
| Munakata | -0.48 | 0.30792057 | 14.14095739 | 0.616611514 | 1.4387602 |
| Chikushino | -0.14 | 0.089210732 | 14.27927251 | 2.264016233 | 1.503081317 |
| Chikuzen | -0.49 | 0.381855417 | 15.85410254 | 0.430278357 | 1.985900109 |
| Chikugo | -0.27 | 0.098092137 | 10.06432238 | 0.852220847 | 1.420368078 |
| Chikujo | -1.13 | 0.321349891 | 3.444607669 | 0.050655995 | 1.013119903 |
| Nakama | -1.27 | 0.085405738 | 5.650837712 | 0.396550015 | 2.726281352 |
| Shime | -0.16 | 0.043718113 | 13.91569107 | 1.372271538 | 0.857669711 |
| Nogata | -0.88 | 0.197490143 | 17.6257128 | 0.875922847 | 1.251318353 |
| Onga | -0.75 | 0.196413796 | 18.02322526 | 1.839104619 | 2.10183385 |
| Umi | -0.39 | 0.075004894 | 16.18975904 | 0.349612737 | 1.613597246 |
| Kanda | -0.3 | 1.704845374 | 13.31337219 | 0.989146126 | 3.208041491 |
| Sue | -0.22 | 0.09179962 | 8.589884552 | 0.240516767 | 2.405167675 |
| Yukuhashi | -0.53 | 0.153833185 | 17.65643464 | 0.961829124 | 2.061062409 |
| Shingu | 0.05 | 0.06341915 | 3.721711988 | 0.270124257 | 0.600276127 |
| Sasaguri | -0.28 | 0.068117062 | 25.51184135 | 0.866439895 | 1.283614659 |
| Kotake | -1.8 | 0.429593839 | 10.26922009 | 0.277546489 | 0 |
| Ogori | -0.56 | 0.099542836 | 19.77911647 | 1.288487282 | 1.673360107 |
| Kawara | -1.66 | 0.281888736 | 1.797030171 | 0 | 0 |
| Tagawa | -1.12 | 0.156288379 | 45.71230683 | 2.597290161 | 1.29864508 |
| Soeda | -1.67 | 0.533544927 | 24.54123369 | 0.221092195 | 2.210921954 |
| Dazaifu | -0.43 | 0.049655494 | 9.885346756 | 0.363534676 | 1.677852349 |
| Mizumaki | -0.8 | 0.069884145 | 17.74785802 | 2.123983008 | 1.43998848 |
| Koge | -0.98 | 0.551777538 | 0 | 0 | 0 |
| Kasuya | 0.4 | 0.048747316 | 9.096147514 | 1.173032598 | 1.646361541 |
| Nakagawa | -0.25 | 0.15061931 | 4.190613027 | 0.0199553 | 1.59642401 |
| Itoda | -0.54 | 0.543853695 | 12.37964237 | 0.458505273 | 0 |
| Itoshima | -1.48 | 0.134435464 | 10.74054802 | 0.490884279 | 1.079945414 |
| Ashiya | -0.93 | 0.106238987 | 11.45096834 | 1.383338457 | 2.305564095 |
| Yanagawa | -1.07 | 0.147802501 | 16.41420063 | 0.750363457 | 1.563257203 |
| Buzen | -1.34 | 2.073341299 | 19.30973255 | 0.406910073 | 0 |
| Hisayama | -0.26 | 0.049197328 | 10.38511467 | 0.432713111 | 4.327131112 |
| Kurume | -0.49 | 0.083805112 | 23.9265305 | 3.819646542 | 1.653526642 |
| Kama | -1.52 | 0.791867356 | 24.68390805 | 0.833333333 | 0.574712644 |
| Yoshitomi | -0.95 | 0.0457193 | 10.30337722 | 0.429307384 | 0 |
| Keisen | -0.94 | 0.436667375 | 7.092751364 | 0.077942323 | 1.558846454 |
| Hirokawa | -0.71 | 0.20539677 | 24.9250181 | 1.396214707 | 1.551349674 |
| Koga | -0.34 | 0.07871757 | 25.17882206 | 2.147555676 | 0.845494361 |
| Miyawaka | -1.23 | 0.231539159 | 23.56218007 | 0.978620897 | 2.63474857 |
| Okagaki | -0.81 | 0.166882544 | 15.17363977 | 0.444416228 | 2.5395213 |
| Fukuchi | -1.37 | 0.302013621 | 24.83196415 | 0.466766243 | 3.267363704 |
| Fukutsu | -0.22 | 0.241783931 | 16.53471999 | 1.285384593 | 1.46066431 |
| Fukuoka | -0.15 | 0.037052641 | 14.54983692 | 2.103598563 | 1.855553749 |
| Iizuka | -0.78 | 0.017771707 | 22.97275641 | 3.725961538 | 1.923076923 |
| Toho | -2.29 | 0.324368213 | 0 | 0 | 5.327650506 |
| Onojo | 0.03 | 0.042030223 | 15.89346657 | 0.373732506 | 1.573610551 |
| Okawa | -0.43 | 0.130530778 | 9.860371383 | 0.215920541 | 2.159205412 |
| Oki | -1.36 | 1.295980738 | 41.8442675 | 2.176554876 | 1.451036584 |
| Omuta | -0.13 | 0.131685284 | 8.0020005 | 0.500125031 | 1.250312578 |
| Tachiarai | -1.08 | 0.123373959 | 20.24917303 | 3.246050638 | 2.164033759 |
| Oto | -1.5 | 0.524542362 | 3.651739381 | 0 | 0 |
| Kasuga | -0.15 | 0.174261448 | 9.86366499 | 1.703078847 | 1.774040466 |
| Kawasaki | -1.41 | 1.274771862 | 16.5471247 | 0.817918711 | 1.887504719 |
| Aka | -1.71 | 0.86047112 | 0 | 0 | 0 |
| Asakura | -1.06 | 0.154810945 | 16.66436859 | 1.300057124 | 1.181870112 |
| Kitakyushu | -0.75 | 0.608317034 | 21.14945863 | 2.348617022 | 1.980630945 |
| Yame | -1.01 | 0.260415582 | 23.7426011 | 1.570715254 | 1.653384478 |
| Kurate | -1.2 | 0.205113218 | 15.77935424 | 0.994497116 | 3.977988464 |

| Supplementary table 3: Raw data for FOHI | | | | | |
| --- | --- | --- | --- | --- | --- |
| **Municipality** | **11.Existence of livestock disease surveillance system (Yes=1; No=0)** | **12.Existence of Wild animal disease surveillance system (Yes=1; No=0)** | **13.Recycling rate (%)** | **14.One Health policy agreement in municipal council (Yes=1; No=0)** | **15.One Health declaration (Yes=1; No=0)** |
| Miyama | 1 | 1 | 39.6 | 1 | 1 |
| Miyako | 1 | 1 | 4.2 | 0 | 1 |
| Ukiha | 1 | 1 | 66.4 | 0 | 0 |
| Munakata | 1 | 1 | 23.8 | 1 | 0 |
| Chikushino | 1 | 1 | 22.3 | 0 | 0 |
| Chikuzen | 1 | 1 | 22.7 | 0 | 1 |
| Chikugo | 1 | 1 | 13.3 | 1 | 1 |
| Chikujo | 1 | 1 | 48.3 | 0 | 1 |
| Nakama | 1 | 1 | 18.9 | 0 | 1 |
| Shime | 1 | 1 | 63.5 | 0 | 0 |
| Nogata | 1 | 1 | 10.1 | 1 | 1 |
| Onga | 1 | 1 | 19 | 0 | 1 |
| Umi | 1 | 1 | 67.1 | 1 | 1 |
| Kanda | 1 | 1 | 69.6 | 0 | 1 |
| Sue | 1 | 1 | 65 | 0 | 0 |
| Yukuhashi | 1 | 1 | 12.9 | 0 | 1 |
| Shingu | 1 | 1 | 11.5 | 0 | 0 |
| Sasaguri | 1 | 1 | 65.4 | 0 | 0 |
| Kotake | 1 | 1 | 94 | 0 | 0 |
| Ogori | 1 | 1 | 26.8 | 0 | 1 |
| Kawara | 1 | 1 | 7.3 | 0 | 0 |
| Tagawa | 1 | 1 | 8.3 | 0 | 0 |
| Soeda | 1 | 1 | 6 | 0 | 1 |
| Dazaifu | 1 | 1 | 17.4 | 1 | 1 |
| Mizumaki | 1 | 1 | 17.7 | 0 | 1 |
| Koge | 1 | 1 | 11.5 | 0 | 1 |
| Kasuya | 1 | 1 | 62.6 | 0 | 0 |
| Nakagawa | 1 | 1 | 14.3 | 1 | 0 |
| Itoda | 1 | 1 | 13 | 0 | 0 |
| Itoshima | 1 | 1 | 23.3 | 0 | 0 |
| Ashiya | 1 | 1 | 19.5 | 0 | 0 |
| Yanagawa | 1 | 1 | 20.3 | 0 | 1 |
| Buzen | 1 | 1 | 9 | 0 | 1 |
| Hisayama | 1 | 1 | 1.1 | 0 | 0 |
| Kurume | 1 | 1 | 21.5 | 0 | 0 |
| Kama | 1 | 1 | 28.2 | 0 | 1 |
| Yoshitomi | 1 | 1 | 10.8 | 0 | 1 |
| Keisen | 1 | 1 | 18.1 | 0 | 0 |
| Hirokawa | 1 | 1 | 10.7 | 0 | 1 |
| Koga | 1 | 1 | 16.6 | 0 | 1 |
| Miyawaka | 1 | 1 | 94.1 | 1 | 1 |
| Okagaki | 1 | 1 | 19.4 | 1 | 1 |
| Fukuchi | 1 | 1 | 13.9 | 0 | 0 |
| Fukutsu | 1 | 1 | 26 | 0 | 0 |
| Fukuoka | 1 | 1 | 12.3 | 1 | 0 |
| Iizuka | 1 | 1 | 26.2 | 0 | 1 |
| Toho | 1 | 1 | 27.4 | 0 | 1 |
| Onojo | 1 | 1 | 20.4 | 0 | 1 |
| Okawa | 1 | 1 | 67 | 0 | 1 |
| Oki | 1 | 1 | 55.5 | 0 | 1 |
| Omuta | 1 | 1 | 25.8 | 0 | 0 |
| Tachiarai | 1 | 1 | 22.2 | 0 | 1 |
| Oto | 1 | 1 | 3.2 | 0 | 0 |
| Kasuga | 1 | 1 | 15.1 | 0 | 0 |
| Kawasaki | 1 | 1 | 4.3 | 0 | 0 |
| Aka | 1 | 1 | 7.3 | 0 | 0 |
| Asakura | 1 | 1 | 18.8 | 1 | 1 |
| Kitakyushu | 1 | 1 | 24.2 | 1 | 1 |
| Yame | 1 | 1 | 13.7 | 0 | 1 |
| Kurate | 1 | 1 | 93.9 | 0 | 0 |

| Supplementary table 3: Raw data for FOHI | | | | | |
| --- | --- | --- | --- | --- | --- |
| **Municipality** | **16.Number of registered agricultural, forestry, fishery products (per 10,000 population)** | **17.Number of Registered One Health declaration businesses implementing One Health Practice (per 10,000 population)** | **18.One Health website presence scale (3=dedicated website; 2=dedicated webpage; 1=section on government website; 0=no web presence)** | **19.Number of One Health promotion and/or education facilities** | **20.Influenza vaccination rate for age 65+ (%)** |
| Miyama | 346.3491799 | 29.97252519 | 3 | 2 | 63.65990505 |
| Miyako | 0.472567459 | 5.198242049 | 0 | 0 | 60.86503387 |
| Ukiha | 310.6692704 | 2.485354163 | 0 | 0 | 56.55778643 |
| Munakata | 0.822148686 | 0.616611514 | 0 | 0 | 60.02047083 |
| Chikushino | 5.73049752 | 1.033368405 | 0 | 0 | 57.37556888 |
| Chikuzen | 1.654916758 | 1.654916758 | 0 | 0 | 65.92398427 |
| Chikugo | 0.608729176 | 12.7833127 | 0 | 0 | 58.9298785 |
| Chikujo | 0 | 1.013119903 | 0 | 0 | 61.70698311 |
| Nakama | 0.495687519 | 1.734906315 | 1 | 0 | 60.88262175 |
| Shime | 0 | 0.643252284 | 0 | 0 | 56.96418931 |
| Nogata | 1.608837883 | 3.038916001 | 2 | 1 | 59.35275081 |
| Onga | 2.627292313 | 1.050916925 | 0 | 0 | 43.79650721 |
| Umi | 0 | 0 | 0 | 0 | 53.91166012 |
| Kanda | 17.10955462 | 2.138694327 | 0 | 0 | 58.16944828 |
| Sue | 0 | 1.030786146 | 0 | 0 | 57.8892372 |
| Yukuhashi | 69.3891011 | 2.473274891 | 1 | 0 | 61.23128879 |
| Shingu | 0 | 0.300138064 | 0 | 0 | 58.83266066 |
| Sasaguri | 0 | 1.283614659 | 0 | 0 | 58.04412334 |
| Kotake | 0 | 1.387732445 | 0 | 0 | 65.82781457 |
| Ogori | 26.94109772 | 1.673360107 | 0 | 0 | 62.66237636 |
| Kawara | 0 | 1.891610707 | 0 | 0 | 63.95056077 |
| Tagawa | 6.276784555 | 3.463053547 | 0 | 0 | 62.74833386 |
| Soeda | 4.421843909 | 3.316382932 | 0 | 1 | 67.21146593 |
| Dazaifu | 0.139821029 | 1.398210291 | 1 | 0 | 60.23144929 |
| Mizumaki | 0 | 0.71999424 | 0 | 0 | 53.70490387 |
| Koge | 0 | 6.298022421 | 0 | 0 | 65.16105146 |
| Kasuya | 1.852156734 | 0.411590385 | 0 | 0 | 57.23601419 |
| Nakagawa | 0 | 1.197318008 | 0 | 0 | 55.39033457 |
| Itoda | 1.146263182 | 0 | 0 | 0 | 45.2181987 |
| Itoshima | 41.13610258 | 0.589061135 | 0 | 0 | 55.19568587 |
| Ashiya | 0.768521365 | 3.842606824 | 0 | 0 | 59.88870856 |
| Yanagawa | 122.2467133 | 5.158748769 | 0 | 0 | 65.03593145 |
| Buzen | 34.40239707 | 4.439018977 | 1 | 0 | 61.91638017 |
| Hisayama | 0 | 0 | 0 | 0 | 51.27795527 |
| Kurume | 22.52103286 | 2.612572094 | 0 | 0 | 58.67508261 |
| Kama | 0 | 1.436781609 | 1 | 0 | 60.52850028 |
| Yoshitomi | 0 | 4.293073841 | 0 | 0 | 61.39933365 |
| Keisen | 0 | 1.558846454 | 0 | 0 | 64.08768536 |
| Hirokawa | 0 | 1.034233116 | 0 | 0 | 61.15499386 |
| Koga | 0 | 0.676395488 | 2 | 0 | 56.62050534 |
| Miyawaka | 0 | 6.398675098 | 1 | 0 | 61.93426441 |
| Okagaki | 0.317440163 | 1.26976065 | 0 | 0 | 61.10953328 |
| Fukuchi | 0 | 2.800597461 | 0 | 0 | 62.78801843 |
| Fukutsu | 0 | 0.730332155 | 0 | 0 | 60.96826989 |
| Fukuoka | 9.169368697 | 2.06597737 | 0 | 0 | 58.12739853 |
| Iizuka | 13.78205128 | 3.525641026 | 1 | 0 | 60.83434489 |
| Toho | 5.327650506 | 10.65530101 | 0 | 0 | 61.74496644 |
| Onojo | 0 | 0.983506594 | 2 | 0 | 58.76929863 |
| Okawa | 285.0151144 | 4.318410825 | 0 | 0 | 55.1316761 |
| Oki | 0 | 1.723105944 | 0 | 1 | 59.69014979 |
| Omuta | 5.001250313 | 0.625156289 | 0 | 0 | 62.64764876 |
| Tachiarai | 0.92744304 | 5.564658237 | 0 | 0 | 47.27334408 |
| Oto | 0 | 1.921968095 | 0 | 0 | 57.72020725 |
| Kasuga | 0 | 0.620914163 | 0 | 0 | 56.92052213 |
| Kawasaki | 0.62916824 | 1.258336479 | 0 | 0 | 65.04226753 |
| Aka | 3.342245989 | 0 | 0 | 0 | 64.00329489 |
| Asakura | 152.0672878 | 3.939567042 | 1 | 0 | 64.7897402 |
| Kitakyushu | 3.008827337 | 1.158073831 | 1 | 0 | 60.01115142 |
| Yame | 560.0013227 | 3.472107404 | 2 | 0 | 61.8645979 |
| Kurate | 0 | 3.977988464 | 0 | 0 | 61.32375063 |

| Supplementary table 3: Raw data for FOHI | | | | | |
| --- | --- | --- | --- | --- | --- |
| **Municipality** | **21.Influenza Weekly Average Number of Cases per Sentinel Surveillance Site** | **22.COVID-19 Weekly Average Number of Cases per Sentinel Surveillance Site** | **23.Infectious Gastroenteritis Weekly Average Number of Cases per Sentinel Surveillance Site** | **24.Tuberculosis incidence (per 100,000 population)** | **25.Food waste per capita (kg)** |
| Miyama | 9.349038462 | 5.656730769 | 6.548461538 | 8.976222858 | 22.45 |
| Miyako | 8.233269231 | 4.630192308 | 3.411538462 | 11.94460782 | 1.81 |
| Ukiha | 7.963846154 | 6.961923077 | 3.888461538 | 6.446457112 | 411.27 |
| Munakata | 11.59769231 | 6.445384615 | 2.223076923 | 7.104198413 | 43.61 |
| Chikushino | 14.29980769 | 7.106923077 | 7.492307692 | 6.800784126 | 42.98 |
| Chikuzen | 7.963846154 | 6.961923077 | 3.888461538 | 6.446457112 | 60.41 |
| Chikugo | 9.349038462 | 5.656730769 | 6.548461538 | 8.976222858 | 84.54 |
| Chikujo | 8.233269231 | 4.630192308 | 3.411538462 | 11.94460782 | 2.15 |
| Nakama | 11.59769231 | 6.445384615 | 2.223076923 | 7.104198413 | 16.48 |
| Shime | 10.8525 | 4.860769231 | 5.294230769 | 6.145116179 | 16.58 |
| Nogata | 8.140576923 | 5.402307692 | 3.121153846 | 4.537885096 | 17.61 |
| Onga | 11.59769231 | 6.445384615 | 2.223076923 | 7.104198413 | 12.40 |
| Umi | 10.8525 | 4.860769231 | 5.294230769 | 6.145116179 | 5.43 |
| Kanda | 8.233269231 | 4.630192308 | 3.411538462 | 11.94460782 | 8.98 |
| Sue | 10.8525 | 4.860769231 | 5.294230769 | 6.145116179 | 26.49 |
| Yukuhashi | 8.233269231 | 4.630192308 | 3.411538462 | 11.94460782 | 8.29 |
| Shingu | 10.8525 | 4.860769231 | 5.294230769 | 6.145116179 | 161.83 |
| Sasaguri | 10.8525 | 4.860769231 | 5.294230769 | 6.145116179 | 13.70 |
| Kotake | 8.140576923 | 5.402307692 | 3.121153846 | 4.537885096 | 1.24 |
| Ogori | 7.963846154 | 6.961923077 | 3.888461538 | 6.446457112 | 6.55 |
| Kawara | 3.326923077 | 3.500192308 | 2.471153846 | 14.0303671 | 6.00 |
| Tagawa | 3.326923077 | 3.500192308 | 2.471153846 | 14.0303671 | 349.21 |
| Soeda | 3.326923077 | 3.500192308 | 2.471153846 | 14.0303671 | 2.82 |
| Dazaifu | 14.29980769 | 7.106923077 | 7.492307692 | 6.800784126 | 22.96 |
| Mizumaki | 11.59769231 | 6.445384615 | 2.223076923 | 7.104198413 | 9.22 |
| Koge | 8.233269231 | 4.630192308 | 3.411538462 | 11.94460782 | 0.68 |
| Kasuya | 10.8525 | 4.860769231 | 5.294230769 | 6.145116179 | 77.69 |
| Nakagawa | 14.29980769 | 7.106923077 | 7.492307692 | 6.800784126 | 11.06 |
| Itoda | 3.326923077 | 3.500192308 | 2.471153846 | 14.0303671 | 3.06 |
| Itoshima | 12.42134615 | 4.269615385 | 5.913461538 | 2.945305674 | 102.59 |
| Ashiya | 11.59769231 | 6.445384615 | 2.223076923 | 7.104198413 | 2.07 |
| Yanagawa | 9.349038462 | 5.656730769 | 6.548461538 | 8.976222858 | 7.12 |
| Buzen | 8.233269231 | 4.630192308 | 3.411538462 | 11.94460782 | 10.78 |
| Hisayama | 10.8525 | 4.860769231 | 5.294230769 | 6.145116179 | 195.73 |
| Kurume | 4.406923077 | 2.825192308 | 3.455192308 | 7.275517223 | 192.70 |
| Kama | 8.140576923 | 5.402307692 | 3.121153846 | 4.537885096 | 4.75 |
| Yoshitomi | 8.233269231 | 4.630192308 | 3.411538462 | 11.94460782 | 7.73 |
| Keisen | 8.140576923 | 5.402307692 | 3.121153846 | 4.537885096 | 112.78 |
| Hirokawa | 9.349038462 | 5.656730769 | 6.548461538 | 8.976222858 | 87.30 |
| Koga | 10.8525 | 4.860769231 | 5.294230769 | 6.145116179 | 136.72 |
| Miyawaka | 8.140576923 | 5.402307692 | 3.121153846 | 4.537885096 | 222.68 |
| Okagaki | 11.59769231 | 6.445384615 | 2.223076923 | 7.104198413 | 6.67 |
| Fukuchi | 3.326923077 | 3.500192308 | 2.471153846 | 14.0303671 | 17.36 |
| Fukutsu | 11.59769231 | 6.445384615 | 2.223076923 | 7.104198413 | 45.66 |
| Fukuoka | 60.24826923 | 24.34711538 | 14.02230769 | 9.11835691 | 196.19 |
| Iizuka | 8.140576923 | 5.402307692 | 3.121153846 | 4.537885096 | 27.33 |
| Toho | 7.963846154 | 6.961923077 | 3.888461538 | 6.446457112 | 0.00 |
| Onojo | 14.29980769 | 7.106923077 | 7.492307692 | 6.800784126 | 19.01 |
| Okawa | 9.349038462 | 5.656730769 | 6.548461538 | 8.976222858 | 7.31 |
| Oki | 9.349038462 | 5.656730769 | 6.548461538 | 8.976222858 | 13.22 |
| Omuta | 7.963846154 | 6.961923077 | 3.888461538 | 6.446457112 | 12.72 |
| Tachiarai | 9.349038462 | 5.656730769 | 6.548461538 | 8.976222858 | 4.89 |
| Oto | 3.326923077 | 3.500192308 | 2.471153846 | 14.0303671 | 69.31 |
| Kasuga | 14.29980769 | 7.106923077 | 7.492307692 | 6.800784126 | 7.69 |
| Kawasaki | 3.326923077 | 3.500192308 | 2.471153846 | 14.0303671 | 81.77 |
| Aka | 3.326923077 | 3.500192308 | 2.471153846 | 14.0303671 | 0.00 |
| Asakura | 7.963846154 | 6.961923077 | 3.888461538 | 6.446457112 | 747.43 |
| Kitakyushu | 11.97480769 | 5.130961538 | 5.134230769 | 11.14781351 | 37.69 |
| Yame | 9.349038462 | 5.656730769 | 6.548461538 | 8.976222858 | 53.30 |
| Kurate | 8.140576923 | 5.402307692 | 3.121153846 | 4.537885096 | 6.27 |

| Supplementary table 3: Raw data for FOHI | | | | | |
| --- | --- | --- | --- | --- | --- |
| **Municipality** | **26.Good Agricultural Practices (GAP) certified agricultural producers (per 10,000 population)** | **27.Food chain inspection implementation rate (%) *7** | **28.Arable land per capita (ha)** | **29.Local food promotion restaurants (per 10,000 population)** | **30.AMR surveillance system (Yes=1; No=0)** |
| Miyama | 0 | 41.0723492 | 0.112396969 | 4.440374102 | 1 |
| Miyako | 0 | 41.0723492 | 0.109163083 | 2.835404754 | 1 |
| Ukiha | 0.355050595 | 35.7845 | 0.091603053 | 0.355050595 | 1 |
| Munakata | 0 | 57.4590649 | 0.020553717 | 6.268883728 | 1 |
| Chikushino | 0 | 71.44416151 | 0.008210582 | 1.503081317 | 1 |
| Chikuzen | 0.330983352 | 71.44416151 | 0.077781088 | 5.957700328 | 1 |
| Chikugo | 0 | 71.44416151 | 0.039364487 | 2.029097255 | 1 |
| Chikujo | 0 | 71.44416151 | 0.095233271 | 6.585279368 | 1 |
| Nakama | 0 | 71.44416151 | 0.006766135 | 0.991375037 | 1 |
| Shime | 0 | 71.44416151 | 0.001265063 | 0.643252284 | 1 |
| Nogata | 0 | 71.44416151 | 0.011619385 | 2.681396471 | 1 |
| Onga | 1.576375388 | 40.28352038 | 0.0354159 | 10.50916925 | 1 |
| Umi | 0 | 40.28352038 | 0.002474182 | 0.537865749 | 1 |
| Kanda | 0 | 40.28352038 | 0.014115383 | 0.802010373 | 1 |
| Sue | 0 | 40.28352038 | 0.004707257 | 0.687190764 | 1 |
| Yukuhashi | 0.412212482 | 29.66543456 | 0.02706862 | 1.786254088 | 1 |
| Shingu | 0 | 40.28352038 | 0.004952278 | 3.001380635 | 1 |
| Sasaguri | 0 | 43.6462585 | 0.006706887 | 0.320903665 | 1 |
| Kotake | 0 | 43.6462585 | 0.02095476 | 1.387732445 | 1 |
| Ogori | 0 | 43.6462585 | 0.030789826 | 2.510040161 | 1 |
| Kawara | 0 | 43.6462585 | 0.044736593 | 0.945805353 | 1 |
| Tagawa | 0.64932254 | 43.6462585 | 0.01430674 | 2.813731007 | 1 |
| Soeda | 0 | 43.6462585 | 0.057262879 | 3.316382932 | 1 |
| Dazaifu | 0.279642058 | 43.6462585 | 0.00185962 | 1.398210291 | 1 |
| Mizumaki | 0 | 43.6462585 | 0.003815969 | 1.07999136 | 1 |
| Koge | 0 | 38.81909548 | 0.125582567 | 5.038417937 | 1 |
| Kasuya | 0 | 38.81909548 | 0.005083141 | 0.617385578 | 1 |
| Nakagawa | 0 | 38.81909548 | 0.007483238 | 1.795977011 | 1 |
| Itoda | 0 | 38.81909548 | 0.041136103 | 1.146263182 | 1 |
| Itoshima | 0.294530567 | 38.81909548 | 0.017996332 | 7.952325319 | 1 |
| Ashiya | 0 | 38.81909548 | 0.006378727 | 5.379649554 | 1 |
| Yanagawa | 0 | 38.81909548 | 0.060810705 | 3.439165846 | 1 |
| Buzen | 0.369918248 | 38.81909548 | 0.062516184 | 5.548773721 | 1 |
| Hisayama | 0 | 29.66543456 | 0.023691043 | 0 | 1 |
| Kurume | 0.330705328 | 29.66543456 | 0.027680036 | 4.299169268 | 1 |
| Kama | 0 | 29.66543456 | 0.05316092 | 2.586206897 | 1 |
| Yoshitomi | 0 | 29.66543456 | 0.027189468 | 2.862049227 | 1 |
| Keisen | 1.558846454 | 29.66543456 | 0.033749026 | 3.897116134 | 1 |
| Hirokawa | 0 | 29.66543456 | 0.035887889 | 2.58558279 | 1 |
| Koga | 0 | 29.66543456 | 0.00754181 | 2.198285337 | 1 |
| Miyawaka | 0 | 45.68490635 | 0.064363144 | 1.129177958 | 1 |
| Okagaki | 0 | 71.44416151 | 0.017617929 | 3.80928195 | 1 |
| Fukuchi | 0.466766243 | 69.48912015 | 0.037528006 | 0.933532487 | 1 |
| Fukutsu | 0.146066431 | 69.48912015 | 0.016505507 | 4.966258654 | 1 |
| Fukuoka | 0.108400047 | 69.48912015 | 0.001122259 | 3.366777936 | 1 |
| Iizuka | 0.320512821 | 69.48912015 | 0.018910256 | 2.96474359 | 1 |
| Toho | 0 | 69.48912015 | 0.140649973 | 10.65530101 | 1 |
| Onojo | 0 | 69.48912015 | 0.000501588 | 0.491753297 | 1 |
| Okawa | 1.439470275 | 65.7083533 | 0.070318123 | 3.598675687 | 1 |
| Oki | 0 | 65.7083533 | 0.009613117 | 1.723105944 | 1 |
| Omuta | 0 | 65.7083533 | 0.075643911 | 1.250312578 | 1 |
| Tachiarai | 0 | 65.7083533 | 0.040189198 | 3.400624478 | 1 |
| Oto | 1.921968095 | 65.7083533 | 0.056890256 | 0 | 1 |
| Kasuga | 0 | 65.7083533 | 0.000204015 | 0.53221214 | 1 |
| Kawasaki | 0 | 65.7083533 | 0.02535548 | 2.516672958 | 1 |
| Aka | 0 | 43.6462585 | 0.127005348 | 16.71122995 | 1 |
| Asakura | 0.787913408 | 41.0723492 | 0.096125436 | 2.757696929 | 1 |
| Kitakyushu | 0.01082312 | 41.0723492 | 0.002316148 | 2.218739583 | 1 |
| Yame | 0.330676896 | 41.0723492 | 0.105651268 | 3.802784299 | 1 |
| Kurate | 0.662998077 | 41.0723492 | 0.054962541 | 1.325996155 | 1 |

| Supplementary table 3: Raw data for FOHI | | | | |
| --- | --- | --- | --- | --- |
| **Municipality** | **31.Japan Nosocomial Infections Surveillance (JANIS) hospitals (per 10,000 population)** | **32.CO2 emissions (tonnes/capita)** | **33.CO2 emissions (kg/GDP)** | **34.Days with WBGT index above 25 (days/year)** |
| Miyama | 0.277523381 | 2.232826231 | 5.560160543 | Missing |
| Miyako | 0 | 3.325131343 | 9.45134918 | Missing |
| Ukiha | 0 | 2.577802265 | 7.749149098 | Missing |
| Munakata | 0.616611514 | 1.719568586 | 3.775130404 | 111 |
| Chikushino | 0.469712911 | 1.371924376 | 3.457662365 | Missing |
| Chikuzen | 0 | 2.35842641 | 5.543117789 | Missing |
| Chikugo | 0 | 2.789939797 | 10.34524599 | Missing |
| Chikujo | 0 | 1.698982362 | 4.913631528 | Missing |
| Nakama | 0.495687519 | 2.34746934 | 5.303856449 | Missing |
| Shime | 0.428834856 | 1.521863938 | 4.267928045 | Missing |
| Nogata | 0.35751953 | 2.444707209 | 8.759228473 | Missing |
| Onga | 0.525458463 | 2.051153997 | 6.042772319 | Missing |
| Umi | 0 | 1.983591903 | 5.681541943 | Missing |
| Kanda | 0.802010373 | 8.63693035 | 58.70715928 | Missing |
| Sue | 0.687190764 | 2.160657926 | 6.562671798 | Missing |
| Yukuhashi | 0.274808321 | 2.1692607 | 6.128225563 | 110 |
| Shingu | 0 | 2.149202544 | 8.437029436 | Missing |
| Sasaguri | 0 | 1.644624447 | 4.627241288 | Missing |
| Kotake | 0 | 3.067274009 | 10.62521424 | Missing |
| Ogori | 0.669344043 | 1.551227843 | 4.215025576 | Missing |
| Kawara | 0 | 2.219777376 | 5.323644653 | Missing |
| Tagawa | 0.432881693 | 2.155396825 | 6.722914181 | Missing |
| Soeda | 0 | 2.268233105 | 4.311297811 | 112 |
| Dazaifu | 0.279642058 | 1.475873075 | 3.439597315 | 122 |
| Mizumaki | 0.35999712 | 2.020803308 | 4.643962848 | Missing |
| Koge | 0 | 3.410459926 | 10.77523193 | Missing |
| Kasuya | 0.411590385 | 1.546106677 | 5.399187025 | Missing |
| Nakagawa | 0.598659004 | 1.925016912 | 3.742665884 | Missing |
| Itoda | 1.146263182 | 2.975904771 | 3.553415864 | Missing |
| Itoshima | 0.196353712 | 0.072924442 | 0.166900655 | 122 |
| Ashiya | 0.768521365 | 1.263699653 | 4.226867507 | Missing |
| Yanagawa | 0.625302881 | 1.845624688 | 5.110703228 | Missing |
| Buzen | 0 | 3.260852924 | 10.8930316 | Missing |
| Hisayama | 1.081782778 | 2.180358215 | 15.84109023 | Missing |
| Kurume | 0.429916927 | 1.736198334 | 5.94277475 | 125 |
| Kama | 0.862068966 | 2.592220689 | 6.256116063 | Missing |
| Yoshitomi | 0 | 3.339037522 | 11.44819691 | Missing |
| Keisen | 0 | 2.513217726 | 5.455034079 | Missing |
| Hirokawa | 0 | 2.570922997 | 10.7020752 | Missing |
| Koga | 0.507296616 | 2.429443194 | 9.85769674 | Missing |
| Miyawaka | 0.376392653 | 10.0503112 | 76.31018812 | Missing |
| Okagaki | 0.634880325 | 1.837652874 | 3.682305885 | Missing |
| Fukuchi | 0 | 2.866122314 | 5.291704076 | Missing |
| Fukutsu | 0.292132862 | 1.650856431 | 3.442414964 | Missing |
| Fukuoka | 0.68228265 | 0.942114468 | 4.532307908 | 116 |
| Iizuka | 0.400641026 | 1.998331195 | 6.677452512 | 114 |
| Toho | 0 | 1.545595054 | 6.925945658 | Missing |
| Onojo | 0.393402638 | 1.67350887 | 4.311914265 | Missing |
| Okawa | 0 | 2.566452369 | 6.395442663 | Missing |
| Oki | 0.634828506 | 2.081388498 | 8.243990502 | 122 |
| Omuta | 0 | 2.521461953 | 6.297822543 | Missing |
| Tachiarai | 0.61829536 | 2.004937831 | 6.860002608 | Missing |
| Oto | 0 | 2.51934497 | 5.381510667 | Missing |
| Kasuga | 0.354808093 | 1.25923112 | 2.698784824 | Missing |
| Kawasaki | 0.62916824 | 2.840589504 | 4.755583623 | Missing |
| Aka | 0 | 2.933698416 | 5.013368984 | Missing |
| Asakura | 0.196978352 | 2.259230976 | 15.51680972 | 121 |
| Kitakyushu | 0.487040396 | 2.083216956 | 8.681519662 | 110 |
| Yame | 0.661353791 | 2.116614457 | 7.365455754 | 113 |
| Kurate | 0.662998077 | 3.207205625 | 15.58885227 | Missing |
