# Supplementary material for: Adaptation of global One Health evaluation framework to municipal levels in Fukuoka, Japan
Source: Infect Dis Poverty. 2025 Nov 13;14:116. doi: 10.1186/s40249-025-01380-y (PMC12613462; doi:10.1186/s40249-025-01380-y)
Supplement: Supplementary file 2 — Supplementary Material 2. Supplemental material Fuzzy Analytic Hierarchy Process (FAHP). [file 40249_2025_1380_MOESM2_ESM.docx]

**The Weighting Methodology Using Fuzzy Analytic Hierarchy Process (FAHP)**

***Fuzzy Analytic Hierarchy Process (FAHP) Implementation***

Based on the binary judgments from each pairwise question, we constructed a fuzzy judgment matrix $\tilde{A}$ to represent the collective expert opinions. The matrix was constructed as follows:

The fuzzy judgment matrix $\tilde{A}$ was constructed as:

$$\tilde{A}=\left[ \begin{matrix} 1 & n_{12} & n_{13} & \cdots& n_{1m} \\ \frac{1}{n_{12}} & 1 & n_{23} & \cdots& n_{2m} \\ \frac{1}{n_{13}} & \frac{1}{n_{23}} & 1 & \cdots& n_{3m} \\ \vdots& \vdots& \vdots& \ddots& \vdots\\ \frac{1}{n_{1m}} & \frac{1}{n_{2m}} & \frac{1}{n_{3m}} & \cdots& 1 \end{matrix} \right]$$

where:

- $n_{ij}$ represents the number of times indicator $i$ was preferred over indicator $j$ in the binary pairwise comparisons across all 23 experts.
- The value $n_{ij}$ can range from 0 to 23, indicating how many experts chose indicator $i$ over indicator $j$.
- The reciprocal relationship between matrix elements is maintained as $a_{ji}=\frac{1}{a_{ij}}$, ensuring mathematical consistency in the preference structure.

To transform the binary expert judgments into quantitative values suitable for mathematical analysis, we employed a systematic approach:

For each pairwise comparison, we tallied the total number of experts (out of 23) who preferred indicator $i$ over indicator $j$. These frequency counts directly formed the elements $n_{ij}$ of the fuzzy judgment matrix. When all 23 experts agreed on a preference, this represented the strongest possible consensus; when opinions were divided (e.g., 12 vs 11), this represented maximum uncertainty. This approach effectively converts simple binary judgments into a rich quantitative framework that captures both the direction and strength of collective expert preferences while preserving the fuzzy nature of the aggregated judgments.

The weight vector $W$ was calculated using the geometric mean method, which is particularly suitable for fuzzy hierarchical analyses due to its ability to minimize the influence of extreme values and maintain the ratio scale properties. The formula used was:

$$w_{i}=\frac{(\prod_{j=1}^{n} a_{ij})^{1/n}}{\sum_{k=1}^{n} (\prod_{j=1}^{n} a_{kj})^{1/n}}$$

where:

- $w_{i}$ represents the normalized weight of indicator $i$
- $a_{ij}$ represents the fuzzy preference value of indicator i over indicator $j$
- $n$ is the total number of indicators being compared

This approach ensures that:

1. The weights are normalized (sum to 1).
2. The relative importance between any two indicators is preserved.
3. The influence of inconsistencies in judgments is minimized.

The weight calculation was performed at multiple hierarchical levels:

First, weights were derived for the *EDI IDI* and *CDI*. Then, weights were calculated for the 13 key indicators within their respective categories

***Consistency Verification***

To ensure the reliability of the derived weights, we calculated the Consistency Ratio (CR) for each expert's judgment matrix using the formula:

$$CR=\frac{CI}{RI}$$

where:

- $CI$ (Consistency Index) = $\frac{\lambda_{max}-n}{n-1}$
- $\lambda_{max}$ is the maximum eigenvalue of the judgment matrix
- $RI$ is the Random Index (a predefined value based on matrix size)

For matrices with $CR$ ≤ 0.1, the judgments were considered consistent. For any expert judgments exceeding this threshold, we conducted follow-up clarifications to resolve inconsistencies.
