# Supplementary material for: Adaptation of global One Health evaluation framework to municipal levels in Fukuoka, Japan
Source: Infect Dis Poverty. 2025 Nov 13;14:116. doi: 10.1186/s40249-025-01380-y (PMC12613462; doi:10.1186/s40249-025-01380-y)
Supplement: Supplementary file 3 — Supplementary Material 3.Supplemental material Latent Class Analysis (LCA). [file 40249_2025_1380_MOESM3_ESM.docx]

**Latent Class Analysis Methodology**

***Latent Class Analysis***

To scientifically classify municipalities in Fukuoka, we employed Latent Class Analysis (LCA) as the primary analytical method. LCA is a statistical technique based on finite mixture models that identifies latent class structures according to patterns of observed indicators.

***Data Transformation***

We discretized the three key continuous index variables (EDI, IDI, and CDI) according to the following boundary values to facilitate the latent class analysis:

$$EDI_{cat}=\left\{ \begin{aligned} &\text{Below 40.001}, &&\text{if }EDI<40.01 \\ &\text{40.001-50.000}, &&\text{if }40.01\leq EDI<50 \\ &\text{50.001-60.000}, &&\text{if }50.01\leq EDI<60 \\ &\text{Above 60}, &&\text{if }EDI\geq60 \end{aligned} \right.$$

$IDI_{cat}$ and $CDI_{cat}$ were categorized using the same classification boundaries.

***Model Construction***

For the three categorical variables ($EDI_{cat}$, $IDI_{cat}$, $CDI_{cat}$) across Fukuoka's 60 municipalities, we established the following latent class model:

$$P\left( Y=y \right)=\sum_{c=1}^{C} \gamma_{c}\prod_{j=1}^{3} \prod_{r_{j}=1}^{4} \rho_{j,r_{j}|c}^{I\left( y_{j}=r_{j} \right)}$$

where:

- $Y=(Y_{1},Y_{2},Y_{3})$ represents the three observed variables ($EDI_{cat}$, $IDI_{cat}$, $CDI_{cat}$)
- $C$is the number of latent classes (we tested $C=2,3,4,5,6$)
- $\gamma_{c}=P(X=c)$ is the prior probability of latent class $c$
- $\rho_{j,r_{j}|c}=P(Y_{j}=r_{j}|X=c)$ is the conditional item response probability, representing the probability that variable $j$ takes the value $r_{j}$ conditional on class $c$
- $I(y_{j}=r_{j})$ is the indicator function

***Likelihood Function***

For the observational data from 60 municipalities, the log-likelihood function is represented as:

$$\ln L=\sum_{i=1}^{60} \ln\left[ \sum_{c=1}^{C} \gamma_{c}\prod_{j=1}^{3} \prod_{r_{j}=1}^{4} \rho_{j,r_{j}|c}^{I\left( y_{ij}=r_{j} \right)} \right]$$

We utilized the Expectation-Maximization (EM) algorithm for estimation, setting the maximum number of iterations to 3000, convergence tolerance to 0.00001, and employing 10 different random starting values to avoid local maxima.

***Model Selection***

To determine the optimal number of classes, we calculated the Bayesian Information Criterion (BIC) for each model:

$$BIC\left( C \right)=-2\ln L+\mathrm{pln} \left( 60 \right)$$

where $p$ is the number of free parameters in the model:

$$p=\left( C-1 \right)+C\sum_{j=1}^{3} \left( 4-1 \right)=\left( C-1 \right)+9C=10C-1$$

We selected the model with the minimum BIC value as the optimal number of classes:

$$C^{*}=\arg\min_{C} BIC\left( C \right)$$

Results indicated that the $C^{*}=2$ class model had the minimum BIC value (423.09), therefore we selected the two-class model as optimal.

| **Supplementary Table D1.** Fitness indicators of different latent classes | | | |
| --- | --- | --- | --- |
| Models | BIC | AIC | LogLikelihood |
| 2-class | 418.527 | 378.735 | -170.367 |
| 3-class | 454.280 | 393.544 | -167.772 |
| 4-class | 488.707 | 407.028 | -164.514 |
| 5-class | 526.677 | 424.054 | -163.027 |
| 6-class | 566.732 | 443.166 | -162.583 |

***Conditional Probability Estimation***

The conditional probability matrices for the 2-class model are shown as follows:

For $EDI_{cat}(j=1)$:

$$\hat{\rho}_{1,r|c}=\left( \begin{matrix} 0.1923 & 0.6538 & 0.0000 & 0.1538 \\ 0.0000 & 0.0000 & 1.0000 & 0.0000 \end{matrix} \right)$$

For $IDI_{cat}(j=2)$:

$$\hat{\rho}_{2,r|c}=\left( \begin{matrix} 0.0000 & 0.0769 & 0.5000 & 0.4231 \\ 0.0882 & 0.0000 & 0.3529 & 0.5588 \end{matrix} \right)$$

For $CDI_{cat}(j=3)$:

$$\hat{\rho}_{3,r|c}=\left( \begin{matrix} 0.1538 & 0.6538 & 0.1154 & 0.0769 \\ 0.0588 & 0.7059 & 0.2353 & 0.0000 \end{matrix} \right)$$

where rows represent classes (c=1,2) and columns represent the four possible values of the categorical variables.

***Class Size***

The estimated class prior probabilities were:

$$\hat{\gamma}_{1}=0.4333,\hat{\gamma}_{2}=0.5667$$

***Posterior Class Assignment***

For each municipality $i$, we calculated its posterior probability of belonging to each class:

$$P\left( X=c | Y=y_{i} \right)=\frac{\gamma_{c}\prod_{j=1}^{3} \rho_{j,y_{ij}|c}}{\sum_{c^{'}=1}^{2} \gamma_{c^{'}}\prod_{j=1}^{3} \rho_{j,y_{ij}|c^{'}}}$$

Then we assigned each municipality to the class with the highest posterior probability:

$$\hat{c}_{i}=\arg\max_{c} P\left( X=c | Y=y_{i} \right)$$

The final results classified 26 municipalities into Class 1 and 34 municipalities into Class 2, with the classification proportions consistent with the estimated class prior probabilities.

Through this mathematical model and computational process, we successfully categorized Fukuoka's 60 municipalities into two classes: one primarily characterized by lower environmental development levels but higher institutional development levels, and the other characterized by high levels in both environmental and institutional development.
