# Supplementary material for: Adaptation of global One Health evaluation framework to municipal levels in Fukuoka, Japan
Source: Infect Dis Poverty. 2025 Nov 13;14:116. doi: 10.1186/s40249-025-01380-y (PMC12613462; doi:10.1186/s40249-025-01380-y)
Supplement: Supplementary file 4 — Supplementary Material 4. Technical File for Fukuoka One Health Indicators Detailed Information. [file 40249_2025_1380_MOESM4_ESM.docx]

| 1 | |
| --- | --- |
| Indicator Title | Forest area (% of total land area) |
| Indicator Code | A1.1 |
| Raw Data Title | Forest Area_Current Forest Area |
| Data Unit | % |
| Value Range | (0,100) |
| Years Available | 2020 |
| Municipalities Available | 60 |
| Data base | 2020 Census of Agriculture and Forestry Confirmed Report Vol. 1 Prefectural Statistical Book (Fukuoka Prefecture) |
| Download Link | https://www.e-stat.go.jp/dbview?sid=0002072478 |
| Published by | Ministry of Agriculture, Forestry and Fisheries |
| Note | Forest ratio = Forest area/Total land area; Total land area can also be obtained from the same Database and Download Link. |

| 2 | |
| --- | --- |
| Indicator Title | Biochemical Oxygen Demand level (mg/L) |
| Indicator Code | A1.2 |
| Raw Data Title | BOD_75% value |
| Data Unit | / |
| Value Range | (0,99999) |
| Years Available | 2022 |
| Municipalities Available | 17 |
| Data base | Hydrological and Water Quality Database |
| Download Link | http://www1.river.go.jp/ |
| Published by | Ministry of Land, Infrastructure, Transport and Tourism |
| Note | / |

| 3 | |
| --- | --- |
| Indicator Title | Government revenue (% of GDP) |
| Indicator Code | A2.1 |
| Raw Data Title | Total Revenue |
| Data Unit | % |
| Value Range | (0,100) |
| Years Available | 2022 |
| Municipalities Available | 60 |
| Data base | Summary of Fukuoka Prefecture Municipal Ordinary Accounts Settlement and Financial Soundness Indicators for Fiscal Year 2022 (Page 11) |
| Download Link | https://www.pref.fukuoka.lg.jp/uploaded/attachment/204008.pdf |
| Published by | Fukuoka Prefectural Government |
| Note | Government revenue (% of GDP) = Total Revenue/Total Production; Total Production is based on the Fukuoka Prefecture Statistical Yearbook (Fiscal Year 2022) published by Fukuoka Prefectural Government: https://ckan.open-governmentdata.org/dataset/401000_tokeinenkan_r04/resource/6461aed2-b7b6-415e-a07b-9807fbc50cfa |

| 4 | |
| --- | --- |
| Indicator Title | Municipal regulations for pet evacuation during disasters (Yes=1; No=0) |
| Indicator Code | A2.2 |
| Raw Data Title | Municipalities with regulations for pet evacuation during disasters (Yes/No) |
| Data Unit | Yes/No |
| Value Range | (0-1) |
| Years Available | 2022 |
| Municipalities Available | 60 |
| Data base | / |
| Download Link | / |
| Published by | / |
| Note | Fukuoka Prefecture Government Internal Data |

| 5 | |
| --- | --- |
| Indicator Title | Unemployment (% of total labor force) |
| Indicator Code | A3.1 |
| Raw Data Title | Unemployment Rate |
| Data Unit | People |
| Value Range | (0,999999) |
| Years Available | 2020 |
| Municipalities Available | 60 |
| Data base | "Population Census, Population Estimates" |
| Download Link | https://www.e-stat.go.jp/regional-statistics/ssdsview/municipality |
| Published by | Statistics Bureau of Japan, Census Statistics Division |
| Note | / |

| 6 | |
| --- | --- |
| Indicator Title | Natural population growth rate (%) |
| Indicator Code | A4.1 |
| Raw Data Title | Natural Population Change Rate |
| Data Unit | % |
| Value Range | (-100,100) |
| Years Available | 2022 |
| Municipalities Available | 60 |
| Data base | [Total] Basic Resident Register Population and Number of Households in 2023, Vital Statistics in 2022 (by Municipality) |
| Download Link | https://www.soumu.go.jp/menu_news/s-news/01gyosei02_02000289.html |
| Published by | Ministry of Internal Affairs and Communications |
| Note | / |

| 7 | | |
| --- | --- | --- |
| Indicator Title | Renewable electricity generation (% of total electricity demand) |  |
| Indicator Code | A5.1 |  |
| Raw Data Title | Reverse Power Flow from Renewable Energy Generation |  |
| Data Unit | 1,000kWh |  |
| Value Range | (0,9999999) |  |
| Years Available | 2022 |  |
| Municipalities Available | 60 |  |
| Data base | Electric Power Survey Statistics |  |
| Download Link | https://www.enecho.meti.go.jp/statistics/electric_power/ep002/results.html |  |
| Published by | Agency for Natural Resources and Energy, Ministry of Economy, Trade and Industry |  |
| Note | Renewable electricity generation (% of total electricity demand) = Reverse Power Flow from Renewable Energy Generation/Electricity Demand; Electricity Demand can also be obtained from the same Database and Download Link. |  |

| 8 | |
| --- | --- |
| Indicator Title | Number of hospital and clinic beds (per 1,000 population) |
| Indicator Code | B1.1 |
| Raw Data Title | Number of Hospital Beds |
| Data Unit | Beds |
| Value Range | (0,9999999) |
| Years Available | 2022 |
| Municipalities Available | 60 |
| Data base | Fukuoka Prefecture Medical Statistics for 2021 |
| Download Link | https://www.pref.fukuoka.lg.jp/uploaded/attachment/209820.pdf |
| Published by | Fukuoka Prefecture Health, Medical and Long-term Care General Affairs Division |
| Note | Number of hospital and clinic beds (per 1,000 population)= Number of Hospital Beds/Population; Population refers to Fukuoka Prefecture Basic Resident Register Annual Report (FY2021) https://www.pref.fukuoka.lg.jp/contents/juki-jukinenpou.html |

| 9 | |
| --- | --- |
| Indicator Title | Number of doctors (per 1,000 population) |
| Indicator Code | B1.2 |
| Raw Data Title | Number of Full-time Doctors |
| Data Unit | People |
| Value Range | (0,9999999) |
| Years Available | 2022 |
| Municipalities Available | 60 |
| Data base | Fukuoka Prefecture Medical Statistics 2021 |
| Download Link | https://www.pref.fukuoka.lg.jp/uploaded/attachment/209820.pdf |
| Published by | Fukuoka Prefecture Health, Medical and Long-term Care General Affairs Division |
| Note | Number of doctors (per 1,000 population) = Number of Full-time Doctors/Population; Population refers to Fukuoka Prefecture Basic Resident Register Annual Report (FY2021) https://www.pref.fukuoka.lg.jp/contents/juki-jukinenpou.html |

| 10 | |
| --- | --- |
| Indicator Title | Suicide rate (per 10,000 population) |
| Indicator Code | B1.3 |
| Raw Data Title | Number of Suicides |
| Data Unit | People |
| Value Range | (0,9999999) |
| Years Available | 2022 |
| Municipalities Available | 60 |
| Data base | Suicide Statistics: Basic Information on Suicide in Regions (2022) |
| Download Link | https://www.mhlw.go.jp/stf/seisakunitsuite/bunya/0000197204_00008.html |
| Published by | Ministry of Health, Labour and Welfare |
| Note | Suicide rate (per 10,000 population) = Number of Suicides/Total Population; Total population data can be obtained from "Fukuoka Prefecture Population 2022" at: https://ckan.open-governmentdata.org/dataset/401000_jinkouidouchousa-jinkousetai/resource/b92206f1-1845-4dad-964c-69df74068119 |

| 11 | |
| --- | --- |
| Indicator Title | Existence of livestock disease surveillance system (Yes=1; No=0) |
| Indicator Code | B2.1 |
| Raw Data Title | Existence of livestock disease surveillance system |
| Data Unit | Yes/No |
| Value Range | (0-1) |
| Years Available | 2024 |
| Municipalities Available | 60 |
| Data base | / |
| Download Link | / |
| Published by | / |
| Note | Fukuoka Prefecture Government Internal Data |

| 12 | |
| --- | --- |
| Indicator Title | Existence of Wild animal disease surveillance system (Yes=1; No=0) |
| Indicator Code | B2.2 |
| Raw Data Title | Existence of Wild animal disease surveillance system |
| Data Unit | Yes/No |
| Value Range | (0-1) |
| Years Available | 2024 |
| Municipalities Available | 60 |
| Data base | / |
| Download Link | / |
| Published by | / |
| Note | Fukuoka Prefecture Government Internal Data |

| 13 | |
| --- | --- |
| Indicator Title | Recycling rate (%) |
| Indicator Code | B3.1 |
| Raw Data Title | Waste Recycling Rate |
| Data Unit | % |
| Value Range | (0,100) |
| Years Available | 2022 |
| Municipalities Available | 60 |
| Data base | Current Status of Municipal Waste Management in Fukuoka Prefecture, Fiscal Year 2022 Edition (Page 96) |
| Download Link | https://www.pref.fukuoka.lg.jp/uploaded/life/721150_62076338_misc.pdf |
| Published by | Fukuoka Prefecture Environment Department |
| Note | Recycling rate = (Total amount of resources recycled + Amount collected by group collection)/(Total amount of waste processed + Amount collected by group collection) × 100 |

| 14 | |
| --- | --- |
| Indicator Title | One Health policy agreement in municipal council (Yes=1; No=0) |
| Indicator Code | C1.1 |
| Raw Data Title | Municipalities that have passed a resolution in the municipal assembly regarding One Health promotion |
| Data Unit | Yes/No |
| Value Range | (0-1) |
| Years Available | 2024 |
| Municipalities Available | 60 |
| Data base | Fukuoka Prefecture One Health Promotion Portal Site: Municipal Initiatives |
| Download Link | https://onehealth.pref.fukuoka.lg.jp/about-one-health/municipality/ |
| Published by | Fukuoka Prefecture |
| Note | / |

| 15 | |
| --- | --- |
| Indicator Title | One Health declaration (Yes=1; No=0) |
| Indicator Code | C1.2 |
| Raw Data Title | Municipalities that have made a declaration regarding One Health promotion |
| Data Unit | Yes/No |
| Value Range | (0-1) |
| Years Available | 2022 |
| Municipalities Available | 60 |
| Data base | Fukuoka Prefecture One Health Promotion Portal Site: Municipal Initiatives |
| Download Link | https://onehealth.pref.fukuoka.lg.jp/about-one-health/municipality/ |
| Published by | Fukuoka Prefecture |
| Note | / |

| 16 | |
| --- | --- |
| Indicator Title | Number of registered agricultural, forestry, fishery products (per 10,000 population) |
| Indicator Code | C1.3 |
| Raw Data Title | Number of entities with One Health certification |
| Data Unit | Number |
| Value Range | (0,9999999) |
| Years Available | 2024 |
| Municipalities Available | 60 |
| Data base | Fukuoka One Health Certification: List of Certified Agricultural, Forestry and Fishery Products, Producers and Organizations |
| Download Link | https://onehealth-certify.pref.fukuoka.lg.jp/certification/search/ |
| Published by | Fukuoka Prefecture |
| Note | / |

| 17 | |
| --- | --- |
| Indicator Title | Number of Registered One Health declaration businesses implementing One Health Practice (per 10,000 population) |
| Indicator Code | C1.4 |
| Raw Data Title | Number of Registered One Health Declaration Businesses |
| Data Unit | Number |
| Value Range | (0,9999999) |
| Years Available | 2024 |
| Municipalities Available | 60 |
| Data base | Fukuoka One Health: One Health Declaration Business Search |
| Download Link | https://onehealth.pref.fukuoka.lg.jp/registrations/search/ |
| Published by | Fukuoka Prefecture |
| Note | / |

| 18 | |
| --- | --- |
| Indicator Title | One Health website presence scale (3=dedicated website; 2=dedicated webpage; 1=section on government website; 0=no web presence) |
| Indicator Code | C1.5 |
| Raw Data Title | Municipalities with One Health special site |
| Data Unit | Number (0-3) |
| Value Range | (0/1/2/3) |
| Years Available | 2024 |
| Municipalities Available | 60 |
| Data base | / |
| Download Link | https://www.city.miyama.lg.jp/onehealth/index.html |
| Published by | / |
| Note | - |

| 19 | |
| --- | --- |
| Indicator Title | Number of One Health promotion and/or education facilities |
| Indicator Code | C1.6 |
| Raw Data Title | Municipalities with One Health awareness certified facilities |
| Data Unit | Yes/No |
| Value Range | (0-1) |
| Years Available | 2024 |
| Municipalities Available | 60 |
| Data base | Fukuoka Prefecture One Health Awareness: Certified Facilities (6 facilities) |
| Download Link | https://www.pref.fukuoka.lg.jp/contents/onehealth-place.html#2itiran |
| Published by | Fukuoka Prefecture |
| Note | - |

| 20 | |
| --- | --- |
| Indicator Title | Influenza vaccination rate for age 65+ (%) |
| Indicator Code | C2.1 |
| Raw Data Title | Influenza vaccination numbers for age 65+ |
| Data Unit | number |
| Value Range | (0,9999999) |
| Years Available | 2022 |
| Municipalities Available | 60 |
| Data base | e-Stat Government Statistics |
| Download Link | https://www.e-stat.go.jp/stat-search/files?page=1&query=%E6%8E%A5%E7%A8%AE&layout=dataset&kikan=00450 |
| Published by | Ministry of Health, Labour and Welfare |
| Note | Vaccination rate equals vaccination numbers divided by population aged 65 and over; population data for those aged 65 and over can be found at the same link. |

| 21 | |
| --- | --- |
| Indicator Title | Influenza Weekly Average Number of Cases per Sentinel Surveillance Site |
| Indicator Code | C2.2 |
| Raw Data Title | Influenza Weekly Average Number of Cases per Sentinel Surveillance Site |
| Data Unit | Number |
| Value Range | (0,9999999) |
| Years Available | 2022 |
| Municipalities Available | 12 |
| Data base | Fukuoka Prefecture Infectious Disease Surveillance Weekly Report |
| Download Link | https://ckan.open-governmentdata.org/dataset/401002_kansenshoushuuhou |
| Published by | Fukuoka Prefecture |
| Note | Influenza, COVID-19, and infectious gastroenteritis are the top 3 annual infections |

| 22 | |
| --- | --- |
| Indicator Title | COVID-19 Weekly Average Number of Cases per Sentinel Surveillance Site |
| Indicator Code | C2.3 |
| Raw Data Title | COVID-19 Weekly Average Number of Cases per Sentinel Surveillance Site |
| Data Unit | Number |
| Value Range | (0,9999999) |
| Years Available | 2022 |
| Municipalities Available | 12 |
| Data base | Fukuoka Prefecture Infectious Disease Surveillance Weekly Report |
| Download Link | https://ckan.open-governmentdata.org/dataset/401002_kansenshoushuuhou |
| Published by | Fukuoka Prefecture |
| Note | / |

| 23 | |
| --- | --- |
| Indicator Title | Infectious Gastroenteritis Weekly Average Number of Cases per Sentinel Surveillance Site |
| Indicator Code | C2.4 |
| Raw Data Title | Infectious Gastroenteritis Weekly Average Number of Cases per Sentinel Surveillance Site |
| Data Unit | Number |
| Value Range | (0,9999999) |
| Years Available | 2022 |
| Municipalities Available | 12 |
| Data base | Fukuoka Prefecture Infectious Disease Surveillance Weekly Report |
| Download Link | https://ckan.open-governmentdata.org/dataset/401002_kansenshoushuuhou |
| Published by | Fukuoka Prefecture |
| Note | / |

| 24 | |
| --- | --- |
| Indicator Title | Tuberculosis incidence (per 100,000 population) |
| Indicator Code | C2.5 |
| Raw Data Title | Tuberculosis incidence |
| Data Unit | Number |
| Value Range | (0,9999999) |
| Years Available | 2022 |
| Municipalities Available | 12 |
| Data base | Tuberculosis in Fukuoka Prefecture 2023 |
| Download Link | https://www.pref.fukuoka.lg.jp/contents/fukuoka-tb2023.html |
| Published by | Fukuoka Prefecture Health, Medical and Long-term Care Department Cancer Infectious Disease Control Division |
| Note | / |

| 25 | |  |
| --- | --- | --- |
| Indicator Title | Food waste (kg/per capita) | |
| Indicator Code | C3.1 | |
| Raw Data Title | Food Loss | |
| Data Unit | kg | |
| Value Range | (0,9999999) | |
| Years Available | 2022 | |
| Municipalities Available | 60 | |
| Data base | Results of Regular Reporting Based on the Food Recycling Law | |
| Download Link | https://www.maff.go.jp/j/shokusan/recycle/syokuhin/s_houkoku/kekka/gaiyou.html | |
| Published by | Ministry of Agriculture, Forestry and Fisheries | |
| Note | Food waste per capita = Amount of food waste divided by municipal population | |

| 26 | |
| --- | --- |
| Indicator Title | Good Agricultural Practices (GAP) certified agricultural producers (per 10,000 population) |
| Indicator Code | C3.2 |
| Raw Data Title | Good Agricultural Practices (GAP) certified agricultural producers |
| Data Unit | number |
| Value Range | (0,9999999) |
| Years Available | 2022 |
| Municipalities Available | 60 |
| Data base | List of Fukuoka GAP Certified Producers (PDF) |
| Download Link | https://www.pref.fukuoka.lg.jp/uploaded/attachment/220760.pdf |
| Published by | Fukuoka Prefecture |
| Note |  |

| 27 | |
| --- | --- |
| Indicator Title | Food chain inspection implementation rate (%) |
| Indicator Code | C3.3 |
| Raw Data Title | Food chain inspection implementation rate |
| Data Unit | % |
| Value Range | (0,9999999) |
| Years Available | 2022 |
| Municipalities Available | 60 |
| Data base | Monitoring and Guidance Implementation Results FY 2021-2024 |
| Download Link | / |
| Published by | Fukuoka Prefecture Health and Medical Care Department, Environmental Health Division |
| Note | Fukuoka Prefecture Government Internal Data |

| 28 | |
| --- | --- |
| Indicator Title | Arable land per capita (ha) |
| Indicator Code | C3.4 |
| Raw Data Title | Arable Land Area (Cultivated Land) |
| Data Unit | Number |
| Value Range | (0,9999999) |
| Years Available | 2022 |
| Municipalities Available | 60 |
| Data base | Area Survey 2022 |
| Download Link | https://www.machimura.maff.go.jp/machi/map/40/index.html |
| Published by | Ministry of Agriculture, Forestry and Fisheries |
| Note | Includes temporarily cultivated land (counted once for double cropping), temporary meadows for mowing or pasture, land for market or kitchen gardens, and temporarily fallow land |

| 29 | |
| --- | --- |
| Indicator Title | Local food promotion restaurants (per 10,000 population) |
| Indicator Code | C3.5 |
| Raw Data Title | Grain Import Dependence |
| Data Unit | Number |
| Value Range | (0,9999999) |
| Years Available | 2024 |
| Municipalities Available | 60 |
| Data base | Fukuoka Agriculture, Forestry and Fisheries Support Group: List of Supporting Restaurants |
| Download Link | https://f-ouen.com/ouen/shop/shop-list/ |
| Published by | Fukuoka Agriculture, Forestry and Fisheries Support Group |
| Note | List of local food promotion restaurants (searchable by municipality) |

| 30 | |
| --- | --- |
| Indicator Title | AMR surveillance system (Yes=1; No=0) |
| Indicator Code | C4.1 |
| Raw Data Title | AMR in humans’ surveillance system |
| Data Unit | Number |
| Value Range | (0-1) |
| Years Available | 2024 |
| Municipalities Available | 60 |
| Data base | / |
| Download Link | / |
| Published by | / |
| Note | Fukuoka Prefecture Government Internal Data |

| 31 | |
| --- | --- |
| Indicator Title | Japan Nosocomial Infections Surveillance (JANIS) hospitals (per 10,000 population) |
| Indicator Code | C4.2 |
| Raw Data Title | Japan Nosocomial Infections Surveillance (JANIS) hospitals |
| Data Unit | Number |
| Value Range | (0,9999999) |
| Years Available | 2022 |
| Municipalities Available | 60 |
| Data base | JANIS - Japan Nosocomial Infections Surveillance |
| Download Link | https://janis.mhlw.go.jp/hospitallist/index.html |
| Published by | Ministry of Health, Labour and Welfare |
| Note |  |

| 32 | |
| --- | --- |
| Indicator Title | CO2 emissions (tonnes/capita) |
| Indicator Code | C5.1 |
| Raw Data Title | CO2 Emissions |
| Data Unit | kg |
| Value Range | (0,9999999) |
| Years Available | 2021 |
| Municipalities Available | 60 |
| Data base | Regional Decarbonization Local Government Action Plan |
| Download Link | https://www.env.go.jp/policy/local_keikaku/tools/suikei.html |
| Published by | Ministry of the Environment |
| Note | CO2 emissions (tonnes/capita)= CO2 emissions /Total Population |

| 33 | |
| --- | --- |
| Indicator Title | CO2 emissions (kg/GDP) |
| Indicator Code | C5.2 |
| Raw Data Title | CO2 Emissions |
| Data Unit | kg |
| Value Range | (0,9999999) |
| Years Available | 2021 |
| Municipalities Available | 60 |
| Data base | Regional Decarbonization Local Government Action Plan |
| Download Link | https://www.env.go.jp/policy/local_keikaku/tools/suikei.html |
| Published by | Ministry of the Environment |
| Note | CO2 emissions (kg/GDP)= CO2 emissions /Total Production; Total Production is based on the Fukuoka Prefecture Statistical Yearbook (Fiscal Year 2021) published by Fukuoka Prefectural Government: https://ckan.open-governmentdata.org/dataset/401000_tokeinenkan_r03 |

| 34 | |
| --- | --- |
| Indicator Title | Days with WBGT index above 25 (days/year) |
| Indicator Code | C5.3 |
| Raw Data Title | WBGT |
| Data Unit | Number |
| Value Range | (0,9999999) |
| Years Available | 2022 |
| Municipalities Available | 12 |
| Data base | Heat Stroke Prevention Measures Site |
| Download Link | https://www.wbgt.env.go.jp/wbgt.php |
| Published by | Ministry of the Environment |
| Note | WBGT above 25 (warning level) |
