# Supplementary material for: Adaptation of global One Health evaluation framework to municipal levels in Fukuoka, Japan
Source: Infect Dis Poverty. 2025 Nov 13;14:116. doi: 10.1186/s40249-025-01380-y (PMC12613462; doi:10.1186/s40249-025-01380-y)
Supplement: Supplementary file 5 — Supplementary Material 5. Questionnaire for weighting. [file 40249_2025_1380_MOESM5_ESM.docx]

**Survey on Priority Assessment for the Development of Fukuoka Prefecture's One Health Indicator Evaluation System**

In developing the One Health Indicator Evaluation System for Fukuoka Prefecture, we are requesting cooperation from experts familiar with various fields to participate in a survey regarding the weighting of evaluation items. In this survey, you will be asked to compare two evaluation items and select the one you consider more important. The survey is expected to take approximately 5-10 minutes to complete. We sincerely apologize for imposing on your busy schedule and would greatly appreciate your cooperation.

**1. Regarding the evaluation scores for *External Drivers Index* in each municipality of Fukuoka Prefecture, which do you consider more important in each of the following pairs? Please enter "1" below the option you consider more important.**

| Earth Systems | Institutional Systems |
| --- | --- |
|  |  |
| Earth Systems | Economic Systems |
|  |  |
| Earth Systems | Social Systems |
|  |  |
| Earth Systems | Technological Systems |
|  |  |
| Institutional Systems | Economic Systems |
|  |  |
| Institutional Systems | Social Systems |
|  |  |
| Institutional Systems | Technological Systems |
|  |  |
| Economic Systems | Social Systems |
|  |  |
| Economic Systems | Technological Systems |
|  |  |
| Social Systems | Technological Systems |
|  |  |

**2. Regarding the evaluation scores for *Internal Drivers Index* in each municipality of Fukuoka Prefecture, which do you consider more important in each of the following pairs? Please enter "1" below the option you consider more important.**

| Human Health | Animal Health and Ecosystem Diversity |
| --- | --- |
|  |  |
| Human Health | Environmental Resources |
|  |  |
| Animal Health and Ecosystem Diversity | Environmental Resources |
|  |  |

**3. Regarding the evaluation scores for *Core Driver Index* in each municipality of Fukuoka Prefecture, which do you consider more important in each of the following pairs? Please enter "1" below the option you consider more important.**

| One Health Governance | Zoonotic Diseases |
| --- | --- |
|  |  |
| One Health Governance | Food Security |
|  |  |
| One Health Governance | ANTIMICROBIAL RESISTANCE (AMR) |
|  |  |
| One Health Governance | Climate Change |
|  |  |
| Zoonotic Diseases | Food Security |
|  |  |
| Zoonotic Diseases | ANTIMICROBIAL RESISTANCE (AMR) |
|  |  |
| Zoonotic Diseases | Climate Change |
|  |  |
| Food Security | ANTIMICROBIAL RESISTANCE (AMR) |
|  |  |
| Food Security | Climate Change |
|  |  |
| ANTIMICROBIAL RESISTANCE (AMR) | Climate Change |
|  |  |

**4. Regarding the *One Health evaluation* scores for each municipality in Fukuoka Prefecture, which do you consider more important in each of the following pairs? Please enter "1" below the option you consider more important.**

| External Drivers Index | Internal Drivers Index |
| --- | --- |
|  |  |
| External Drivers Index | Core Driver Index |
|  |  |
| Internal Drivers Index | Core Driver Index |
|  |  |

Finally, we would like to ask about your profile as an expert.

5. Please indicate your age: (　)

a.21-30 b.31-40 c.41-50 d.51-60 　　　　d. Over 60

6. Please indicate your biological sex: (　)

a. Male b. Female c. Prefer not to answer

7. Please indicate your field of specialization: (　)

a. Human Health b. Veterinary Medicine c. Environmental Science d. Sociology e. Political Science f. Legal Studies g. Economics/Business Administration

8. Please indicate your years of professional experience: (　)

a. Less than 10 years b. 10-20 years c. More than 20 years
