# Supplementary material for: Adaptation of global One Health evaluation framework to municipal levels in Fukuoka, Japan
Source: Infect Dis Poverty. 2025 Nov 13;14:116. doi: 10.1186/s40249-025-01380-y (PMC12613462; doi:10.1186/s40249-025-01380-y)
Supplement: Supplementary file 6 — Supplementary Material 6. List of GOHI Sub-Indicators. [file 40249_2025_1380_MOESM6_ESM.docx]

| Original GOHI sub-indicators | Status | Rationale |
| --- | --- | --- |
| A1.1.1 Country area | Deleted | Not applicable at municipal level; designed for national-level assessment with no meaningful variation within a prefecture |
| A1.1.2 Cultivated area | Deleted | Replaced by more specific and normalized agricultural indicators (arable land per capita) that better reflect local food security capacity |
| A1.1.3 Arable land area | Retained | Retained as FOHI C3.4 "Arable land per capita (ha)" to measure local food production capacity with direct relevance to food security |
| A1.1.4 Terrain Ruggedness Index | Deleted | Limited relevance to One Health priorities in Fukuoka's mostly developed urban/rural context; minimal impact on health outcomes at municipal level |
| A1.2.1 Forest area | Retained | Retained as FOHI A1.1 "Forest area (% of total land area)" as key environmental indicator with direct implications for biodiversity and ecosystem health |
| A1.2.2 Forest Transition Phase | Deleted | Data unavailable at municipal level; concept more applicable to large-scale national forest management rather than municipal conservation efforts |
| A1.2.3 Trees per capita | Deleted | Data unavailable at municipal level; urban greenspace better captured through forest area percentage for Fukuoka's context |
| A1.2.4 Permanent deforestation | Deleted | Not a significant ongoing concern for Fukuoka municipalities; stable forest cover with minimal deforestation activity in recent decades |
| A1.3.1 Renewable water resources | Deleted | While important, centralized water management in Japan makes municipal-level assessment impractical; not a differentiating factor among municipalities |
| A1.3.2 Water dependency ratio | Deleted | Shared water resources across municipalities in Fukuoka make this metric non-discriminatory for municipal comparisons |
| A1.3.3 Water Stress | Retained | Retained as FOHI A1.2 "Biochemical Oxygen Demand level (mg/L)" to measure water quality with direct implications for environmental and human health |
| A1.4.1 CO2 emissions | Retained | Retained as FOHI C5.1 "CO2 emissions (tons/capita)" and C5.2 "CO2 emissions (kg/GDP)" as critical climate change indicators with municipal variation |
| A1.4.2 Air pollution index | Deleted | Limited monitoring stations at municipal level; regional air quality patterns make this less valuable for municipal-level differentiation |
| A1.5.1 Disasters Death Rate | Deleted | Extremely low rates across most municipalities with minimal meaningful variation; more relevant at regional or national levels |
| A1.5.2 Disaster Economic Loss | Deleted | Inconsistent disaster occurrence makes historical data poor predictor of future resilience; better captured through preparedness measures |
| A1.5.3 Disasters Affected Population | Deleted | Highly variable and event-dependent; not a stable indicator for ongoing One Health capacity assessment |
| A2.1.1 Unsentenced detainees | Deleted | Legal system indicators have minimal direct relevance to One Health implementation at municipal level in Fukuoka context |
| A2.1.2 Property Rights | Deleted | Uniform property rights system across Japan makes this non-discriminatory for municipal comparisons |
| A2.1.3 Corruption Perception Index | Deleted | National-level metric with no municipal variation; minimal direct relevance to One Health implementation |
| A2.1.4 Press Freedom Index | Deleted | National-level metric with no municipal variation; minimal direct relevance to One Health implementation |
| A2.1.5 Affordability of justice | Deleted | Standardized legal system across Japan makes this non-discriminatory for municipal comparisons |
| A2.2.1 Voice and Accountability | Deleted | National governance metric with limited municipal variation; not directly linked to operational One Health outcomes |
| A2.2.2 Government Spending | Retained | Retained as FOHI A2.1 "Government revenue (% of GDP)" as indicator of municipal fiscal capacity to support One Health initiatives |
| A2.2.3 Public Social Expenditure | Deleted | Overlaps with other more specific indicators; municipal budget allocations not consistently categorized across municipalities |
| A2.2.4 Public Education Expenditure | Deleted | While education is important, other indicators more directly measure One Health implementation capacity at municipal level |
| A2.2.5 Public Health Expenditure | Deleted | Better captured through more specific health infrastructure and service indicators in FOHI |
| A2.2.6 Political Stability | Deleted | Minimal variation across Japanese municipalities; not a differentiating factor for One Health implementation |
| A2.2.7 Government Effectiveness | Deleted | Too abstract for municipal assessment; better captured through specific One Health policy and implementation indicators |
| A2.2.8 Regulatory Quality | Deleted | National-level regulatory framework with limited municipal variation in Japan's centralized system |
| A2.2.9 Rule of Law | Deleted | Uniform application across Japan makes this non-discriminatory for municipal comparisons |
| A2.2.10 Control of Corruption | Deleted | Limited variation across Japanese municipalities; not a significant barrier to One Health implementation |
| A3.1.1 Gross Domestic Product | Deleted | Used as denominator for other metrics rather than standalone indicator; absolute GDP less relevant than per capita measures |
| A3.1.2 GDP Deflator | Deleted | Macroeconomic indicator more relevant at national level; minimal municipal variation with limited One Health implications |
| A3.1.3 Revenue excluding grants | Deleted | Municipal finance structures vary significantly, making cross-municipality comparisons difficult and less meaningful |
| A3.1.4 Grants and other revenue | Deleted | Complex funding mechanisms across municipalities with limited standardization for comparative assessment |
| A3.1.5 Adjusted GDP growth | Deleted | Short-term economic fluctuations less relevant than structural economic indicators for One Health capacity |
| A3.2.1 Labor force participation | Deleted | Demographic indicator with limited direct connection to One Health implementation capacity |
| A3.2.2 Unemployment | Retained | Retained as FOHI A3.1 "Unemployment (% of total labor force)" as indicator of socioeconomic stability affecting health outcomes |
| A3.2.3 Annual Working Hours | Deleted | Work-life culture fairly uniform across Japanese municipalities; limited One Health implementation relevance |
| A3.2.4 Youth condition | Deleted | Demographic indicator with limited direct connection to One Health implementation capacity |
| A3.3.1 Own outright | Deleted | Housing ownership patterns not directly relevant to One Health implementation in Japanese municipal context |
| A3.3.2 Rent at reduced/subsidised price | Deleted | Housing subsidy programs typically administered at national level with limited municipal variation |
| A4.1.1 Natural Population Growth | Retained | Retained as FOHI A4.1 "Natural population growth rate (%)" as key demographic indicator affecting health system planning |
| A4.1.2 Life Expectancy | Deleted | Limited variation across Fukuoka municipalities; national health insurance creates uniformity in outcomes |
| A4.1.3 Child and Infant Mortality | Deleted | Extremely low rates across Japan with minimal municipal variation; not a differentiating factor |
| A4.1.4 Total Fertility Rate | Deleted | Demographic indicator with limited direct connection to One Health implementation capacity |
| A4.1.5 Urbanization | Deleted | Static classification rather than dynamic indicator; better captured through population density and distribution |
| A4.2.1 Education enrollment | Deleted | Compulsory education system in Japan creates high uniformity; limited relevance to One Health implementation |
| A4.2.2 Literacy | Deleted | Near universal literacy in Japan with negligible municipal variation; not a differentiating factor |
| A4.2.3 PISA score | Deleted | Educational assessments conducted at national level without municipal breakdown; not applicable for local comparison |
| A4.2.4 Science performance | Deleted | Educational assessments conducted at national level without municipal breakdown; not applicable for local comparison |
| A4.2.5 Higher Education | Deleted | Educational attainment less directly relevant to municipal One Health implementation than specific health indicators |
| A4.2.6 Expenditure on research | Deleted | Research funding often distributed through universities and national programs, not consistently tracked at municipal level |
| A4.2.7 Female graduates | Deleted | Gender education parity, while important, has limited direct relevance to One Health implementation in this context |
| A4.2.8 Researchers population | Deleted | Research capacity typically concentrated in specific municipalities with universities; skewed distribution limits usefulness |
| A4.3.1 Gini coefficient | Deleted | Income inequality measures not calculated at municipal level in Japan; limited relevance to operational One Health capacity |
| A4.3.2 Palma ratio | Deleted | Income distribution metrics not calculated at municipal level in Japan; limited direct One Health implementation relevance |
| A4.3.3 Human Development Index | Deleted | Composite national-level metric not calculated for municipalities; components better assessed individually |
| A4.3.4 Poverty rate | Deleted | Different measurement methodologies across municipalities limit comparability; national welfare programs reduce variation |
| A4.3.5 Gender Inequality Index | Deleted | Composite national-level metric not calculated for municipalities; limited direct One Health implementation relevance |
| A5.1.1 Railway Travel | Deleted | Transportation infrastructure, while important for access, has limited direct relevance to One Health implementation |
| A5.1.2 Air Travel | Deleted | Only few municipalities in Fukuoka have airports; not a meaningful comparative metric for most municipalities |
| A5.2.1 Internet Population | Deleted | High internet penetration across Japan with minimal municipal variation; not a differentiating factor |
| A5.2.2 Motor Vehicle Ownership | Deleted | Transportation patterns vary by urban/rural status but have limited direct One Health implementation relevance |
| A5.2.3 Mobile Cellular Subscriptions | Deleted | Near universal mobile access in Japan with minimal municipal variation; not a differentiating factor |
| A5.2.4 Logistics Performance Index | Deleted | National-level logistics assessment without municipal breakdown; not applicable for local comparison |
| A5.2.5 Access to electricity | Deleted | Universal electricity access in Japan with no municipal variation; not a differentiating factor |
| A5.2.6 Share of renewable energy | Retained | Retained as FOHI A5.1 "Renewable electricity generation (% of total electricity demand)" as key sustainability indicator |
| A5.3.1 Energy Consumption | Deleted | Better reflected through emissions indicators; absolute consumption less relevant than efficiency metrics |
| A5.3.2 Electricity Consumption | Deleted | Better reflected through emissions indicators; absolute consumption less relevant than clean energy transition |
| A5.3.3 Solid waste | Deleted | Better captured through more specific waste recycling indicator in FOHI that reflects circular economy efforts |
| A5.3.4 Electronic waste | Deleted | E-waste management primarily administered through prefectural programs with limited municipal-level data |
| A5.3.5 SO₂ emissions | Deleted | Industrial emissions monitored at limited sites; not comprehensive enough for meaningful municipal comparison |
| A5.3.6 Nitrogen emissions | Deleted | Agricultural emissions difficult to attribute precisely to municipalities; monitoring systems insufficient |
| A5.3.7 Non-recycled waste | Retained | Retained as FOHI B3.1 "Waste disposal recycling rate (%)" as indicator of environmental sustainability and circular economy |
| B1.1.1 Life expectancy | Deleted | Minimal variation across municipalities due to universal healthcare system; not a differentiating factor |
| B1.1.2 Health service coverage | Adapted | Adapted as FOHI B1.1 "Number of hospital and clinic beds (per 1,000 population)" to assess concrete healthcare infrastructure |
| B1.1.3 Domestic health expenditure | Deleted | National health insurance standardizes much of healthcare financing; municipal variations less meaningful |
| B1.1.4 Health risks | Deleted | Too broad and composite for specific municipal assessment; better captured through specific risk indicators |
| B1.2.1 Infectious diseases | Deleted | Better captured through specific zoonotic disease surveillance indicators in FOHI with greater relevance |
| B1.2.2 Non-communicable diseases | Deleted | NCD prevalence data not consistently collected across municipalities; major NCDs better tracked nationally |
| B1.2.3 Mental health | Retained | Retained as FOHI B1.3 "Suicide rate (per 10,000 population)" as key mental health indicator with municipal variation |
| B1.3.1 Road traffic | Deleted | Traffic safety, while important, has limited direct relevance to core One Health implementation |
| B1.3.2 Homicide | Deleted | Extremely low rates across Japan with minimal municipal variation; not a significant public health concern |
| B2.1.1 Diseases of domestic animal | Adapted | Adapted as FOHI B2.1 "Existence of livestock disease surveillance system" to reflect municipal monitoring capacity |
| B2.1.2 Diseases of Wild Animal | Adapted | Adapted as FOHI B2.2 "Existence of Wild animal disease surveillance system" to assess zoonotic disease prevention capacity |
| B2.2.1 Fisheries | Deleted | Captured more specifically in food security indicators; limited inland fisheries in many municipalities |
| B2.2.2 Red List Index | Deleted | Biodiversity assessments typically conducted at larger ecological zones rather than administrative boundaries |
| B3.1.1 Air quality | Deleted | Limited municipal monitoring stations; regional air patterns make municipal attribution difficult |
| B3.1.2 Climate risk index | Retained | Retained and related to FOHI C5.3 "Days with WBGT index above 25 (days/year)" to measure heat stress risk |
| B3.2.1 Biodiversity conditions | Deleted | Ecological assessments typically conducted at larger biomes rather than municipal boundaries |
| B3.2.2 Ecological Services | Deleted | Complex ecological valuation not systematically conducted at municipal level in Fukuoka |
| B3.3.1 Water Resources | Deleted | Captured by water quality indicator (A1.2); water quantity managed at watershed level across municipalities |
| B3.3.2 Acidification | Deleted | Limited direct municipal policy relevance; better addressed at regional watershed management level |
| B3.3.3 Clean water | Deleted | Near universal access to clean water in Japan with minimal municipal variation; not a differentiating factor |
| B3.3.4 Waste Reduction | Deleted | Better captured by waste recycling indicator (B3.1) which reflects municipal waste management effectiveness |
| B3.3.5 Heavy Metal Pollutants | Deleted | Site-specific contamination monitoring doesn't translate well to municipal-wide assessment metrics |
| C1.1.1 Global Connectivity | Deleted | International relationships primarily managed at national and prefectural levels in Japanese context |
| C1.1.2 One Health Association | Adapted | Adapted as FOHI C1.1 "One Health policy agreement in municipal council" to reflect formal local commitment |
| C1.1.3 One Health Forums | Adapted | Adapted as FOHI C1.2 "One Health declaration in municipal council" to assess municipal engagement level |
| C1.2.1 One Health Specialized law & Regulation | Deleted | Better captured through municipal-level policy adoption indicators (C1.1, C1.2) in FOHI |
| C1.3.1 Data Availability of National Statistical Systems | Adapted | Adapted as FOHI C1.5 "One Health website presence scale" to measure transparency and public communication |
| C1.4.1 Emergency Response Operation | Deleted | Incorporated into pet evacuation indicator (A2.2) which reflects concrete disaster preparation measures |
| C1.4.2 Risk Communication | Deleted | Better captured by One Health website presence indicator (C1.5) which reflects public information capacity |
| C1.5.1 One Health Education | Adapted | Adapted as FOHI C1.6 "Number of One Health promotion and/or education facilities" to assess local awareness efforts |
| C1.6.1 Biodiversity Protection | Deleted | Protected area designations typically managed at prefectural or national level rather than municipal level |
| C1.6.2 Social Inclusion | Deleted | Important social factor but with limited direct operational relevance to municipal One Health implementation |
| C1.7.1 Zoonotic Disease Governance | Deleted | More effectively captured through specific disease surveillance indicators in FOHI |
| C1.7.2 Climate Change Governance | Deleted | Better assessed through concrete climate mitigation and adaptation indicators in FOHI |
| C1.7.3 Government effectiveness | Deleted | Abstract governance concept better measured through specific policy implementation indicators |
| C1.8.1 One Health Official Department | Deleted | Formal departments typically exist at prefectural rather than municipal level in Japanese governance structure |
| C1.8.2 Regulatory Quality | Deleted | Abstract governance concept better measured through specific implementation indicators |
| C1.8.3 Financial Input | Adapted | Adapted as FOHI C1.3 & C1.4 indicators for One Health certified products and businesses to measure concrete initiatives |
| C2.1.1 Strategy and regulation | Deleted | Better captured through specific governance and implementation indicators at municipal level |
| C2.1.2 Monitoring and feedback | Deleted | Operational aspect better captured through specific surveillance system indicators in FOHI |
| C2.1.3 Hygiene | Deleted | Hygiene practices fairly uniform across Japanese municipalities; limited variation for meaningful comparison |
| C2.2.1 Conventional intervention | Deleted | Too broad for specific municipal assessment; better captured through specific disease control indicators |
| C2.2.2 Ecological interventions | Deleted | Specialized interventions not systematically implemented or documented across municipalities |
| C2.3.1 Vaccine coverage | Retained | Retained as FOHI C2.1 "Influenza vaccination rate for age 65+ (%)" as key preventive health measure |
| C2.3.2 Population coverage and intervention costs | Deleted | Healthcare financing primarily through national health insurance; limited municipal variation |
| C2.3.3 Inhabitants below 5 meters above sea level | Deleted | Coastal vulnerability assessment more relevant for specific coastal municipalities than prefecture-wide comparison |
| C2.4.1 Guidelines for the control and supervision of zoonotic diseases | Deleted | Better captured through specific implementation measures and surveillance indicators |
| C2.4.2 Nature reserves | Deleted | Protected areas typically designated at prefectural or national level; limited municipal control |
| C2.5.1 COVID-19 | Retained | Retained as FOHI C2.3 "COVID-19 Weekly Average Number of Cases per Sentinel Site" as critical recent zoonotic disease |
| C2.5.2 Echinococcosis | Deleted | Not endemic in Fukuoka Prefecture; zero or near-zero incidence makes it non-discriminatory |
| C2.5.3 Leishimaniasis | Deleted | Not endemic in Fukuoka Prefecture; zero or near-zero incidence makes it non-discriminatory |
| C2.5.4 Rabies | Deleted | Effectively eliminated in Japan; zero cases for decades makes it non-discriminatory |
| C2.5.5 Tuberculosis | Retained | Retained as FOHI C2.5 "Tuberculosis incidence (per 100,000 population)" as significant public health concern |
| C2.5.6 Yellow fever | Deleted | Not endemic in Japan; zero indigenous cases makes it non-discriminatory |
| C3.1.1 Food demand score | Deleted | Complex composite metric not systematically calculated at municipal level; limited policy application |
| C3.1.2 Food loss and waste | Retained | Retained as FOHI C3.1 "Food waste per capita (kg)" as key food system sustainability indicator |
| C3.1.3 Infrastructures score | Deleted | Food infrastructure assessment not systematically conducted at municipal level; too composite |
| C3.1.4 Food Aid | Deleted | Food assistance programs primarily administered at national level; limited municipal-level data |
| C3.1.5 Food Production score | Deleted | Better captured by specific agricultural indicators like arable land per capita (C3.4) |
| C3.2.1 Food Safety Governance | Adapted | Adapted as FOHI C3.2 "Good Agricultural Practices (GAP) certified producers" to measure concrete standards adoption |
| C3.2.2 Food Control and Surveillance | Retained | Retained as FOHI C3.3 "Food chain inspection implementation rate (%)" to assess safety monitoring |
| C3.2.3 Food safety evaluation | Deleted | Overlaps with food chain inspection indicator (C3.3); potential redundancy |
| C3.2.4 Foodborne illness burden | Adapted | Adapted as FOHI C2.4 "Infectious Gastroenteritis Weekly Average Cases" to track foodborne disease burden |
| C3.2.5 Safety of Livestock Production | Deleted | Better captured through livestock disease surveillance indicator (B2.1) which reflects monitoring capacity |
| C3.3.1 Food Balance | Deleted | National-level food security assessment without municipal breakdown; complex metric |
| C3.3.2 Nutrition promoting capacity | Adapted | Adapted as FOHI C3.5 "Local food promotion restaurants (per 10,000 population)" to measure healthy food access |
| C3.3.3 Nutrition score | Deleted | Dietary surveys conducted at national level; municipal-level nutrition data not systematically collected |
| C3.4.1 Famine warning | Deleted | Food security risk extremely low throughout Japan; not a meaningful indicator in this context |
| C3.4.2 Natural sources sustainability | Deleted | Resource sustainability assessments typically conducted at larger ecological zones rather than municipalities |
| C3.4.3 Economic performance index | Deleted | Better captured by specific economic indicators; composite metrics less useful for targeted improvement |
| C3.4.4 Agriculture value added per worker | Deleted | Agricultural productivity varies by crop type and scale; limited comparability across diverse municipalities |
| C3.4.5 Food price indicators | Deleted | Food pricing fairly uniform across prefecture with national retail chains; limited meaningful variation |
| C3.5.1 Investment and financial support score | Deleted | Agricultural subsidies primarily administered at national level; municipal-level data inconsistent |
| C3.5.2 Training and AI agriculture performance score | Deleted | Precision agriculture adoption uneven and early-stage in Fukuoka; not yet widespread enough for comparison |
| C4.1.1 Antimicrobial consumption surveillance | Deleted | Pharmaceutical tracking systems operate at national level; municipal-level consumption data not available |
| C4.1.2 Antimicrobial resistance status surveillance | Retained | Retained as FOHI C4.1 "AMR surveillance system (Yes/No)" as critical emerging health threat indicator |
| C4.1.3 Environmental antimicrobial resistance surveillance | Deleted | Specialized monitoring not yet implemented systematically at municipal level in Fukuoka |
| C4.2.1 National AMR capacity | Deleted | National program with limited municipal differentiation; better captured through local implementation indicators |
| C4.2.2 Technical level | Retained | Retained as FOHI C4.2 "Japan Nosocomial Infections Surveillance hospitals" to measure local AMR monitoring capacity |
| C4.2.3 National plan | Deleted | National-level policy with uniform application; not a differentiating factor among municipalities |
| C4.3.1 National law(s) for antibiotic use | Deleted | National regulations apply uniformly; not a differentiating factor among municipalities |
| C4.3.2 Antimicrobial use optimization | Deleted | Healthcare practice standardization through national guidelines; limited municipal policy variation |
| C4.3.3 Antimicrobial use control | Deleted | Pharmaceutical regulations primarily national; limited municipal policy control |
| C4.4.1 Raising awareness and understanding | Deleted | Public health education effectiveness difficult to measure consistently across municipalities |
| C4.4.2 Professional training activities in multi-sectors | Deleted | Professional development typically organized through professional associations rather than municipalities |
| C4.5.1 Carbapenems | Deleted | Specific resistance patterns tracked at hospital level; municipal aggregation challenging and potentially misleading |
| C4.5.2 Glycopeptide | Deleted | Specific resistance patterns tracked at hospital level; municipal aggregation challenging and potentially misleading |
| C4.5.3 Third-generation β-lactams-resistance for multi-species | Deleted | Specific resistance patterns tracked at hospital level; municipal aggregation challenging and potentially misleading |
| C4.5.4 Macrolides | Deleted | Specific resistance patterns tracked at hospital level; municipal aggregation challenging and potentially misleading |
| C4.5.5 Aminoglycosides | Deleted | Specific resistance patterns tracked at hospital level; municipal aggregation challenging and potentially misleading |
| C4.5.6 Quinolone | Deleted | Specific resistance patterns tracked at hospital level; municipal aggregation challenging and potentially misleading |
| C5.1.1 Greenhouse gases emissions | Retained | Retained as FOHI C5.1 & C5.2 CO2 emissions indicators as critical climate change metrics |
| C5.1.2 Energy Use | Deleted | Better reflected through emissions intensity indicators; absolute consumption less informative than efficiency |
| C5.1.3 Air quality | Deleted | Limited municipal monitoring stations; regional air patterns make municipal attribution difficult |
| C5.1.4 Natural disaster and extreme weather | Adapted | Adapted as FOHI A2.2 "Municipal regulations for pet evacuation during disasters" to assess concrete preparedness |
| C5.2.1 Air quality dalys | Deleted | Health impact assessments not conducted systematically at municipal level; methodological challenges |
| C5.2.2 Climate-related illnesses | Retained | Retained and related to FOHI C5.3 "Days with WBGT index above 25" to measure heat-related health risk |
| C5.3.1 Mitigation and adaptation achievements | Deleted | Better captured through specific indicators of climate action rather than composite assessments |
| C5.3.2 Financial support | Deleted | Climate finance tracking systems not standardized across municipalities; inconsistent reporting |
| C5.3.3 Propaganda and education | Deleted | Better captured through One Health education indicators which include climate awareness |
| C5.3.4 Forestation | Deleted | Captured by forest area indicator (A1.1) which reflects existing forest coverage |
